# Lithospermic acid, a novel KLK5 inhibitor, ameliorates rosacea by suppressing the TLR4/NF-κB signaling pathway and rectifying phenylalanine metabolism

**DOI:** 10.3389/fimmu.2026.1734997

**Published:** 2026-01-26

**Authors:** Jingang Xu, Xinyu Wang, Manyu Chen, Boya Xu, Tingting Zhang, Yao Zhang, Yueye Xu, Junjie Guo, Jingyun Xu, Yuanyuan Li, Jinhong Zhao, Hongming Zhou

**Affiliations:** 1 Department of Medical Parasitology, Wannan Medical College, Wuhu, Anhui, China; 2 School of Public Health, Wannan Medical College, Wuhu, Anhui, China; 3 School of Basic Medical Sciences, Wannan Medical College, Wuhu, Anhui, China; 4 Department of Medical Parasitology, Qiqihaer Medical College, Qiqihaer, Heilongjiang, China; 5 Anhui Provincial Key Laboratory of Biological Macro-molecules, Wuhu, Anhui, China; 6 Experimental Center of Wannan Medical College, Wuhu, Anhui, China

**Keywords:** KLK5, lithospermic acid, metabolomics, rosacea, virtual screening

## Abstract

**Background:**

Rosacea is a chronic inflammatory skin disease, with LL-37 driving inflammation. Inhibiting serine protease kallikrein-5 (KLK5) overactivity can reduce LL-37 production, thereby relieving this inflammation. Lithospermic acid (LA), a plant-derived synthetic phenolic carboxylic acid, is known for its potent anti-inflammatory and antioxidant effects. However, its effects on rosacea remain unelucidated. This study aimed to investigate the KLK5-inhibiting activity of LA to assess its therapeutic efficacy in rosacea management, along with identifying its underlying mechanisms.

**Methods:**

Herein, molecular docking and dynamic simulations of LA–KLK5 were performed via virtual screening. A rosacea mouse model was established by injecting LL-37. The therapeutic efficacy of LA was assessed based on skin erythema scores and pathological analysis (hematoxylin–eosin and toluidine blue staining). Enzyme-linked immunosorbent assay, reverse transcription-quantitative polymerase chain reaction, immunofluorescence, and Western blotting were employed to quantify relevant gene and protein expression in serum and back skin. Untargeted metabolomics was used to profile alterations in serum metabolites.

**Results:**

Notably, LA was identified as a high-affinity KLK5 inhibitor and interacted through hydrophobic and hydrogen bonds, forming a stable complex with KLK5. Furthermore, LA significantly reduced pathological changes in the skin of rosacea-affected mice, along with inhibiting the expression of matrix metalloproteinase (MMP)-9, cluster of differentiation (CD)31, CD4+, and KLK5-associated proteins in the skin tissues. Pro-inflammatory cytokines (interleukin [IL]-1β, IL-6, and tumor necrosis factor [TNF]-α) in serum and the activation of skin Toll-like receptor (TLR)2, MMP-9, KLK5, IL-1β, IL-6, and TNF-α genes were also suppressed. Additionally, LA inhibited TLR4/nuclear factor (NF)-κB-regulated inflammation by binding to KLK5, thereby improving rosacea. Metabolomics analysis identified 44 dysregulated metabolites in diseased mice, of which 28 were restored to near-normal levels following LA treatment. Pathway enrichment revealed phenylalanine metabolism regulation as a central mechanism of action of LA.

**Conclusions:**

Overall, this study, for the first time, shows that LA is a novel KLK5 inhibitor, as confirmed by molecular docking and kinetic modelling. Additionally, the results highlight that LA can ameliorate rosacea-like dermatitis through dual inhibition of KLK5 and TLR4/NF−κB signaling, while correcting metabolic disturbances, especially in phenylalanine metabolism.

## 1 Introduction

Rosacea is a chronic inflammatory skin condition characterized by chronic erythema, flushing, inflammation, papules, pustules, and pigmented changes, primarily affecting the central part of the face. Patients with rosacea often present with a severe negative impact on their state of life, self-esteem, and general well-being (1). Although the exact pathophysiology of rosacea remains elusive, the immunological dysregulation and/or neurovascular dysfunction, together with reduced skin barrier integrity, are considered the major causes. Additionally, ultraviolet (UV) exposure, climate variations, nutrition, and stress have been implicated in aggravation of the innate immune responses and/or neurovascular dysfunction (2). Cathelicidin, an antimicrobial peptide, is an indispensable part of the innate immunity (3) and has been implicated in rosacea because of serine protease kallikrein-5 (KLK5), which cleaves inactive cathelicidin to active LL-37, with high levels of KLK5 resulting in elevated LL-37 content (4). In patients with rosacea lesional skin, LL-37, which is essential to the pathophysiology of rosacea, is overexpressed (5). Reportedly, recent clinical studies have associated KLK5 inhibition with a decrease in the severity of erythema and papules (6). Therefore, drugs inhibiting the production or activity of KLK5 are expected to inhibit LL-37 expression, thereby attenuating the inflammatory cascade associated with rosacea (7).

Docking-based virtual screening (VS), a structure-based drug design approach, is widely employed in drug development research to identify new lead compounds against important therapeutic targets (8). A dataset of chemicals is docked against the three-dimensional structure of one or more receptors of interest, and the receptor–ligand binding modes are predicted to find the possible hits (9). Understanding and rationalizing the receptor–ligand interactions is a major challenge in molecular biology and essential in the development of new drugs in the field of rational drug design (10). Hence, structure-based VS was used in the present study to identify novel KLK5 inhibitors. Subsequently, molecular dynamics simulations were performed to determine the energy characteristics of drug–KLK5 interactions, thereby identifying probable binding sites and forecasting the stability of the inhibitor–protein complexes.

Lithospermic acid (LA) is a water-soluble phenolic compound extracted from the dried roots and rhizomes of *Salvia miltiorrhiza* Bge., a member of the Lamiaceae family. LA exhibits multiple biological activities, including anti-inflammatory, antioxidant, autophagy-promoting, and anti-apoptotic properties (11). Notably, topical application of 0.1% LA has been shown to ameliorate imiquimod-induced psoriatic dermatitis, restore skin barrier function, and inhibit cytokine cascades associated with the skin T-helper-17/interleukin (IL)-23 axis (12). However, its potential influence on rosacea-like lesions has not been elucidated.

The field of metabolomics, which is a relatively new field within systems biology, deals with all small molecules (<1500 Da), presenting immense potential for precision medicine. These small molecules, including sugars, lipids, fatty acids, amino acids, and nucleotides, present a wide range of shapes and physicochemical characteristics (13–15). Metabolomics exhibits notable potential for biomarker discovery owing to its dual characteristics of providing rapid responses to observable phenotypes and representing the intersection between the genome and the environment (16). Furthermore, techniques such as liquid chromatography (LC)-tandem mass spectrometry (MS) are being integrated with both non-targeted and focused metabolomics, thereby increasing the analytical potential and advancing common methods in clinical and research settings (17). Nevertheless, to the best of our knowledge, analytical findings on the different metabolites in rosacea-affected mice have not been reported previously.

This study aimed to identify novel KLK5 inhibitors through a VS approach and evaluate the potential of LA as a KLK5 inhibitor. Furthermore, the therapeutic efficacy of LA was assessed in a mouse model of LL-37-induced rosacea lesions, and the underlying mechanisms of action were elucidated.

## 2 Materials and methods

### 2.1 Structure-based VS

All VS procedures were conducted on the Yinfo Cloud Platform (http://cloud.yinfotek.com/). Compound libraries L6020, L6030, and TargetMol were screened using structure-based molecular docking approaches. The L6020 and L6030 libraries were initially processed using AutoDock Vina (18), from which the top 1000 compounds were selected for subsequent docking with DOCK 6.9 (19). The Targetmol library was screened directly using DOCK 6.9. The crystal structure of KLK5 (PDB ID: 6QFE) was retrieved from the Research Collaboratory for Structural Bioinformatics Protein Data Bank via the platform’s Processing PDB Structures widget. Protein preparation was performed with AutoDock Tools by assigning AD4 atom types and Kollman charges. Semi-flexible docking was carried out using AutoDock Vina, generating one top-binding pose per compound. Further docking with DOCK 6.9 produced 10,000 conformational orientations per ligand, which were evaluated through Grid scoring. Conformations were clustered, and only the top-scoring pose was retained. Compounds with Grid scores above −70 kcal/mol were excluded. LA was selected as the final candidate compound following manual inspection, and its binding mode was visualized via PyMOL. Subsequently, in this study, we employed Gromacs 2024.2, the industry-leading molecular dynamics simulation software, to conduct exhaustive simulations on the KLK5-LA complex obtained through molecular docking.

### 2.2 Materials

LL-37 peptide was purchased from MedChemExpress (New Jersey, USA). Doxycycline hydrochloride (DH) was obtained from Shanghai Macklin Biochemical Technology Co., Ltd. (Shanghai, China). LA and KLK5 were supplied by Shanghai TargetMol Biotech Co., Ltd. (Shanghai, China). Primary antibodies used included anti-Toll-like receptor (TLR)4 (Rabbit mAb, Cat. No. #14358, CST), anti-p65 (RelA) (Rabbit mAb, Cat. No. #8242, CST), and anti-phospho-p65 (p-p65) (Ser536) (Rabbit mAb, Cat. No. #3033, CST). Secondary Goat Anti-Rabbit IgG was purchased from Beijing Labgic Technology Co., Ltd. (Haimen, China).

### 2.3 Animals and treatment

Herein, 49 female BALB/c mice (6–8 weeks old, specific pathogen-free grade) were obtained from Henan Skebes Biotechnology Co., Ltd. (License No. SCXK(Yu)2020-0005). All animal procedures were approved by the Animal Ethics Committee of Wannan Medical College (Approval No. SUCMDL20220805003).

Notably, mice were randomly divided into the following seven groups (n = 7 per group): Control (phosphate-buffered saline [PBS]), Model (LL-37), DH (30 mg/kg/d), LA groups (intraperitoneal injection: 20, 40, and 60 mg/kg/d), and LL-37 plus high-dose LA together with KLK5 (60 mg/kg/d + 2 μg). Sample size selection was based on power analysis and previous similar research, ensuring adequate statistical strength to identify treatment differences at α = 0.05 and 80% power.

**Figure 1:**
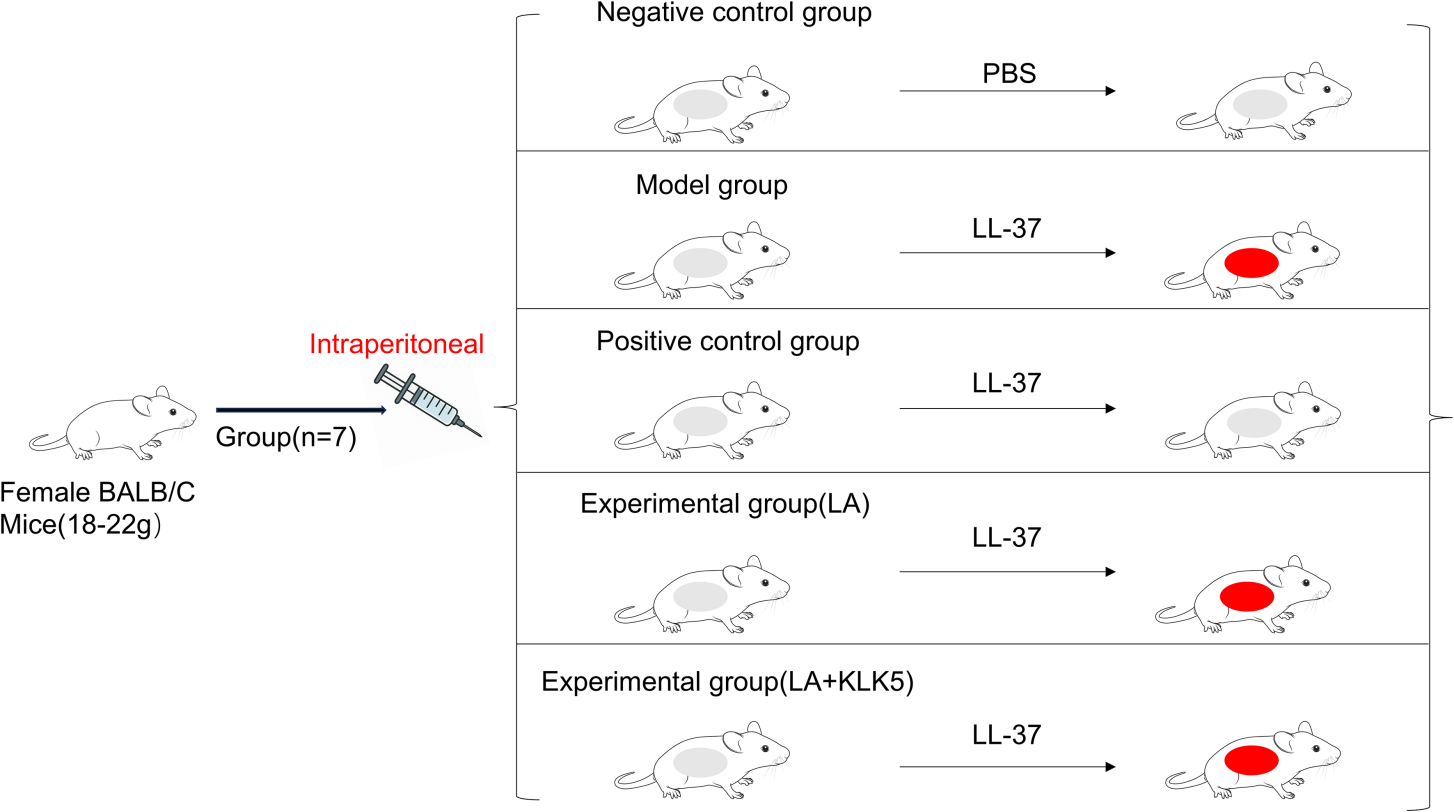
A schematic depiction of mouse modeling, drug delivery, and skin measurements.

All mice were shaved over a 3 × 3 cm dorsal area 24 h before treatment. Rosacea model and LA-treated groups received subcutaneous injections of LL-37 (40 μL, 320 μM) twice daily for 2 d, following previously validated protocols. Control mice were subcutaneously injected with 40 μL PBS. The DH group received intraperitoneal injections (30 mg/kg/d) every 24 h, starting 12 h after LL-37 exposure, for a total of two doses. LA was administered intraperitoneally at 20, 40, or 60 mg/kg/d for 2 d. For the LA + KLK5 treatment, KLK5 (2 μg) was injected subcutaneously into the dorsal skin every 24 h for two injections (**Figure 1A**). After 12 h of the final injection, dorsal skin lesions were photographed, and rosacea-like symptoms were assessed based on erythema intensity and size (20). Erythema was scored from 0 to 4, as follows: 0, no redness; 1, slight redness; 2, mild redness; 3, distinct and clearly visible redness; and 4, pronounced redness with sharp margins (20).

### 2.4 Histopathological analysis and immunofluorescence

Dorsal skin samples were fixed in 4% paraformaldehyde, embedded in paraffin, and sectioned at 5 μm thickness. Sections were pretreated, dehydrated, mounted, and stained using hematoxylin and eosin (HE) and toluidine blue (TB). A vertical optical microscope (Olympus BX53, Japan) was used to examine the histomorphology. For immunofluorescence staining, Rat anti-cluster of differentiation (CD)4 (1:100, eBioscience), Rat anti-CD31 (1:100, eBioscience), Rat anti-matrix metalloproteinase (MMP)-9 (1:100, eBioscience), and Rat anti-KLK5 (1:100, eBioscience) antibodies were applied to tissue sections. Finally, stained tissue sections were analyzed using an immunofluorescence microscopy system.

### 2.5 Enzyme-linked immunosorbent assay

Serum concentrations of IL-1β, IL-6, and tumor necrosis factor (TNF)-α were quantified using ELISA kits (Ruixin Biological Company, Quanzhou, China) following the manufacturer’s instructions. Absorbance was measured at 450 nm using a microplate reader (Molecular Devices, USA).

### 2.6 Reverse transcription-quantitative polymerase chain reaction assay

Total RNA was extracted from dorsal skin tissue using TRIzol reagent (ShareBio), and RNA concentration and purity were quantified according to the manufacturer’s instructions. Following the reverse transcription, complementary DNA was amplified using Taq DNA polymerase. PCR amplification consisted of 40 cycles performed on an Applied Biosystems system (Roche LightCycler 96), under the following conditions: denaturation at 95°C for 30 s and annealing at 60°C for 30 s per cycle. Melting curve analysis was conducted to verify amplification specificity. Relative gene expression was calculated using the 2^−ΔΔCT^ method. Skin tissue was used as an internal control using glyceraldehyde-3-phosphate dehydrogenase (GAPDH). Primer sequences are listed in **Table 1**.

**Table 1:** Forward and reverse primer sequences for GAPDH, IL-1β, IL-6, TNF-α, TLR2, MMP-9, and KLK5.

Gene	Primers	Sequence (5’-3’)
GAPDH	Forward	ACCACAGTCCATGCCATCAC
	Reverse	GTGAGGGAGATGCTCAGTGT
IL-1β	Forward	AAAAAAGCCTCGTGCTGTCG
	Reverse	GTCGTTGCTTGGTTCTCCTTG
IL-6	Forward	TGATGGATGCTACCAAACTGGA
	Reverse	TGTGACTCCAGCTTATCTCTTGG
TNF-α	Forward	CAGGCGGTGCCTATGTCTC
	Reverse	CGATCACCCCGAAGTTCAGTAG
TLR2	Forward	TCTAAAGTCGATCCGCGACAT
	Reverse	CTACGGGCAGTGGTGAAAACT
MMP-9	Forward	GGTCCTCACCATGAGTCCCT
	Reverse	AGACCACAAAAGTCGGCTGG
KLK5	Forward	CAGAACCACTTAGCCTCGAC
	Reverse	AGGGTAGCCATTGCCCATTT

### 2.7 Western blotting

The protein expression of TLR4, p65, and p-p65 was analyzed by Western blotting. Herein, dorsal skin tissues were homogenized in the radioimmunoprecipitation assay buffer, supplemented with 1% phenyl methane sulfonyl fluoride, and centrifuged at 12,000 rpm and 4°C for 10 min. Total protein concentration was determined using a bicinchoninic acid assay kit. Equal amounts of protein were separated through 10% sodium dodecyl sulfate-polyacrylamide gel electrophoresis and transferred onto polyvinyl difluoride membranes (Beyotime, China). Membranes were blocked and incubated overnight at 4°C with primary antibodies diluted 1:1000. After being washed thrice in Tris-buffered saline with Tween 20, membranes were incubated with horseradish peroxidase–conjugated secondary antibodies for 1 h at room temperature. Target protein bands were visualized using enhanced chemiluminescence detection, and data analysis was performed by computerized densitometry software ImageJ.

### 2.8 Metabolomics

Serum metabolomic profiling was performed using LC-MS. Serum samples stored at −80°C were thawed on ice, vortexed, and centrifuged at 12,000 ×*g* for 1 min at 4°C. Quality control and system suitability test samples were prepared. Metabolites were extracted using pre-cooled methanol (−80°C). An internal standard solution was added, followed by vortexing, shaking, and incubation at −20°C for 30 min. After centrifugation at 14,000 ×*g* for 10 min at 4°C, the resulting supernatant was freeze-dried and reconstituted in 10% aqueous methanol for LC-MS analysis.

Chromatographic separation was performed on a Waters ACQUITY BEH C18 column (1.7 µm, 2.1 mm × 100 mm). The following two mobile-phase systems were employed: (1) 0.1% formic acid in water and 0.1% formic acid in acetonitrile/methanol (40/60) for both positive and negative ion modes; (2) 6.5 mM ammonium bicarbonate in water and methanol for negative ion mode detection. MS analysis was conducted using a Q Exactive HF-X instrument with a resolution of 60,000 and a scan range of 60–900 m/z.

For non-targeted metabolomic analysis, differential metabolites were identified using univariate statistical tests (t-test or Wilcoxon test) combined with multivariate orthogonal partial least squares–discriminant analysis (OPLS-DA) modeling (VIP > 1 and p < 0.05). Model performance was evaluated using R2X, R2Y, and Q2 values to assess explanatory and predictive performance. To identify enriched metabolic pathways, Kyoto Encyclopedia of Genes and Genomes (KEGG) pathway enrichment and metabolite set enrichment analysis (MSEA) were performed using the corto package (500 permutations). Regulatory networks were visualized with the ggraph package. All analyses were conducted in R (v4.3.2) using the ropls, corto, and ggplot2 packages.

### 2.9 Statistical analyses

Data are expressed as mean ± standard error of the mean. Statistical analyses were performed using ImageJ software (National Institutes of Health, USA) and GraphPad Prism 8.0 (GraphPad Software, Inc., San Diego, CA, USA). The Shapiro–Wilk test was used to assess data normality. For normally distributed data, comparisons between two groups were made using Student’s t-test, and multiple groups were analyzed using one-way analysis of variance. For non-normally distributed data, the Kruskal–Wallis test was used. Correlations between numerical variables were calculated using Pearson or Spearman correlation coefficients, depending on data distribution. Statistical significance was set at p < 0.05.

## 3 Results

### 3.1 Virtual screening

**Figure 2:**
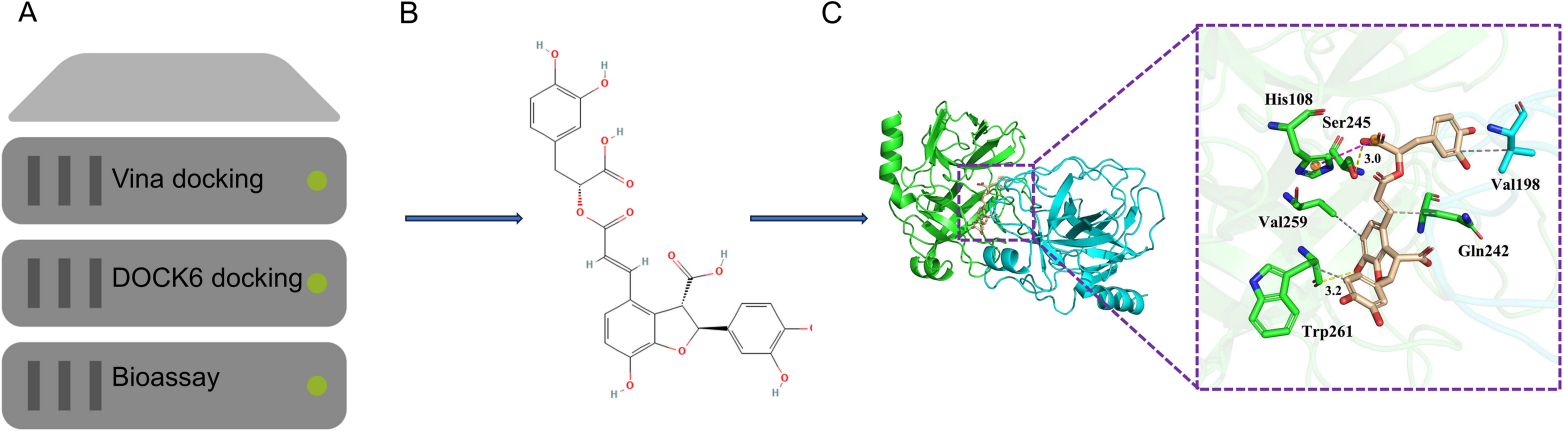
Flowchart depicting the discovery of lithospermic acid (LA), a novel kallikrein-5 (KLK5) inhibitor. **(A)** Virtual screening flowchart; **(B)** Chemical structure of LA; **(C)** LA binding to KLK5; green cartoon, A chain and cyan cartoon, B chain of KLK5. The green and cyan sticks indicate the critical residues, and the wheat-colored sticks indicate LA. Gray dashed lines indicate hydrophobic interactions, whereas yellow dashed lines indicate hydrogen bonds.

VINA (Virtual Screening Tool) and DOCK 6.9 (Docking and Scoring Function) were employed for the primary and secondary screenings, respectively (**Figure 2A**). Fifty potential seed compounds were identified through manual evaluation of binding mechanisms for compounds with a Grid Score below −70 kcal/mol. Preliminary testing revealed that LA (28831-65-4) could bind to KLK5 and inhibit its enzymatic activity (**Figure 2B**). Compounds exhibiting Grid_Score values below −70 kcal/mol, a threshold generally indicative of strong binding, confirmed that LA showed substantial affinity for KLK5, as supported by scoring analysis (**Table 2**). This improved binding was attributed to LA’s relatively large molecular size, which enabled the formation of additional hydrophobic contacts within the protein. The overall binding affinity was determined based on van der Waals (Grid_vdw) and electrostatic (Grid_es) interactions, with the van der Waals forces being most dominant. Nonetheless, electrostatic contributions were considerably significant, indicating the presence of many polar contacts.

**Table 2:** Molecular docking score of KLK5 with LA (in kcal/mol).

Compounds	Grid_Score	Grid_vdw	Grid_es	Int_energy
LA(28831-65-4)	-100.29	-90.50	-9.79	18.10

The small molecule was observed to bind between the two polypeptide chains of KLK5, establishing tight interactions through hydrophobic forces, hydrogen bonds, and salt bridges. Specifically, the benzene ring of LA formed hydrophobic interactions with residues Gln242, Val259, and Trp261 of chain A, and Val198 of chain B. These hydrophobic interactions resulted in substantial van der Waals forces (Grid_vdw = −90.50 kcal/mol) to facilitate the binding of small molecules to proteins. Furthermore, LA formed hydrogen bonds with Ser245 and Trp261 of chain A at distances of 3.0–3.2 Å, and a salt bridge with His108. This salt bridge substantially increased the electrostatic interaction between both entities (Grid_es = −9.79 kcal/mol). The combined influence of these binding forces yielded a total binding affinity score of −100.29 kcal/mol, enabling LA to effectively inhibit KLK5 activity (**Figure 2C**). Based on the molecular docking results, this study selected the LA-KLK5 complex for molecular dynamics simulation analysis. We compared the mean values of RMSD, Rg, RMSF and SASA for LA_KLK5 via molecular dynamics simulations, yielding: RMSD = 0.490 nm, Rg = 2.521 nm, RMSF = 0.336 nm, SASA = 220.01 nm².

### 3.2 LA dose escalation study reveals its inhibitory effect on LL-37-induced phenotype in rosacea-like mice

To determine the optimal therapeutic dose for rosacea-like lesions in mice, LA was administered at three dose levels: 20 mg/kg/d (low, L), 40 mg/kg/d (medium, M), and 60 mg/kg/d (high, H). After treatment, skin erythema severity and affected area were assessed. In the LL-37 + H group, only 14% (1/7) of mice showed mild erythema. In contrast, 71% (4/7) of mice in the LL-37 + M group exhibited mild to moderate erythema, and all mice in the LL-37 + L group displayed mild to moderate erythema. Similarly, 14% (1/7) of mice in the DH group exhibited mild erythema. Treatment with 60 mg/kg/d LA significantly reduced erythema in rosacea mice, as evidenced by the considerably reduced average erythema score and area in the LA 60 group, compared with that in the LA 20 group (p < 0.001). The high-dose group showed the most pronounced effect.

**Figure 3:**
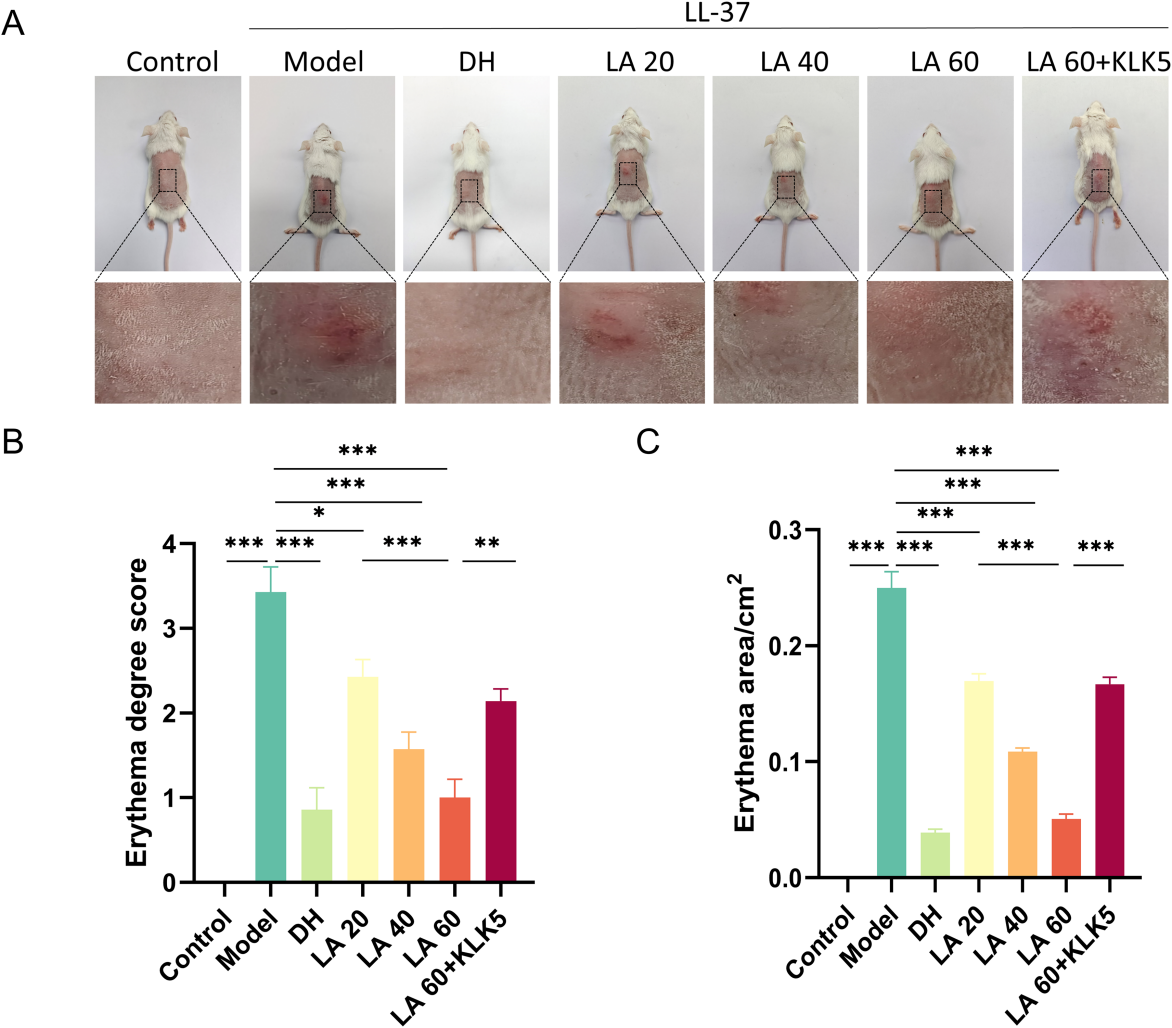
Effects of lithospermic acid on rosacea-like skin lesions in LL-37-induced mice. **(A)** Gross photographs, representative of seven mice; **(B)** Erythema area of seven mice in different groups; **(C)** Erythema score of seven mice in different groups. Data are presented as mean ± standard error of the mean, *p < 0.05, **P<0.01, ***p < 0.001. DH, doxycycline hydrochloride.

To evaluate LA’s therapeutic efficacy, a rosacea mouse model was developed using LL-37 (**Figure 1**), as previously described (21). Erythema developed on the dorsal skin of mice, characterizing rosacea-like pathology. Mice treated with LA or DH demonstrated marked improvement in symptoms, suggesting that LA’s inhibitory effect is comparable to that of DH (**Figure 3A**). In LA-treated mice, both the affected area and erythema score were significantly reduced (p < 0.05 or p < 0.001). Mice were given LA 60 treatment and then subcutaneous KLK5 injections to investigate the function of KLK5 in LA-mediated improvement. Compared with the mice treated with LA 60, the mice treated with LA 60 + KLK5 exhibited larger erythema areas and higher erythema scores (p < 0.01 or p < 0.001), indicating aggravated dorsal erythema (**Figures 3B, C**). Overall, these findings confirm that LA treatment effectively improves the skin phenotype in rosacea-like mice.

### 3.3 LA inhibits damage in rosacea-like mouse pathology

**Figure 4:**
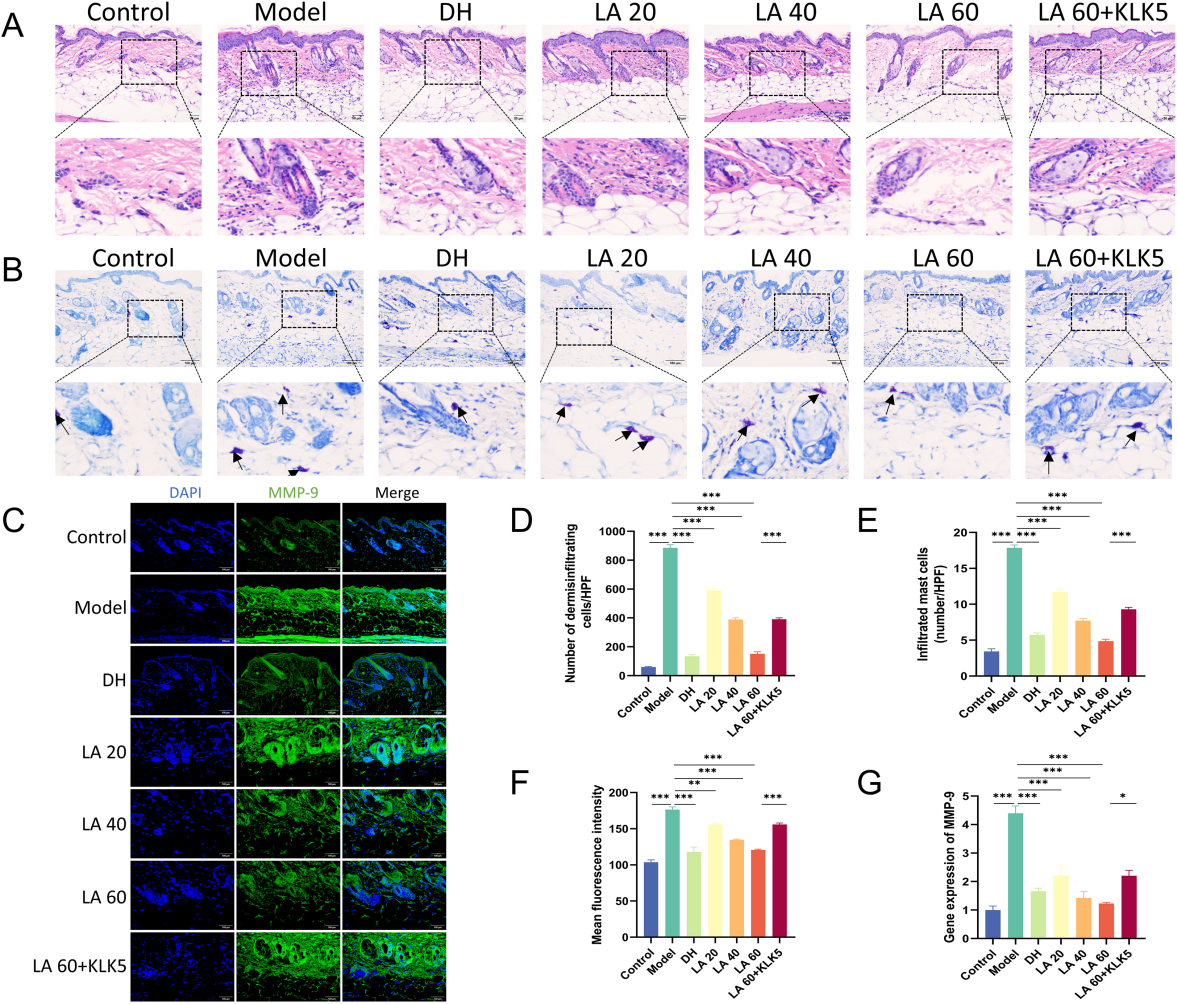
Effect of lithospermic acid (LA) on pathological features of rosacea-like mouse skin. **(A)** Hematoxylin and eosin staining for histological analysis of rosacea-like lesions (magnification, 400×; scale bar, 100 μm); **(B)** Toluidine blue (TB) staining, black arrows indicate mast cells (magnification, 400×; scale bar, 100 μm); **(C)** Immunostaining of MMP-9 in skin lesions from control mice and LL-37-induced mice (scale bar, 50 μm); **(D)** Quantification of inflammatory cells; **(E)** Mast cell counts from TB-stained sections; **(F)** Quantification of fluorescence intensity of dermal MMP-9-positive T cells from control and LL-37-induced mice treated with phosphate-buffered saline or LA (n = 7 per group); **(G)** Expression of MMP-9 determined by reverse transcription-quantitative polymerase chain reaction. Data are presented as mean ± standard error of the mean, *p < 0.05, **p < 0.01, ***p < 0.001. MMP-9, matrix metallopeptidase 9.

HE staining revealed increased epidermal thickness and enhanced inflammatory cell accumulation (**Figure 4A**). LA-treated rosacea mice exhibited a marked dose-dependent suppression of inflammatory cells (**Figure 4D**). Reportedly, in rosacea lesions, substantial innate immune cell infiltration, including that of mast cells (MCs), has been recorded (22). Consequently, MC accumulation in the dorsal skin was assessed using TB staining. Results revealed a marked increase in MC numbers following LL-37 treatment (**Figure 4B**). In LA-treated mice, MC numbers significantly declined in a dose-dependent manner (**Figure 4E**). Furthermore, MC numbers were markedly suppressed in DH-treated mice, mirroring the effect observed in LA-treated mice.

Activated MCs contribute to inflammatory amplification by producing MMP-9, which in turn promotes LL-37 production (23). Therefore, immunofluorescence analysis was performed to assess MMP-9 expression. The model group exhibited significantly higher mean fluorescence intensity of MMP-9 than that of the controls (p < 0.001). LA treatment led to a pronounced, dose-dependent decrease in MMP-9 protein expression relative to the model group (p < 0.01 or p < 0.001) (**Figures 4C, F**). Furthermore, these findings were corroborated by RT-qPCR results, which showed a dose-dependent downregulation of MMP-9 expression following LA treatment and increased MMP-9 messenger RNA (mRNA) in the model group compared with that in the controls (**Figure 4G**).

Additionally, histopathological staining showed that mice in the LA 60 + KLK5 group exhibited greater inflammatory and MC infiltration than those in the LA 60 group (p < 0.001). RT-qPCR and immunofluorescence analyses revealed significantly higher MMP-9 mRNA and protein levels in the LA 60 + KLK5 group (p < 0.05 or p < 0.001). These results indicate that KLK5 exacerbated rosacea-like inflammation by counteracting the anti-inflammatory effects in the LA 60 group. Overall, these findings suggest that LA alleviates rosacea-like pathological changes in mice by suppressing inflammatory cell and MC infiltration in a dose-dependent manner.

### 3.4 LA reduces LL-37-induced skin angiogenesis in rosacea mice

**Figure 5:**
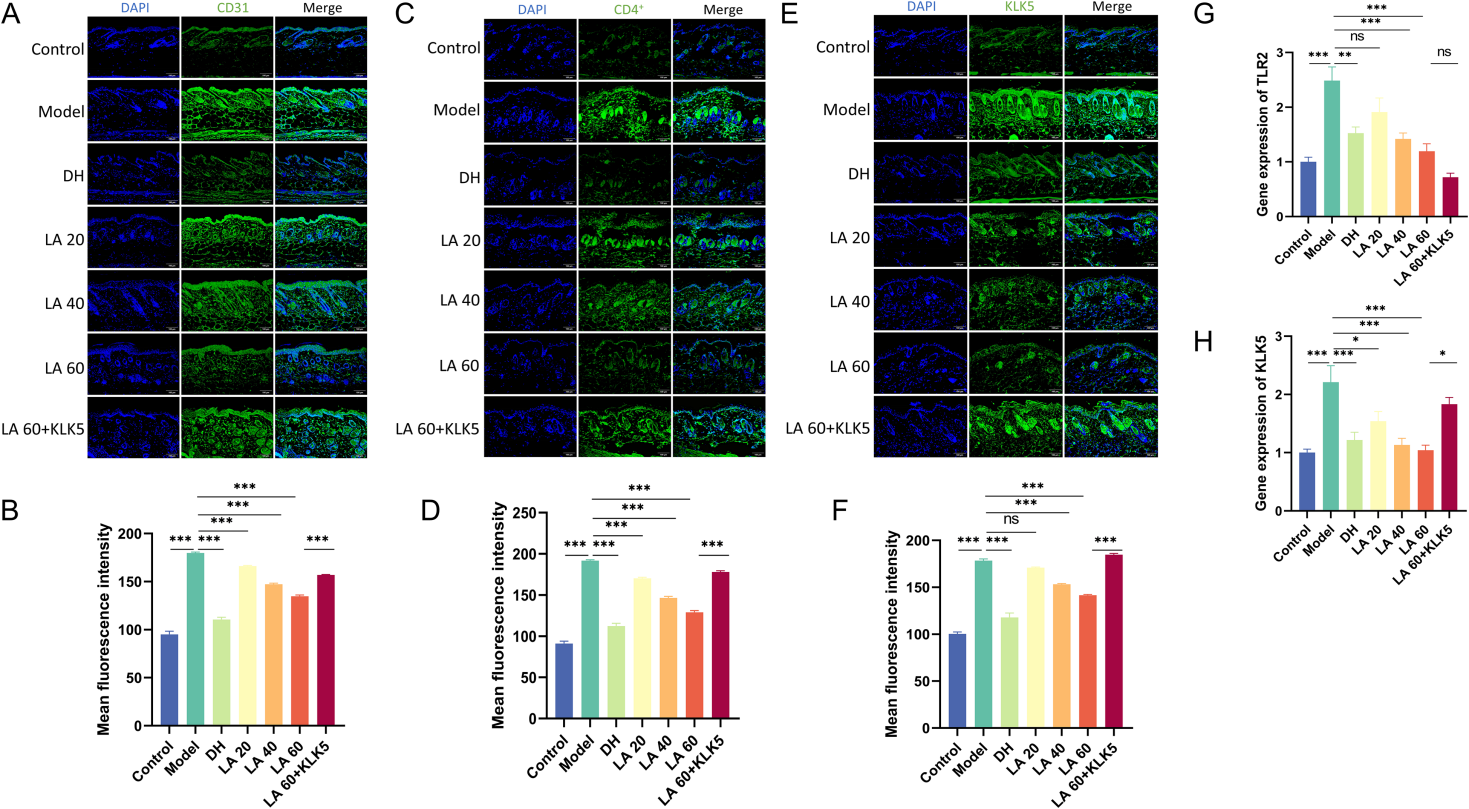
Effects of lithospermic acid on dermal cluster of differentiation (CD)31, CD4, and kallikrein-5 (KLK5) levels in a mouse model. **(A)** CD31 immunostaining of skin lesions from control and LL-37-induced mice (scale bar, 50 μm); **(B)** Quantification of dermal CD31 fluorescence intensity (n = 7 per group); **(C)** CD4 immunostaining of skin lesions from control and LL-37-induced mice (scale bar, 50 μm); **(D)** Quantification of dermal CD4 fluorescence intensity (n = 7 per group); **(E)** Immunostaining of KLK5 in skin lesions from control and LL-37-induced mice (scale bar, 50 μm); **(F)** Quantification of dermal KLK5 fluorescence intensity (n = 7 per group); **(G–H)** Toll-like receptor 2 and KLK5 expression by reverse transcription-quantitative polymerase chain reaction; Data are presented as mean ± standard error of the mean, *p < 0.05, **p < 0.01, ***p < 0.001. NS, not significant (p > 0.05).

Reportedly, neurovascular dysregulation plays a crucial role in the pathophysiology of rosacea, potentially leading to the characteristic flushing and persistent erythema observed in the skin (24). To assess cutaneous vascular changes, CD31 (a vascular marker) immunostaining was performed. Active angiogenesis was indicated by the considerably higher mean CD31 fluorescence intensity (p < 0.001) in LL-37-treated mice. In contrast, LA administration markedly decreased CD31 intensity in a dose-dependent manner (p < 0.01 or p < 0.001), demonstrating suppression of skin angiogenesis. However, compared to the LA 60 group, the LA 60 + KLK5 group showed significantly greater CD31 infiltration (p < 0.001), indicating that KLK5 reversed the inhibitory effect of LA. These results suggest that LA inhibits angiogenesis in rosacea by targeting KLK5 (**Figures 5A, B**).

### 3.5 LA inhibits CD4^+^ T cell infiltration in LL-37-induced cutaneous inflammation

The presence of CD4^+^ T cell infiltration within rosacea-affected skin lesions constitutes a significant immunological marker, strongly suggesting that the adaptive immune system is profoundly involved in the chronic inflammatory process of the disease (25). Immunofluorescence analysis revealed significantly higher mean CD4^+^ T cell fluorescence intensity in the model group compared with that in the controls (p < 0.001). LA treatment reduced CD4^+^ T cell infiltration in a dose-dependent manner (p < 0.001), alleviating rosacea-like inflammation. In contrast, mice in the LA 60 + KLK5 group showed substantially higher CD4^+^ T cell infiltration than those in the LA 60 group (p < 0.001) (**Figures 5C, D**).

### 3.6 LA attenuates inflammation in rosacea-like mice by inhibiting KLK5

The inhibitory effect of LA on KLK5 was evaluated through immunofluorescence. The model group exhibited significantly higher KLK5 fluorescence intensity compared to controls (p < 0.001) (**Figures 5E, F**). Both LA and DH treatments significantly reduced KLK5 expression (p < 0.001 or not significant), with LA showing a dose-dependent inhibitory effect. RT-qPCR results support these findings, highlighting that LA markedly downregulated KLK5 expression in rosacea-like mice in a dose-dependent manner (p < 0.05 or 0.001).

Keratinocytes in rosacea lesions express elevated levels of the pattern recognition receptor TLR2and cathelicidin antimicrobial peptides. Activation of TLR2 by triggers such as UV radiation, Demodex infestation, and microbial stimuli increases production of the serine protease KLK5. KLK5 cleaves cathelicidin into LL-37 and smaller peptides, which promote angiogenesis and pro-inflammatory processes (26).

RT-qPCR analysis showed that LA treatment significantly decreased TLR2 expression dose-dependently (p < 0.001 or not significant), suggesting that LA partially downregulates KLK5 expression by inhibiting TLR2 (**Figure 5G**). To further confirm the relationship between KLK5 and LA activity, mice in the LA 60 group received subcutaneous KLK5 injections. RT-qPCR and immunofluorescence results indicated a significantly increased KLK5 gene and protein expression (p < 0.05 or p < 0.001), showing that elevated KLK5 reversed the therapeutic effect of high-dose LA in rosacea-affected mice (**Figures 5F, H**). Collectively, these findings demonstrate that LA mitigates rosacea-like inflammation by suppressing TLR2-mediated KLK5 expression.

### 3.7 LA inhibited the activity of the TLR4/nuclear factor -κB pathway in rosacea-like mice

**Figure 6:**
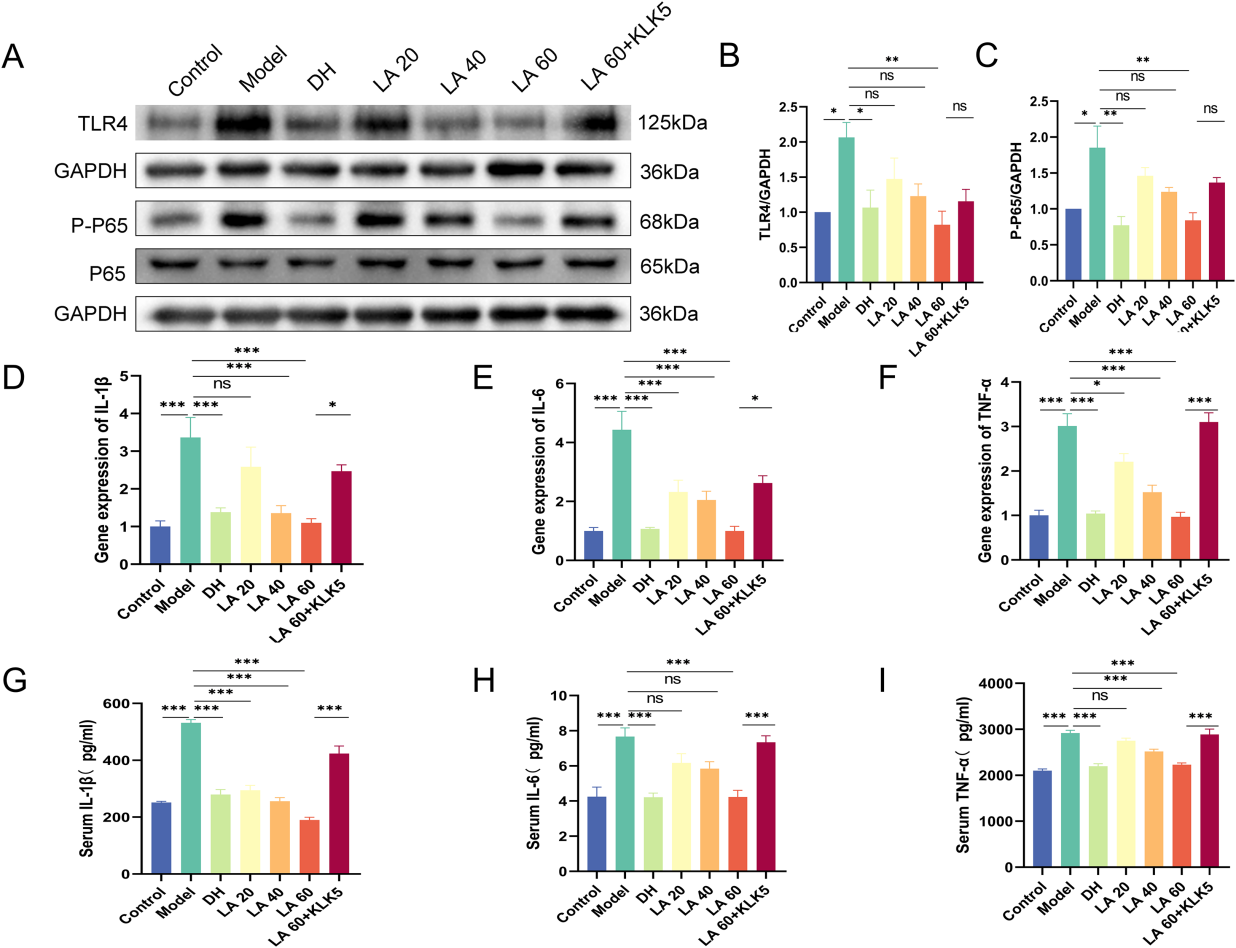
Effect of lithospermic acid on toll-like receptor (TLR)4/nuclear factor (NF)-κB pathway activity in rosacea-like mouse skin. **(A)** Western blot analysis of TLR4, phosphor-P65 (p-p65), and glyceraldehyde-3-phosphate dehydrogenase; **(B)** Ratio of TLR4/GAPDH (n = 3); **(C)** Ratio of p-p65/GAPDH (n = 3). **(D–F)** Reverse transcription-quantitative polymerase chain reaction analysis of IL-1β, IL-6, and tumor necrosis factor (TNF)-α expression; **(G–I)** Enzyme-linked immunosorbent assay of IL-1β, IL-6, and TNF-α serum levels. Data are presented as mean ± standard error of the mean, *p < 0.05, **p < 0.01, ***p < 0.001. NS, not significant (p > 0.05).

A crucial receptor in the innate immune system, toll-like receptor 4 (TLR4) identifies pathogen-associated molecular patterns, including bacterial lipopolysaccharides. When TLR4 is activated, its adaptor proteins set off a series of downstream signaling events that eventually activate nuclear factor κB (NF-κB) (27); therefore, the effect of LA on the TLR4/NF-κB signaling pathway was investigated. The LL-37-induced rosacea mouse model exhibited significantly increased expression of TLR4 and p-p65 (p < 0.05) (**Figures 6A–C**). In contrast, LA treatment significantly decreased both TLR4 expression and NF-κB phosphorylation in a dose-dependent manner. Notably, the LA 60 + KLK5 group displayed significantly higher TLR4 and p-p65 expression than the LA 60 group (NS), indicating that KLK5 reactivation reversed LA-mediated inhibition. TLR4 activates the NF-κB pathway; hence, it can upregulate inflammatory mediators such as IL-1β, IL-6, and TNF-α (28, 29). Inflammatory diseases have been connected to an excess of these cytokines (30).Therefore, RT-qPCR was used to determine cytokine expression. LL-37 treatment led to significant elevations in IL-1β, IL-6, and TNF-α mRNA levels (p < 0.001), whereas LA administration decreased these levels in a dose-dependent manner (**Figures 6D–F**). ELISA results further confirmed these findings, with LL-37-treated mice exhibiting higher levels of IL-1β, IL-6, and TNF-α (p < 0.001). These were subsequently suppressed after LA treatment in a dose-dependent manner (**Figures 6G–I**). Cytokine expression in both skin and serum was significantly higher in the LA 60 + KLK5 group than that in the LA 60 group (p < 0.05 or p < 0.001). Collectively, these findings indicate that LA attenuates rosacea-like inflammation by suppressing the TLR4/NF-κB signaling pathway by inhibiting KLK5.

### 3.8 LA modulates serum metabolomics in rosacea mice

An untargeted metabolomic approach was subsequently employed to investigate how LA modulates metabolic pathways. Log-transformed raw data were analyzed to evaluate group separation, followed by one-way statistical tests (t-test or Wilcoxon test) and multifactorial analysis with OPLS-DA using the R package ropls. Differential metabolites were identified using thresholds of VIP > 1.0 and p < 0.05.

**Figure 7:**
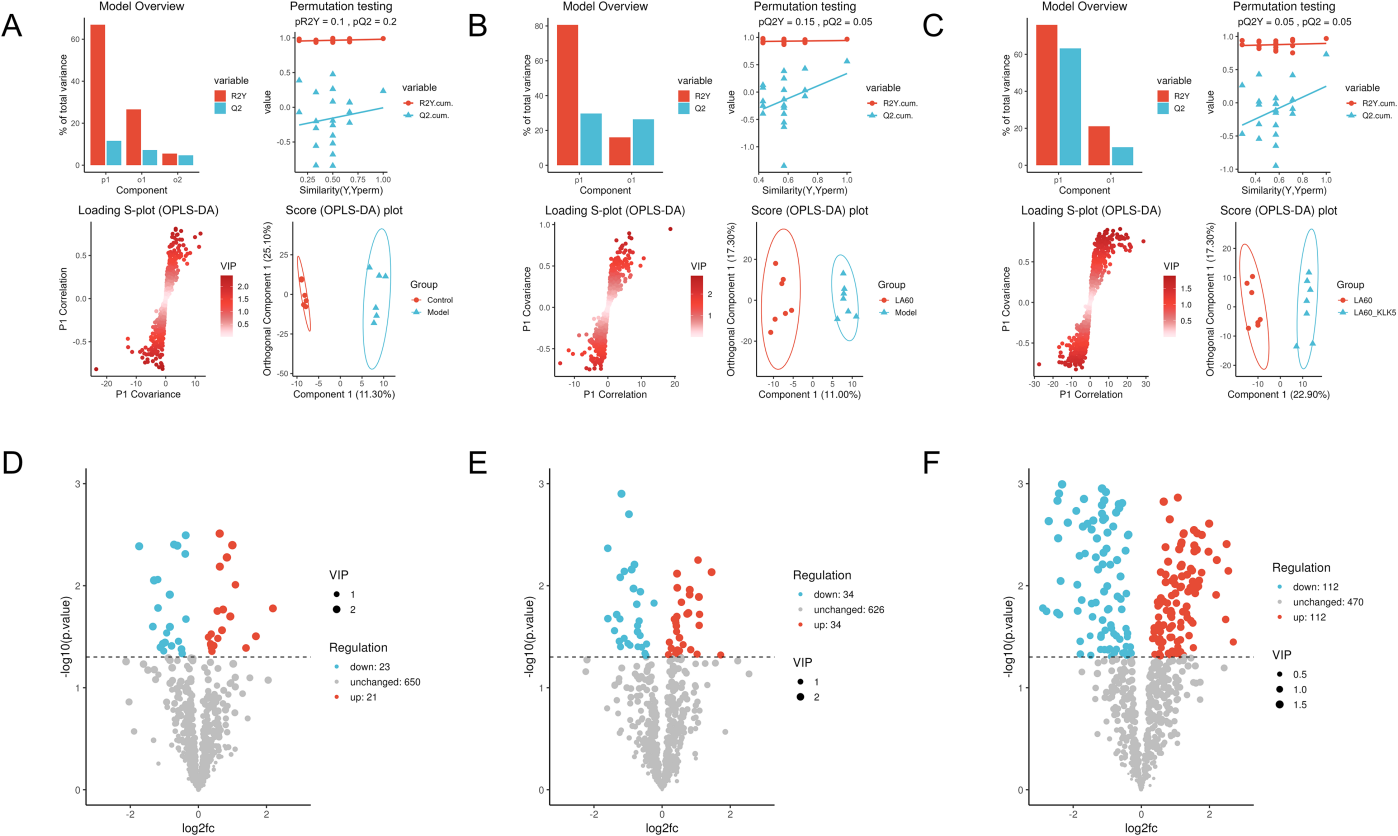
Effect of lithospermic acid on serum metabolomics in rosacea-like mice. **(A–C)** Orthogonal partial least squares–discriminant analysis; **(D–F)** Volcano plots of differential metabolites. Data are presented as mean ± standard error of the mean.

OPLS-DA analysis revealed clear separation between the experimental groups, indicating significant metabolic distinctions. Specifically, separations were observed between the Control and Model groups (R2X = 0.34, R2Y = 0.92, Q2 = 0.19), between the Model and LA 60 groups (R2X = 0.28, R2Y = 0.96, Q2 = 0.56), and between the LA 60 and LA 60+KLK5 groups (R2X = 0.40, R2Y = 0.96, Q2 = 0.73) (**Figures 7A–C**). Volcano plots visualized the distribution of differential metabolites. Notably, 44 metabolites were identified between the Control and Model groups (21 upregulated, 23 downregulated. LA treatment normalized 28 of these metabolites back to normal levels. In contrast, 68 different metabolites were found between the Model and LA 60 groups, (34 upregulated, 34 downregulated). Comparatively, 224 differential metabolites were identified between the LA 60 and LA 60 + KLK5 groups, with 112 being upregulated and 112 being downregulated in the LA 60 group. Phenylalanine was found to be predominant among these metabolites (**Figures 7D–F**).

**Figure 8:**
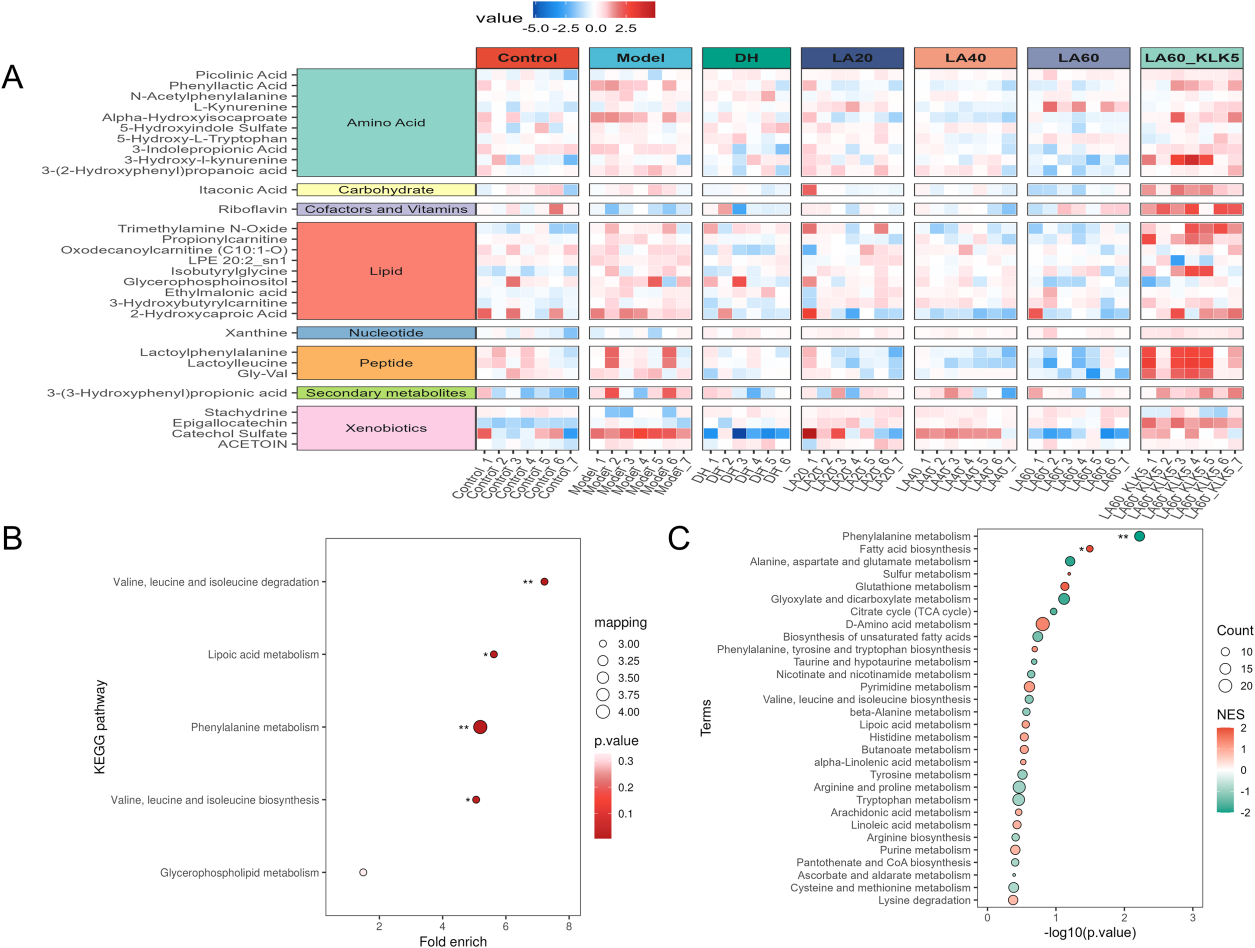
Effect of lithospermic acid on serum metabolomics in rosacea-like mice. **(A)** Heat map of differential metabolites; **(B)** Kyoto Encyclopedia of Genes and Genomes pathway enrichment analysis; **(C)** Metabolite set enrichment analysis. *P<0.05; **P<0.01.

Heatmap analysis of univariate and multivariate data identified 30 key metabolites, mainly involved in the phenylalanine metabolism pathway (e.g., phenyllactic acid, 3-(2-hydroxyphenyl)propanoic acid, 3-(3-hydroxyphenyl)propionic acid, N-acetylphenylalanine, and lactoylphenylalanine) (**Figure 8A**). Pathway enrichment analysis using Fisher’s exact test revealed significant enrichment in phenylalanine metabolism, lipoic acid metabolism, and degradation and biosynthesis of valine, leucine, and isoleucine (**Figure 8B**).

Furthermore, MSEA analysis of all identified metabolites and KEGG pathways indicated enrichment in the metabolism of phenylalanine, arginine, proline, D-amino acids, and tryptophan (**Figure 8C**). Overall, integrated KEGG and MSEA data show that LA may alleviates rosacea by modulating the metabolism of phenylalanine.

## 4 Discussion

In recent years, the prevalence of rosacea has notably increased. It is now recognized as a chronic inflammatory skin disorder affecting approximately 1–20% of the global population. Clinically, rosacea is characterized by persistent facial erythema, telangiectasia, papules, pustules, and ocular symptoms. Its pathophysiology is complex, involving interactions among genetic, environmental, immunological, microbial, and neurovascular variables (31). LA is a naturally occurring compound derived from *S. miltiorrhiza*, with well-documented bioactive properties. Previous studies have shown that LA exhibits both antioxidant and anti-inflammatory effects across a range of disease models (32, 33). Despite these promising findings, the therapeutic potential of LA in rosacea has not been fully explored.

In the present study, LA was identified as an effective and selective KLK5 inhibitor via structure-based VS. The binding analysis revealed that LA exhibited a high binding affinity for KLK5 (–100.29 kcal/mol), supporting its inhibitory actions on KLK5. These results underscore the potential of LA as a promising lead compound for developing novel therapeutic strategies against rosacea.

A key pathological feature of rosacea is the overexpression of the antimicrobial peptide LL-37 in the affected skin of patients (34). LL-37 plays multifaceted roles in regulating inflammation, angiogenesis, and immune responses. Specifically, it activates keratinocytes, neutrophils, macrophages, and MCs, leading to increased MMP production, angiogenesis, and pro-inflammatory cytokines (31). In this study, LL-37 exposure in mice resulted in substantial infiltration of inflammatory cells and MCs within mouse skin tissue, along with enhanced cutaneous angiogenesis and elevated CD4+ T-cell infiltration, which exacerbate rosacea-like skin lesions. Following LA treatment, these pathological changes were significantly reversed, including reduced inflammatory cell and MC infiltration, inhibition of skin neovascularization, and decreased CD4+ T-cell accumulation. This indicates that LA effectively alleviates LL-37-induced rosacea-like symptoms. Moreover, the therapeutic efficacy of LA exhibited a dose-dependent relationship, with its ameliorative action becoming more pronounced as the dosage increased. DH is a commonly used medication to treat rosacea (35). It was chosen as a positive treatment agent in this study, and it was found that the LA 60 group did not differ statistically significantly from the DH group regarding erythema severity or associated clinical indices. This finding further supports the potential of LA as a viable and effective therapeutic candidate for rosacea treatment.

TLR2, a key pattern recognition receptor, is markedly overexpressed in the keratinocytes of patients with rosacea, rendering their skin more sensitive to external stimuli. Furthermore, KLK5 is released when TLR2 is activated (26). KLK5 is initially inactive, but serine proteases convert it into various active peptides. Notably, the trypsin-like serine protease KLK5 plays a pivotal role in breaking down cathelicidin’s precursor, human cationic antimicrobial protein 18 (hCAP18; 18 kDa), to produce LL37 (36). Contemporary clinical strategies for rosacea management frequently target the TLR2–KLK5-LL37 pathway. Furthermore, drugs, such as retinoids, azelaic acid, and doxycycline, can exert regulatory effects on this pathway by downregulating KLK5 and cathelicidin expression (37–39). In the present study, one of the principal objectives was to verify that LA significantly suppressed TLR2 expression in the skin of LL-37-induced rosacea model mice. Agrahari et al. demonstrated that *in vitro* injection of KLK5 induces erythema in mice (40). To further investigate the possibility that LA exerts its effects by influencing KLK5-mediated inflammatory responses, exogenous KLK5 was locally injected into the dorsal skin of mice in the LA 60 treatment group. Notably, KLK5 induced LL-37 expression through the proteolytic cleavage of hCAP18. The results demonstrated that LA markedly reduced KLK5 protein levels in the skin tissue. However, in the LA 60 + KLK5 co-treatment group, injection of KLK5 led to exacerbated skin erythema, increased inflammatory and MC infiltration, along with the upregulation of TLR2 expression. Collectively, these findings indicate that KLK5 partially reversed the anti-inflammatory effects of LA. In summary, LA alleviates KLK5-driven inflammatory responses by suppressing KLK5 enzymatic activity, providing experimental evidence supporting its therapeutic potential in rosacea treatment.

TLRs are pattern recognition receptors that play critical roles in immunological and inflammatory responses (41). Many inflammatory processes are regulated by the ubiquitous and essential nuclear transcription factor NF-κB, which also acts as a downstream mediator of the TLR4 signaling pathway (42, 43). Pro-inflammatory cytokines such as IL-1β, IL-6, and TNF-α are upregulated when the TLR4/NF-κB signaling pathway is activated (44). Reportedly, NF-κB is a crucial transcription factor in inflammatory damage; by triggering pro-inflammatory molecules such as IL-1β, IL-6, and TNF-α, it has been shown to worsen colonic injury (45). The present study found significantly increased protein and mRNA levels of pro-inflammatory cytokines IL-1β, IL-6, and TNF-α in the dorsal skin and sera of model group mice. LA treatment effectively downregulated these cytokines, suggesting that LA ameliorates rosacea-like inflammation by suppressing the TLR4/NF-κB signaling pathway. Notably, this study provides only a cursory investigation and deduction of the mechanism of LA in rosacea treatment. Further molecular biology research is warranted for comprehensive elucidation of the precise regulatory mechanisms of LA within the TLR4/NF-κB signaling to validate the therapeutic relevance of LA in rosacea.

Metabolomics, a powerful systems biology approach, enables the evaluation of the quantities of all metabolites found in a biological system and establishes the relationship between metabolites and physiological and pathological abnormalities (46). Herein, multivariate statistical methods were used in untargeted metabolomics to identify changes in LL-37-associated metabolites in rosacea. OPLS-DA demonstrated clear separation between the metabolic profiles of the model, control, and LA 60 groups, with the difference in the metabolic profiles of the model and control groups being significant. These findings further substantiate the ability of LA to treat LL-37-induced rosacea in mice. This untargeted metabolomics analysis identified 30 new potential biomarkers reflecting the metabolic dynamics of LL-37-induced rosacea following LA treatment. Notably, phenylalanine metabolites were the predominant metabolites influencing LL-37-induced rosacea. They may represent key metabolic pathways involved in modulating the pathological progression and underlying molecular mechanisms of rosacea. Phenylalanine metabolism abnormalities are frequently associated with dysregulated reactive oxygen species(ROS)production (47). The NOX2/ROS/NF-κB signaling pathway may mediate the inflammatory response and vascular alterations observed in rosacea (48). 3-(2-Hydroxyphenyl) propanoic acid is a known product of phenylalanine metabolism via microbial or alternative pathways (49). Similarly, phenyllactic acid is another metabolite derived from phenylalanine and lactate metabolism (50). In the present study, the concentrations of both phenyllactic acid and 3-(2-hydroxyphenyl)propanoic acid decreased in the LL-37-induced group, albeit LA treatment increased their contents. These results suggest that LA may exerts its anti-rosacea effects by modulating phenylalanine metabolism. The identified biomarkers, both in LL-37-induced murine rosacea and after LA intervention, provide valuable insight into delineating the pathways and mechanisms associated with rosacea pathogenesis.

## 5 Conclusion

**Figure 9:**
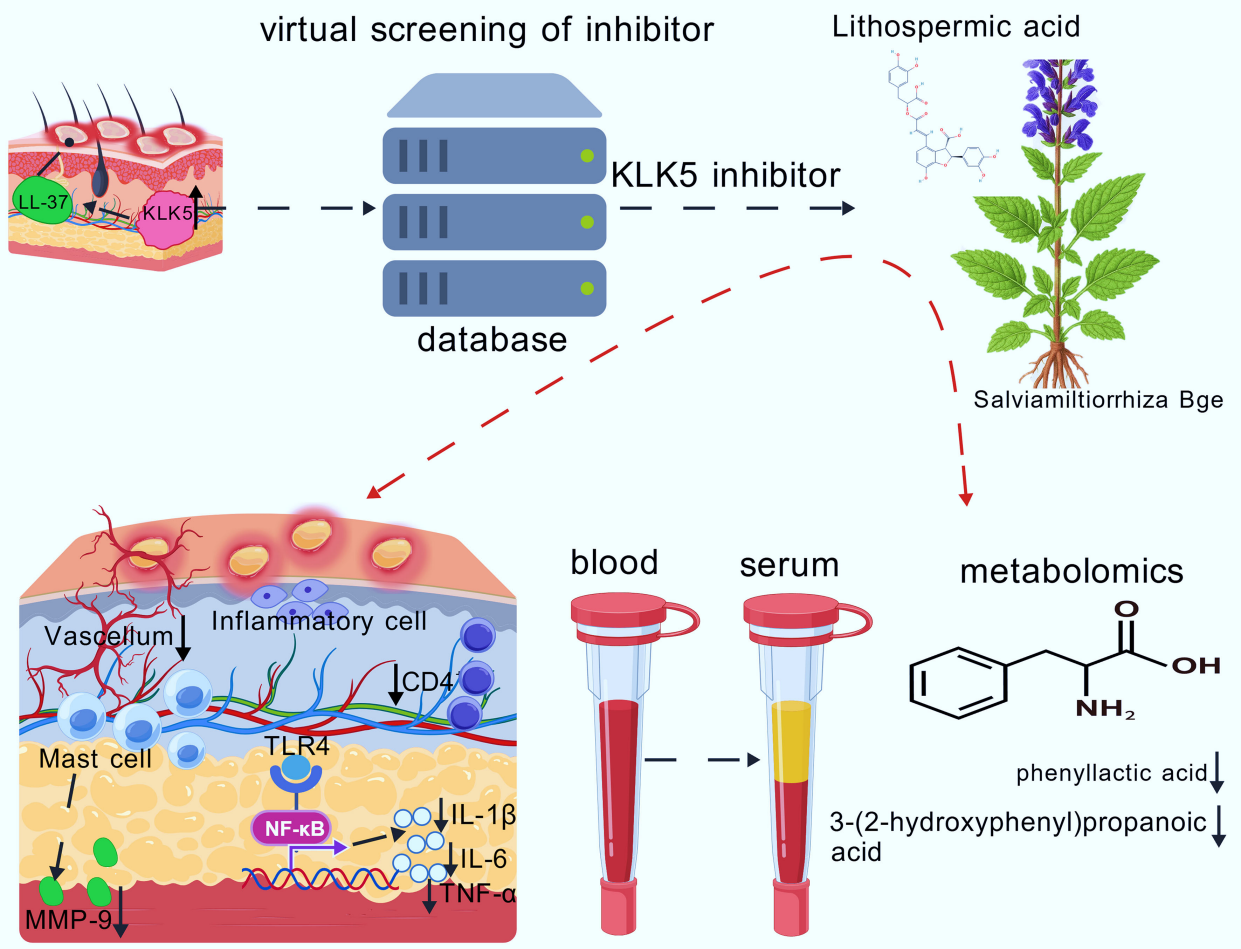
Schematic representation of the basic mechanism of rosacea alleviation by the LA of KLK5 inhibitor.

In conclusion, this study identified LA as a potent KLK5 inhibitor and a promising therapeutic candidate for rosacea through VS. Intraperitoneal administration of LA effectively alleviated LL-37-induced rosacea-like symptoms, including erythema, elevated inflammatory cytokines, MC infiltration, cutaneous angiogenesis, and immune cell infiltration. Evidence from animal experiments and serum metabolomics indicated that LA mitigates rosacea by modulating phenylalanine metabolism and inhibiting activation of the TLR4/NF-κB signaling pathway. Altogether, this study provides the first mechanistic insight into the potential therapeutic mechanism of LA in rosacea, supporting its further development as a natural and effective treatment modality (**Figure 9**).

## Data Availability

The datasets presented in this study can be found in online repositories. The names of the repository/repositories and accession number(s) can be found below: MTBLS13282(Metabolights; https://www.ebi.ac.uk/metabolights/reviewerfdec28b3-adf2-4be4-a0e6-61a1a1ffc73e).
