# Supplementary figures and images for: Lithospermic acid, a novel KLK5 inhibitor, ameliorates rosacea by suppressing the TLR4/NF-κB signaling pathway and rectifying phenylalanine metabolism (part 1 of 2)
Source: Front Immunol. 2026 Jan 26;17:1734997. doi: 10.3389/fimmu.2026.1734997 (PMC12883415; doi:10.3389/fimmu.2026.1734997)

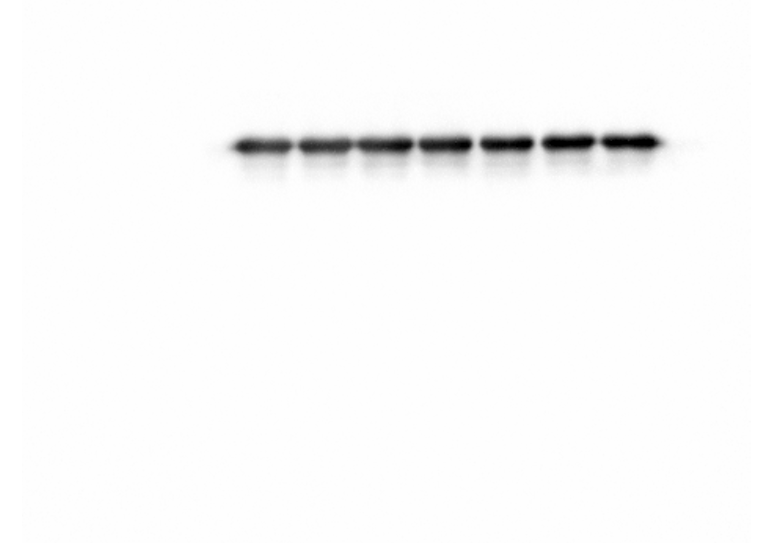

Supplement: Supplementary file 1 [file DataSheet1.zip › western blot images/GAPDH/gapdh-1.tif]

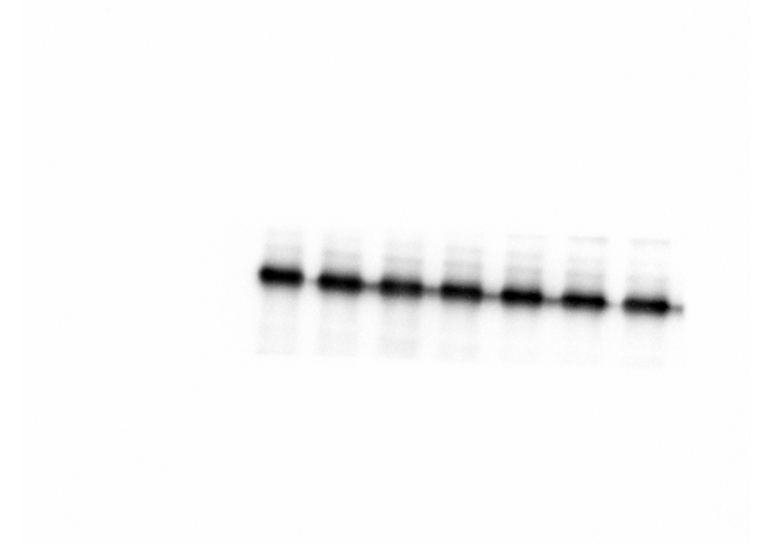

Supplement: Supplementary file 1 [file DataSheet1.zip › western blot images/GAPDH/gapdh-2-5.8.tif]

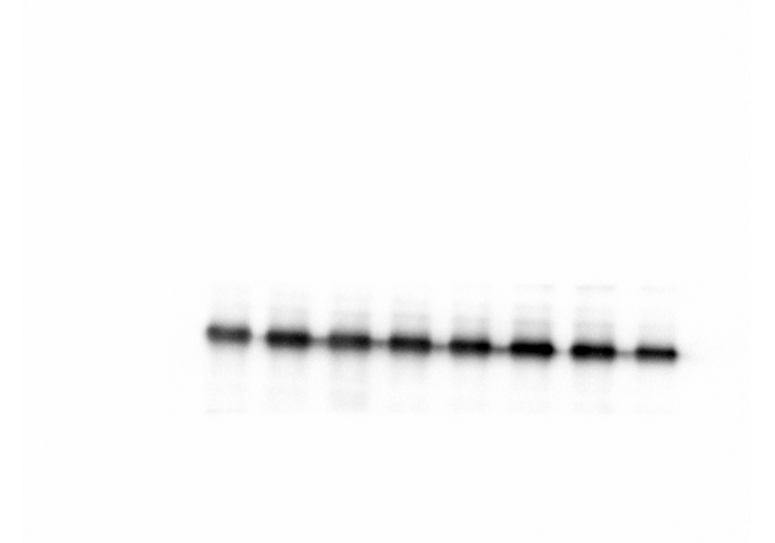

Supplement: Supplementary file 1 [file DataSheet1.zip › western blot images/GAPDH/gapdh-2.tif]

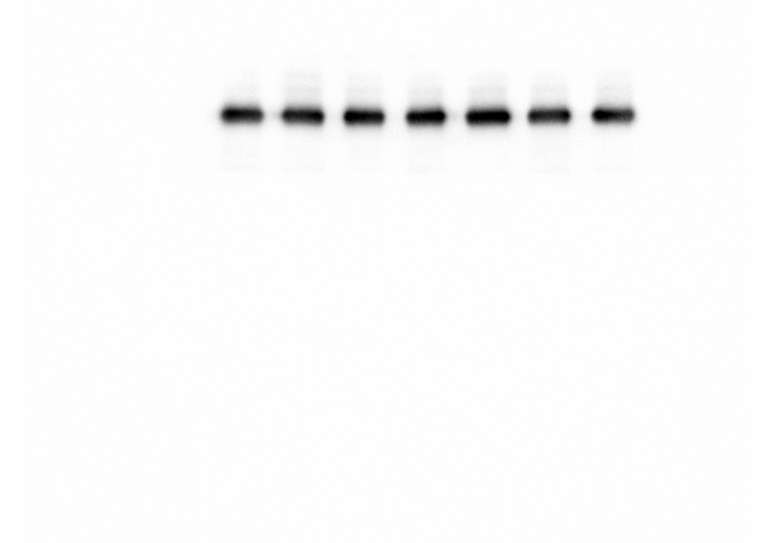

Supplement: Supplementary file 1 [file DataSheet1.zip › western blot images/GAPDH/gapdh-3-5.10.tif]

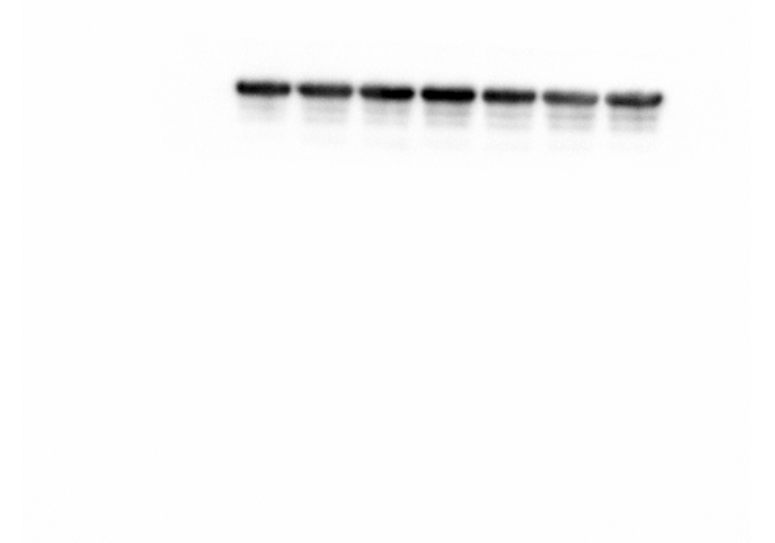

Supplement: Supplementary file 1 [file DataSheet1.zip › western blot images/GAPDH/gapdh-3.tif]

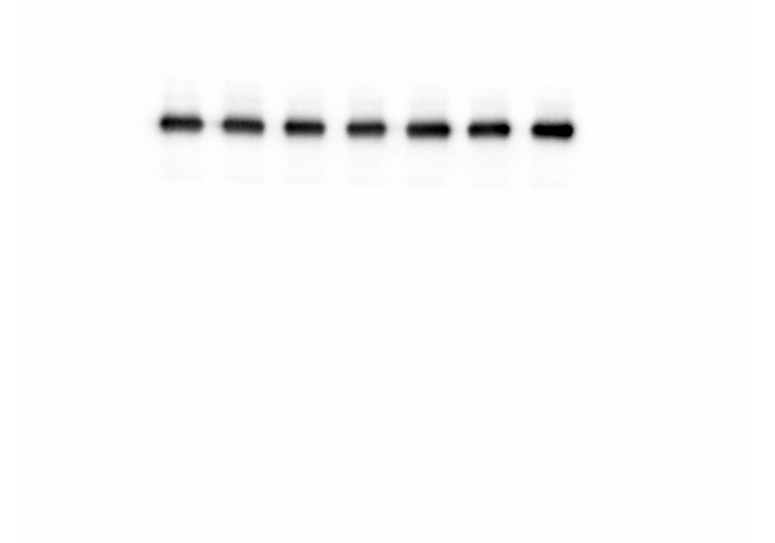

Supplement: Supplementary file 1 [file DataSheet1.zip › western blot images/GAPDH/gapdh-4-5.10.tif]

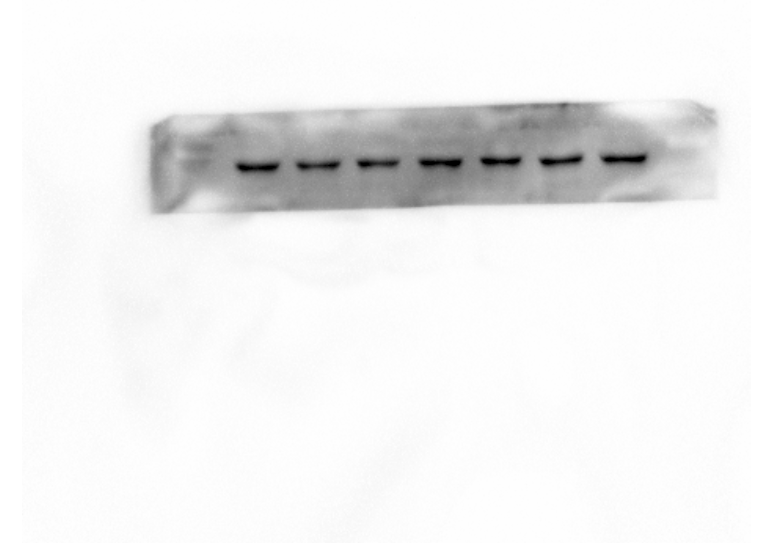

Supplement: Supplementary file 1 [file DataSheet1.zip › western blot images/P65/p65-1.tif]

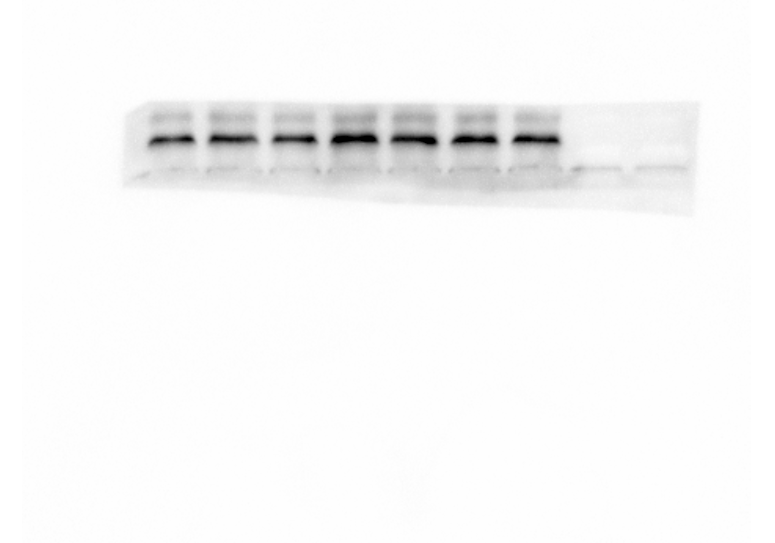

Supplement: Supplementary file 1 [file DataSheet1.zip › western blot images/P65/p65-2.tif]

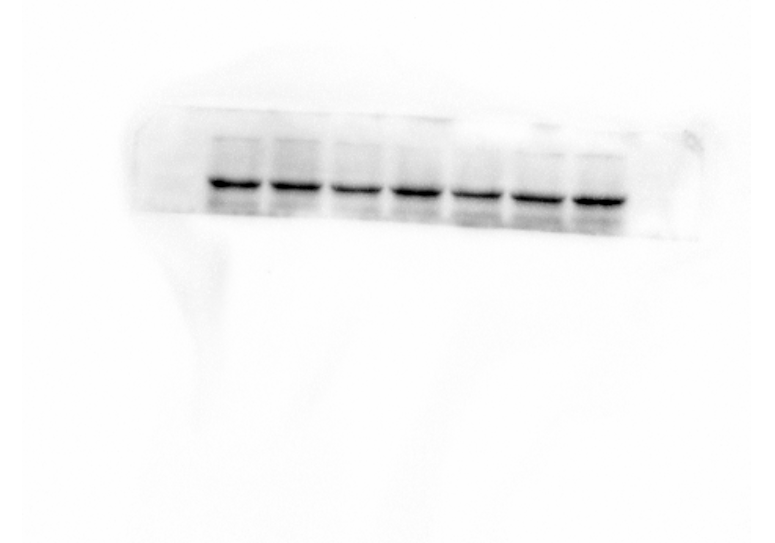

Supplement: Supplementary file 1 [file DataSheet1.zip › western blot images/P65/p65-3.tif]

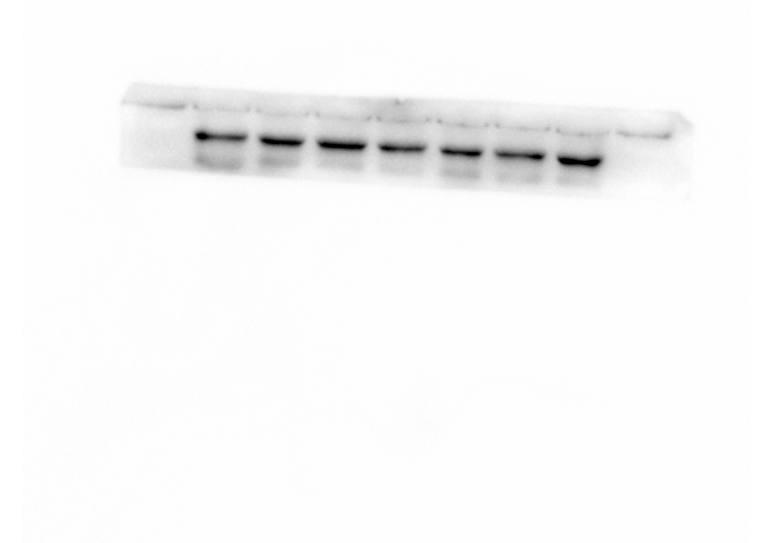

Supplement: Supplementary file 1 [file DataSheet1.zip › western blot images/P65/p65-4.tif]

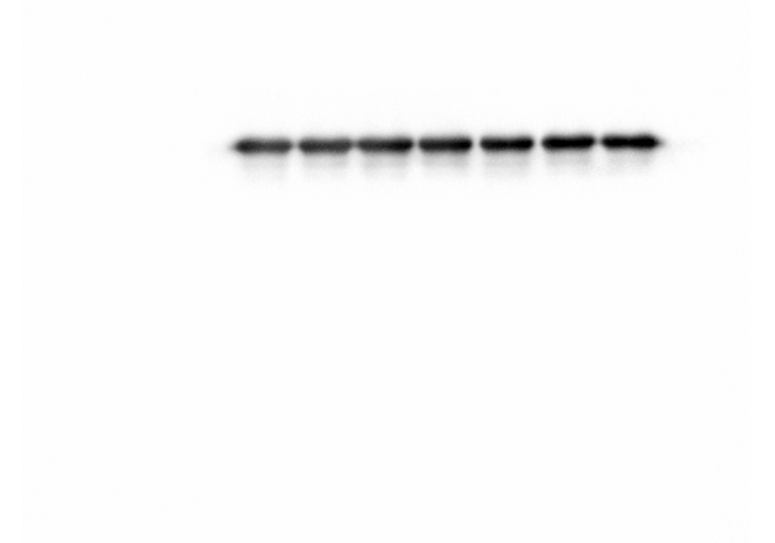

Supplement: Supplementary file 1 [file DataSheet1.zip › western blot images/PP65/gapdh-1作图.tiff]

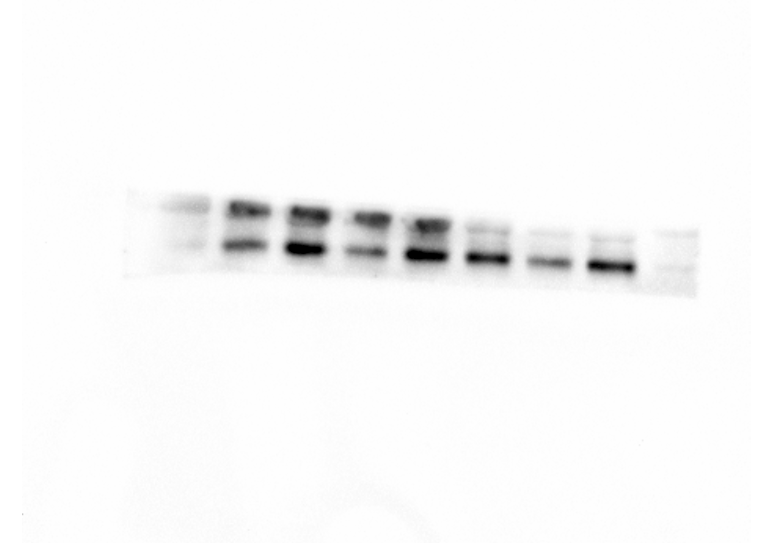

Supplement: Supplementary file 1 [file DataSheet1.zip › western blot images/PP65/pp65-1.tif]

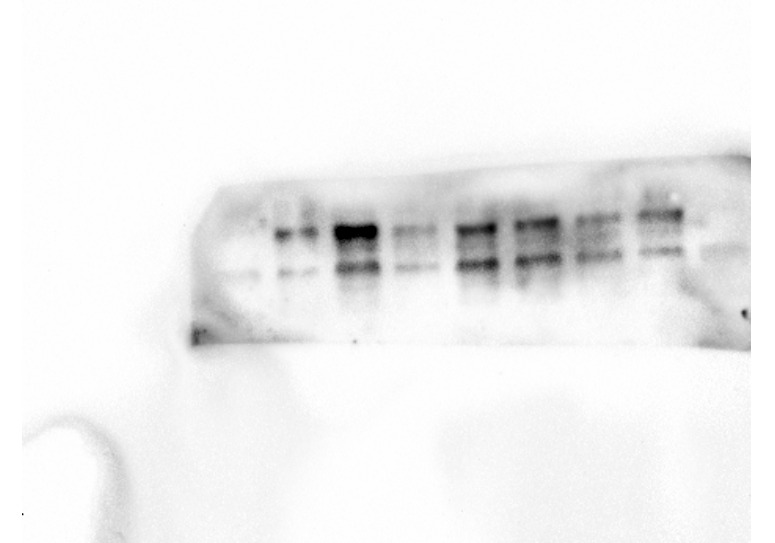

Supplement: Supplementary file 1 [file DataSheet1.zip › western blot images/PP65/pp65-2.tif]

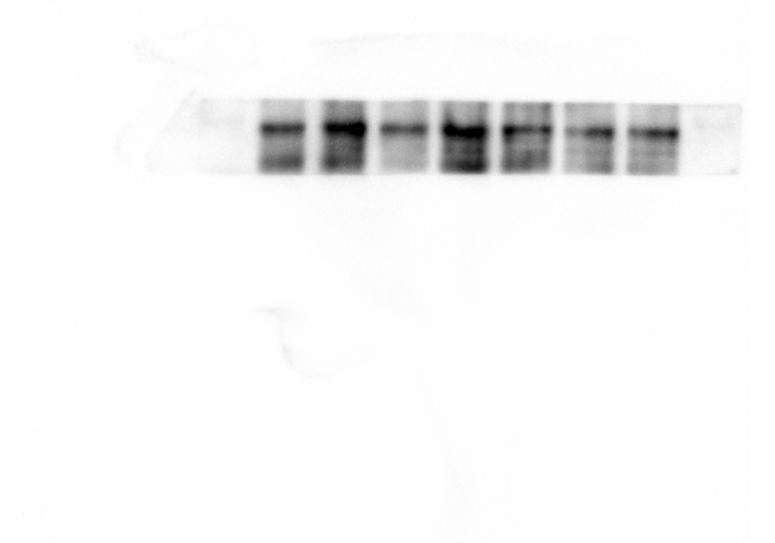

Supplement: Supplementary file 1 [file DataSheet1.zip › western blot images/PP65/pp65-3.tif]

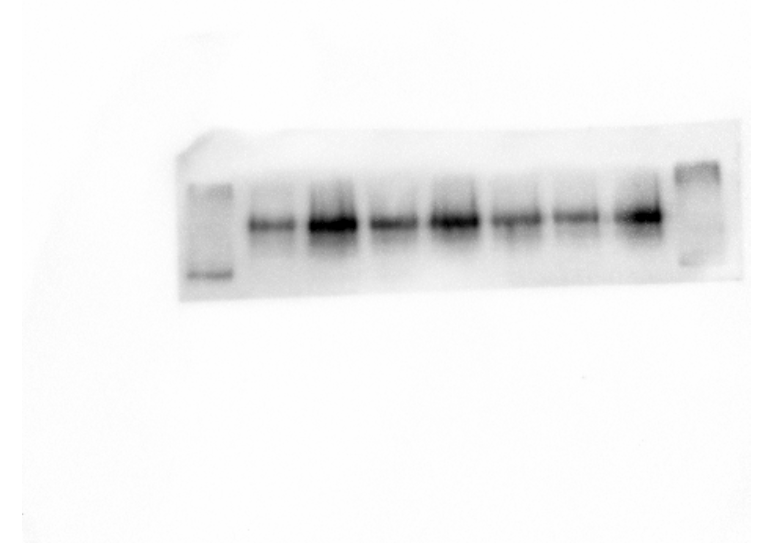

Supplement: Supplementary file 1 [file DataSheet1.zip › western blot images/TLR4/TLR4-1.tiff]

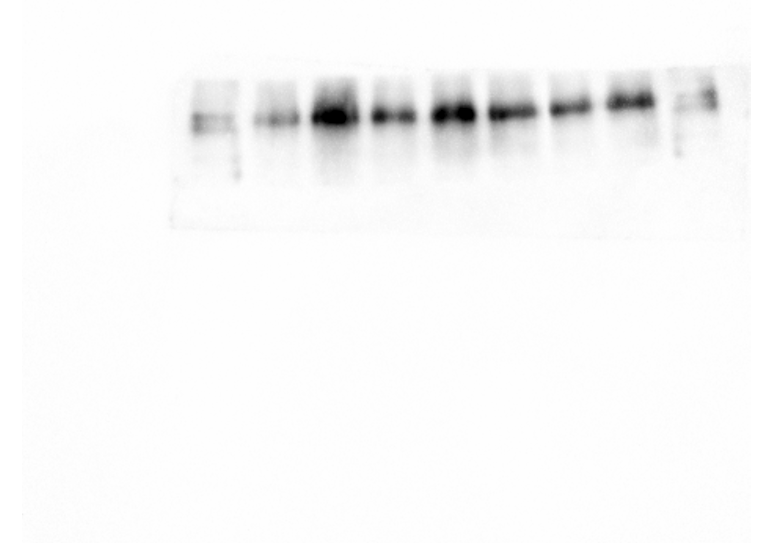

Supplement: Supplementary file 1 [file DataSheet1.zip › western blot images/TLR4/TLR4-2.tif]

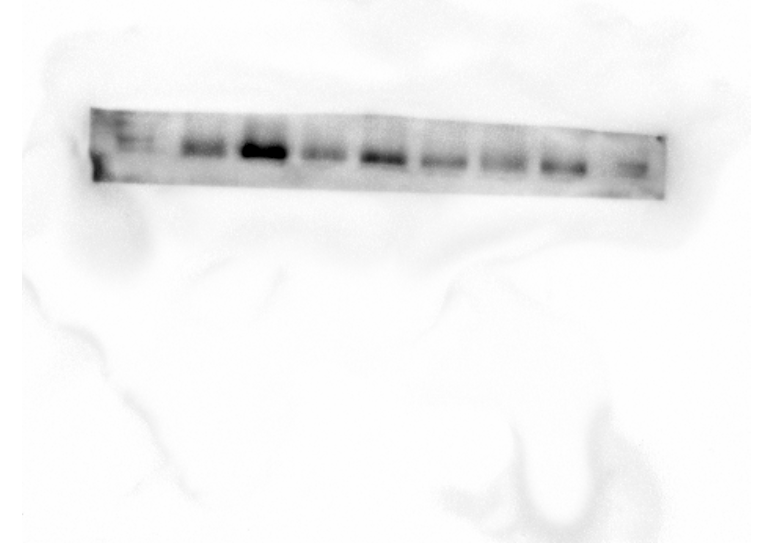

Supplement: Supplementary file 1 [file DataSheet1.zip › western blot images/TLR4/TLR4-3.tif]

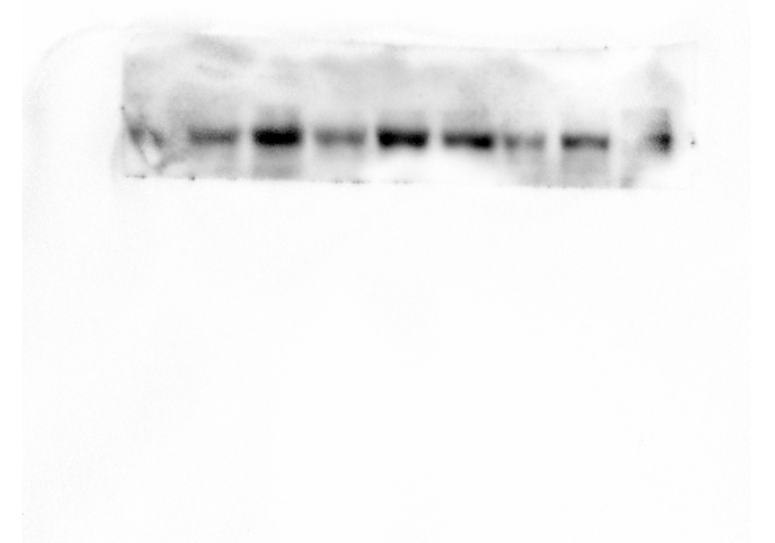

Supplement: Supplementary file 1 [file DataSheet1.zip › western blot images/TLR4/TLR4-4.tif]

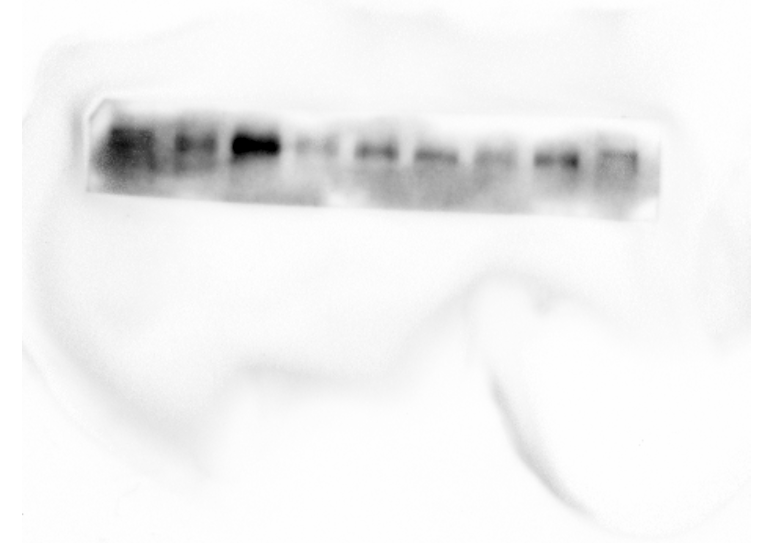

Supplement: Supplementary file 1 [file DataSheet1.zip › western blot images/TLR4/toll-2-1-7.7.tif]

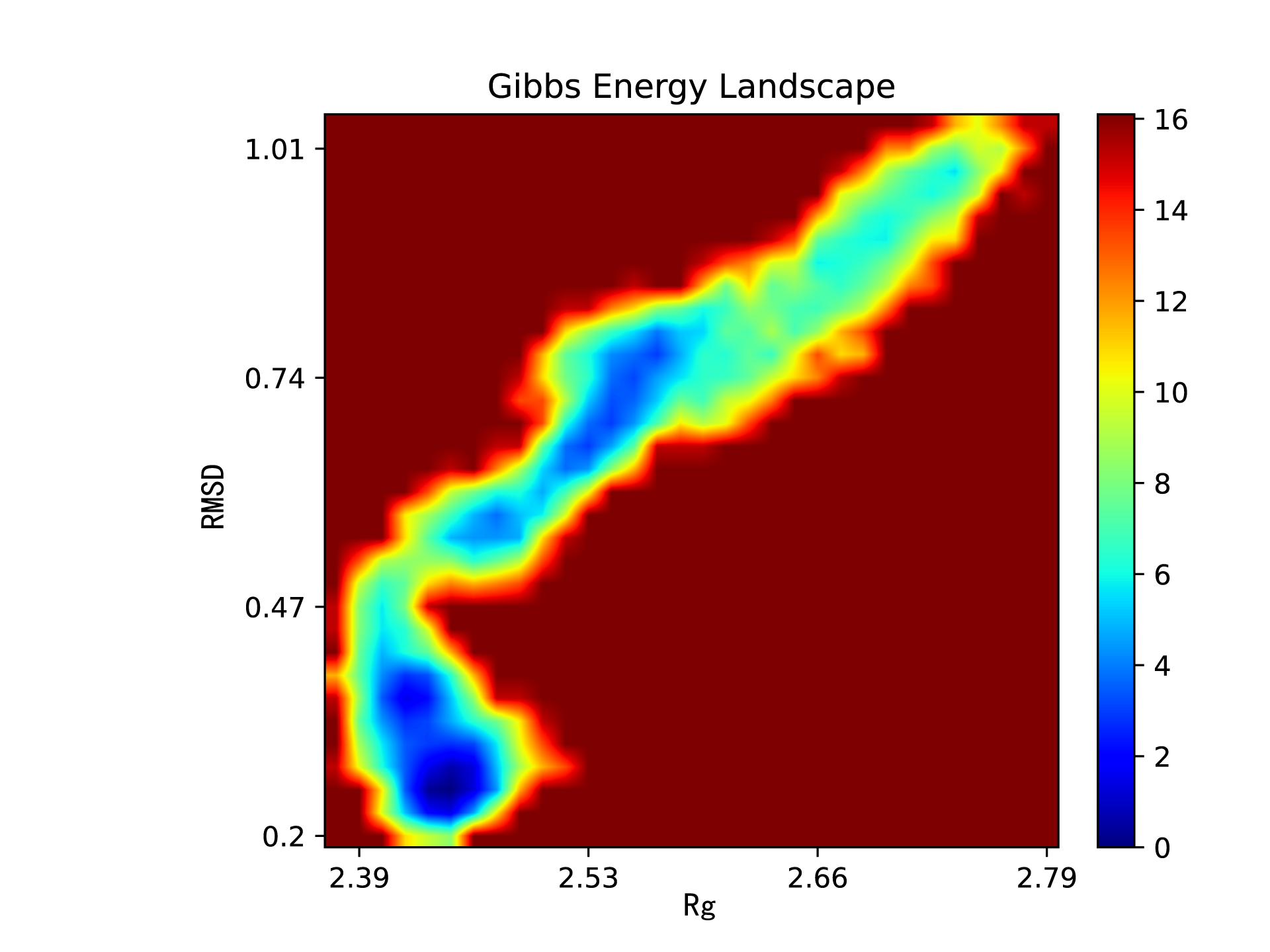

Supplement: Supplementary file 2 [file DataSheet2.zip › LA-Molecular dynamics simulation/2D.tif]

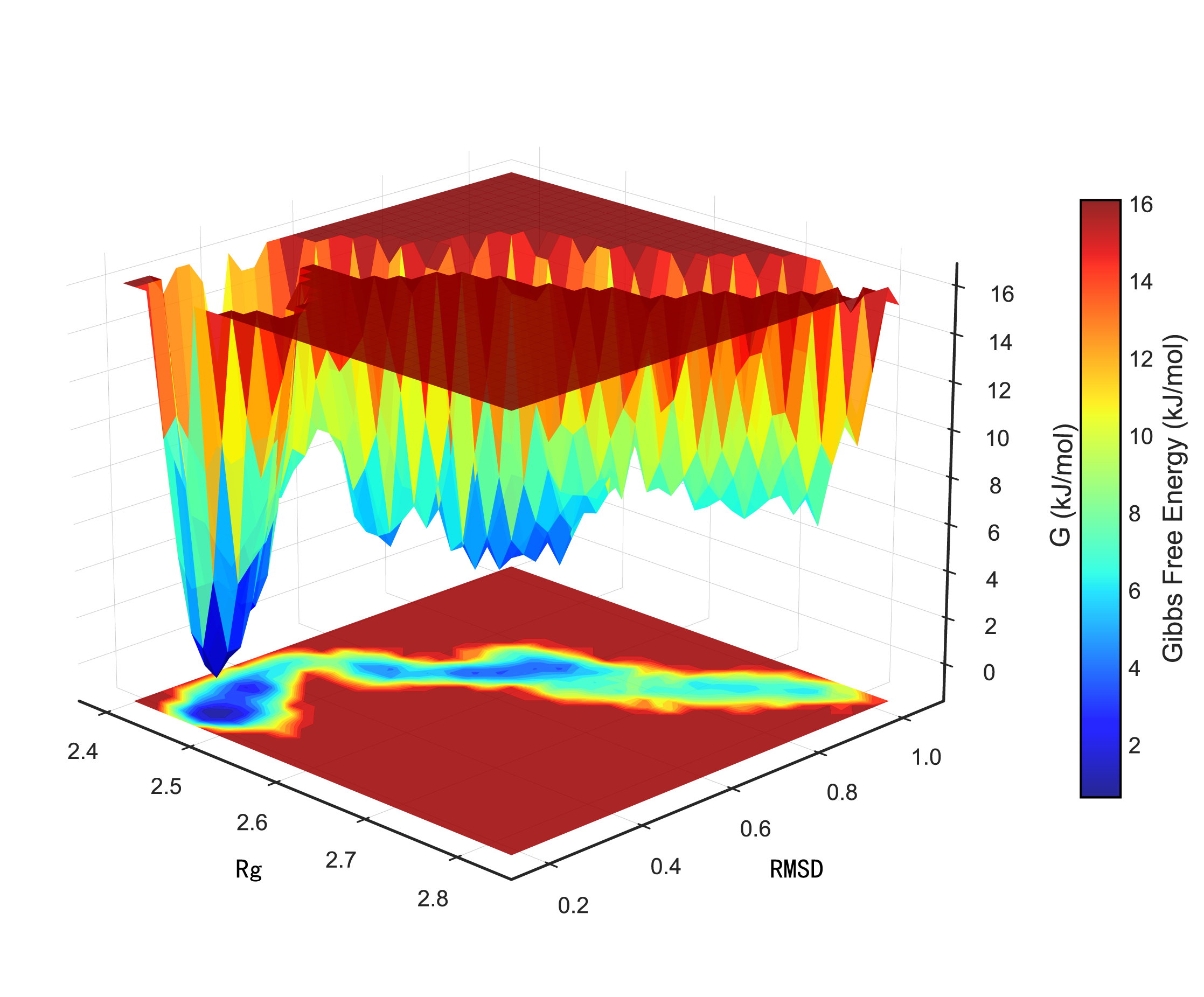

Supplement: Supplementary file 2 [file DataSheet2.zip › LA-Molecular dynamics simulation/3D.tif]

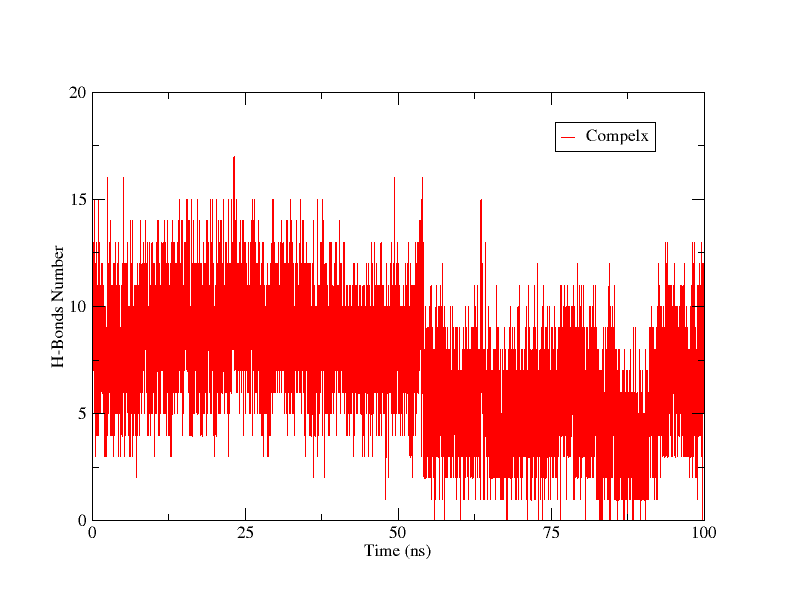

Supplement: Supplementary file 2 [file DataSheet2.zip › LA-Molecular dynamics simulation/H-B.tif]

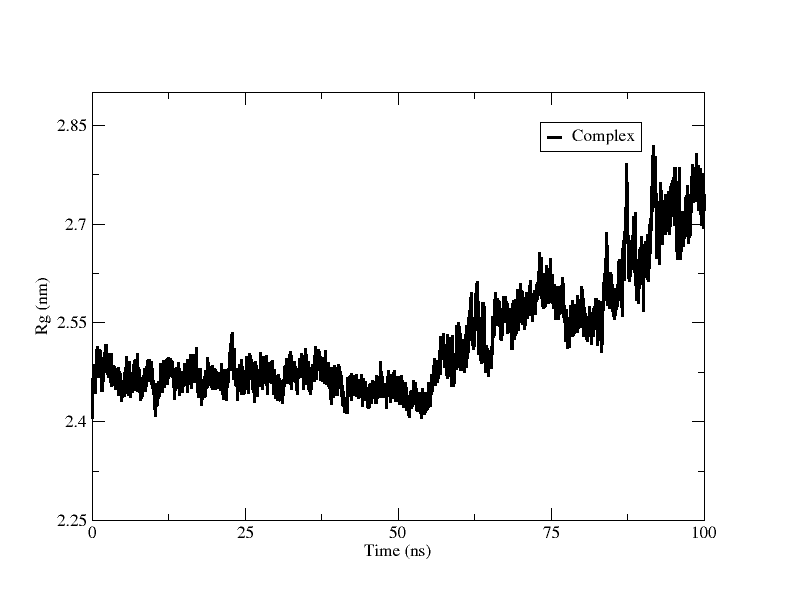

Supplement: Supplementary file 2 [file DataSheet2.zip › LA-Molecular dynamics simulation/Rg.tif]

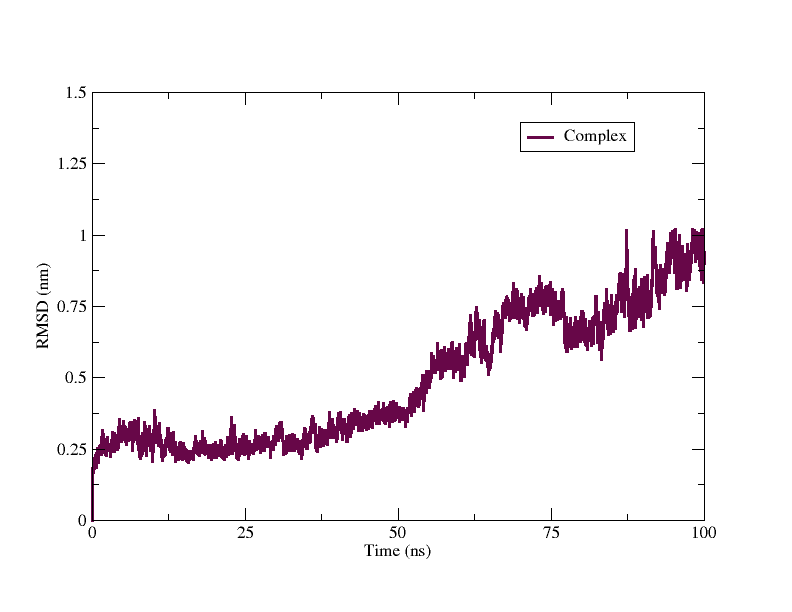

Supplement: Supplementary file 2 [file DataSheet2.zip › LA-Molecular dynamics simulation/RMSD.tif]

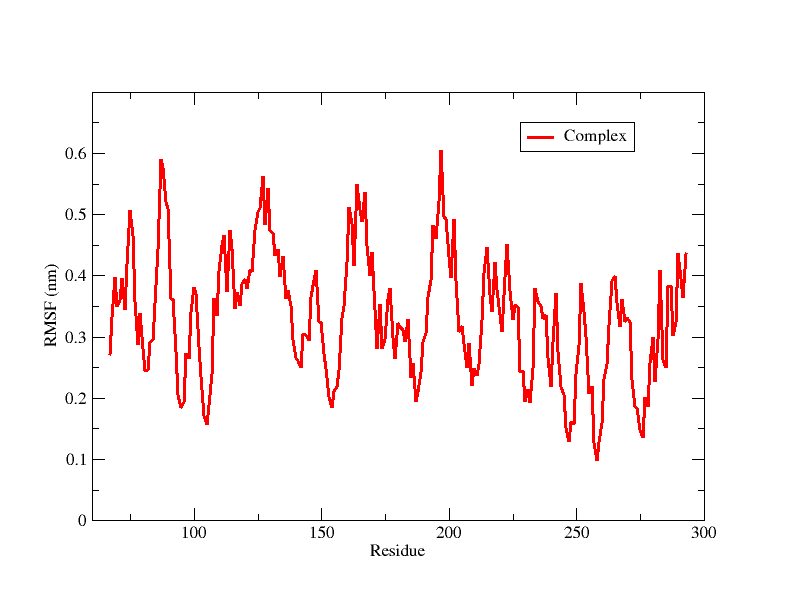

Supplement: Supplementary file 2 [file DataSheet2.zip › LA-Molecular dynamics simulation/RMSF.tif]

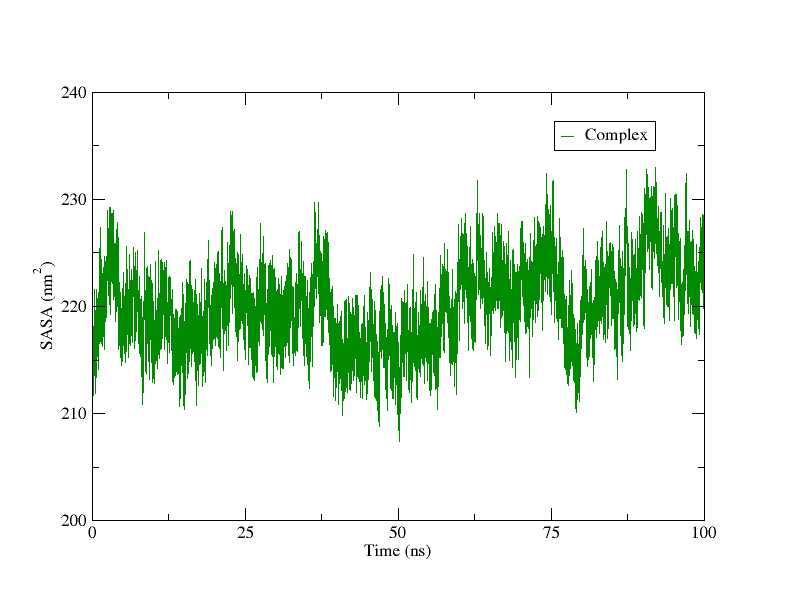

Supplement: Supplementary file 2 [file DataSheet2.zip › LA-Molecular dynamics simulation/SASA.tif]

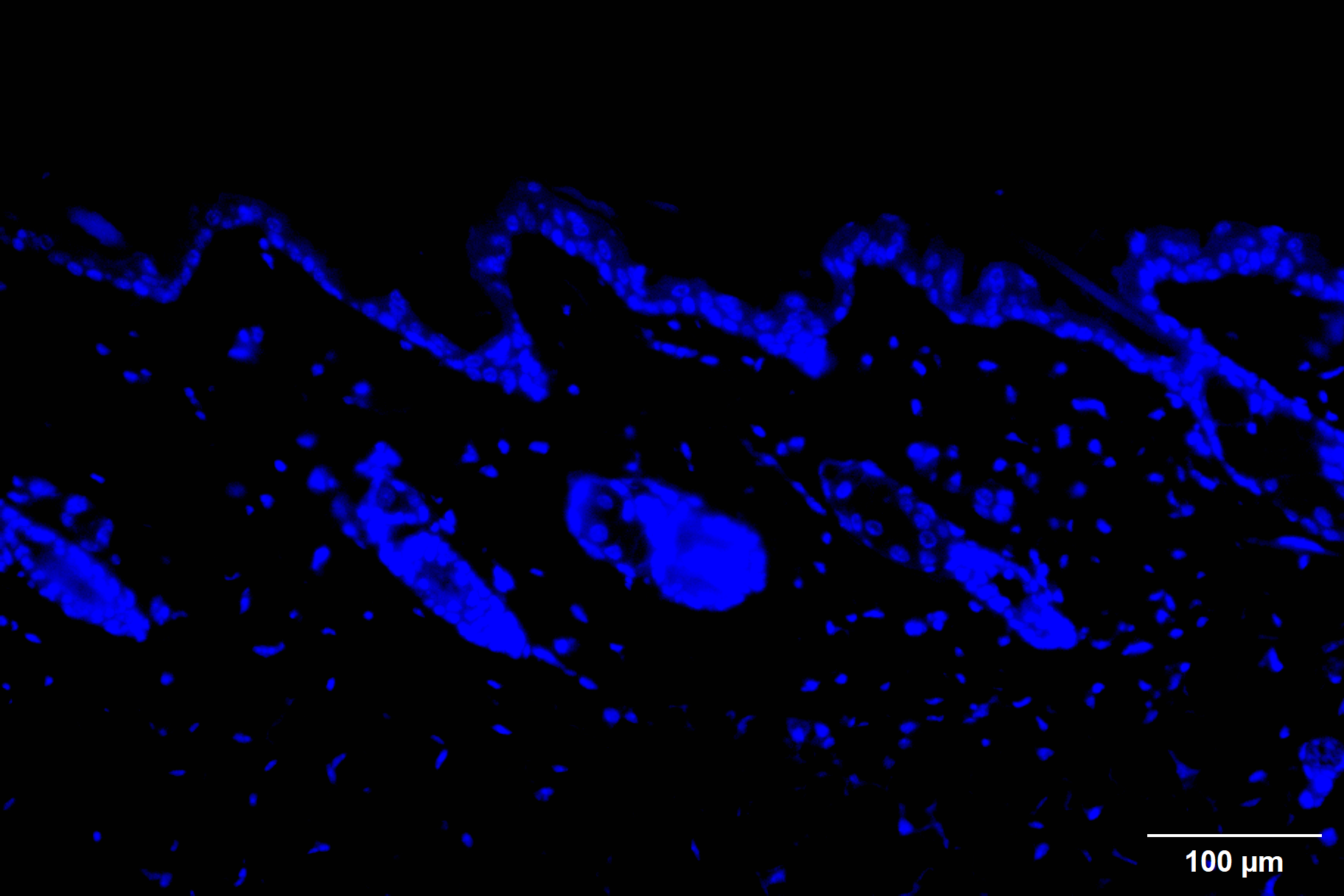

Supplement: Supplementary file 4 [file DataSheet4.zip › LA-Immunofluorescence staining image-Figure 4C/Figure 4C/1-1.tif]

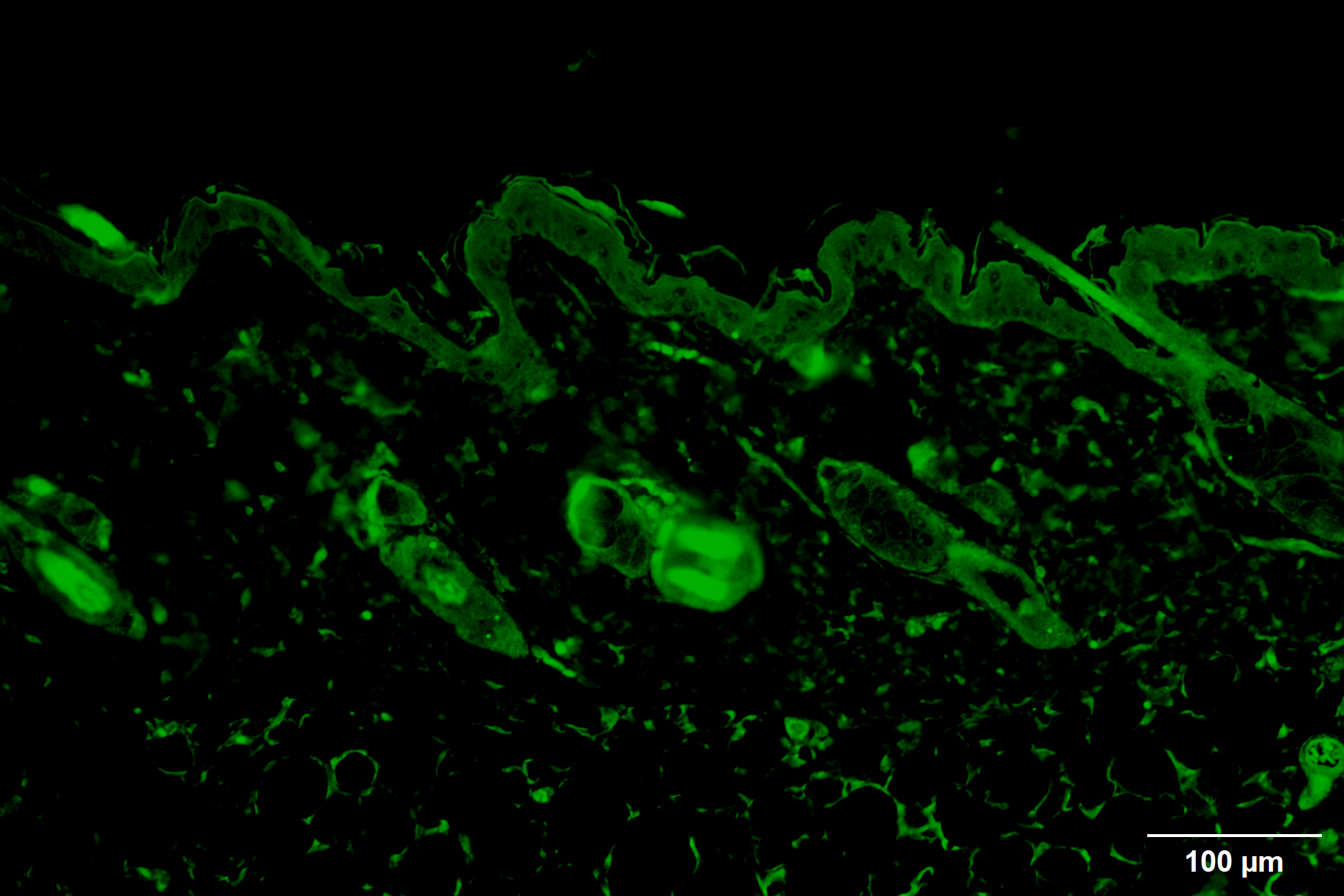

Supplement: Supplementary file 4 [file DataSheet4.zip › LA-Immunofluorescence staining image-Figure 4C/Figure 4C/1-2.tif]

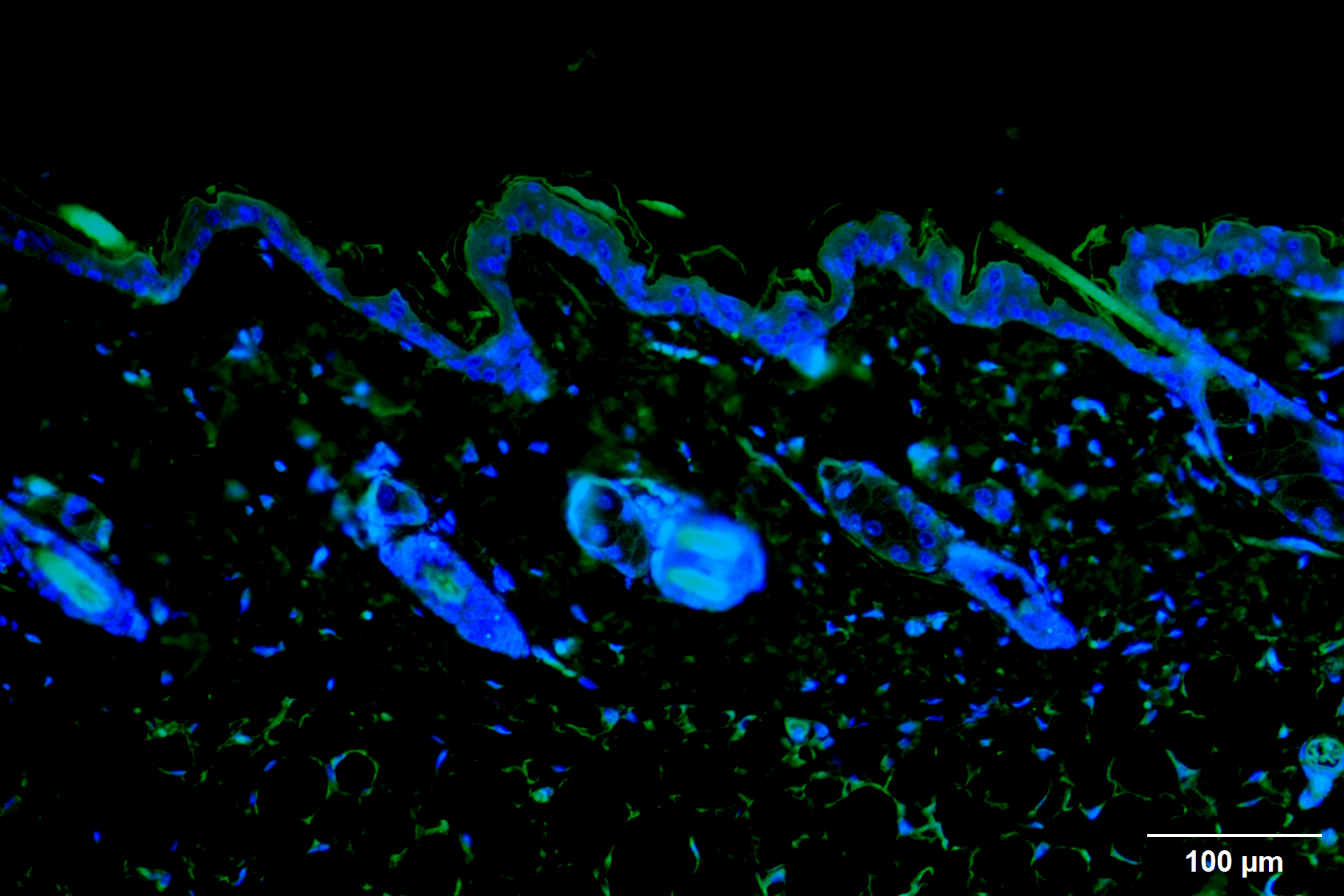

Supplement: Supplementary file 4 [file DataSheet4.zip › LA-Immunofluorescence staining image-Figure 4C/Figure 4C/1-3.tif]

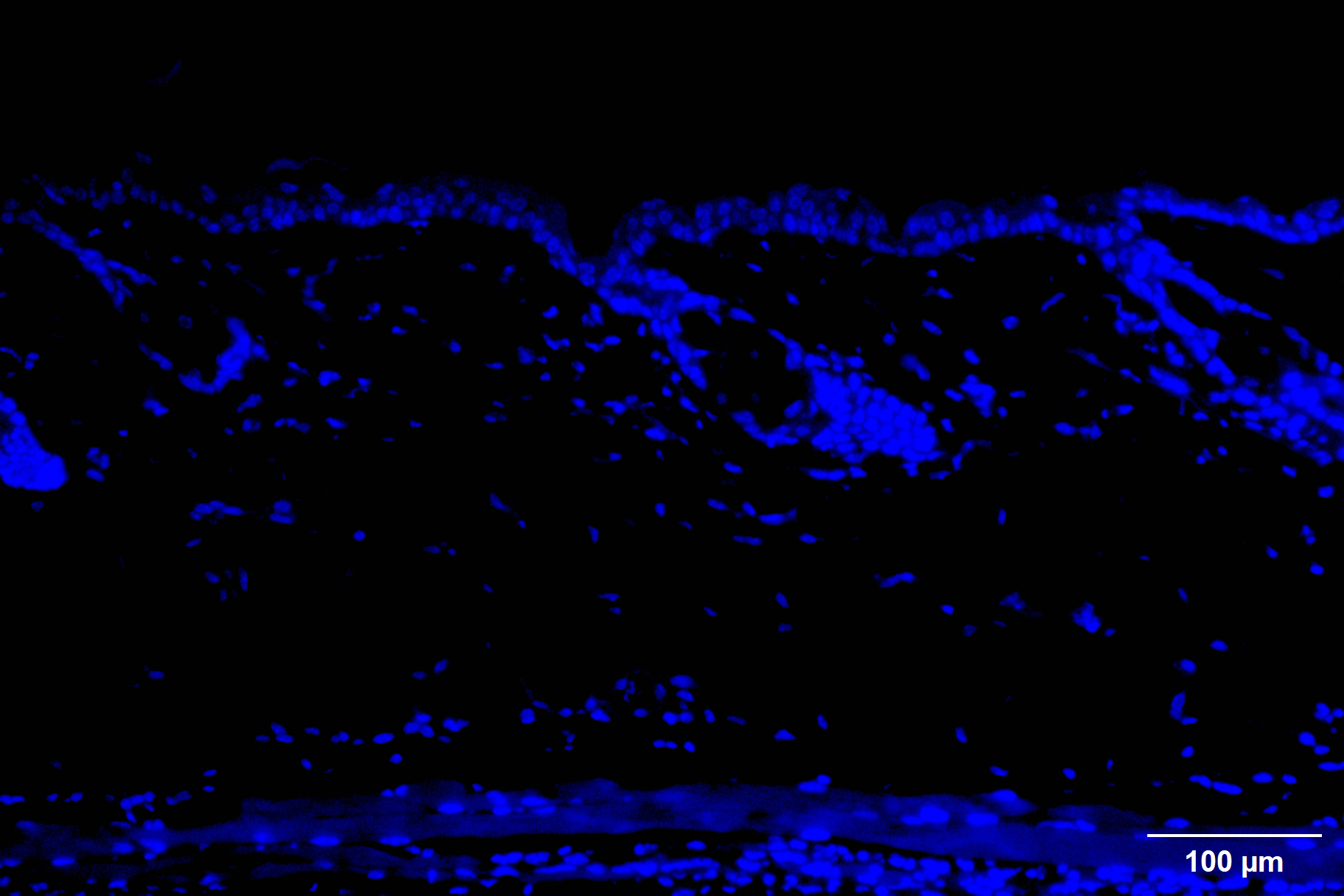

Supplement: Supplementary file 4 [file DataSheet4.zip › LA-Immunofluorescence staining image-Figure 4C/Figure 4C/2-1.tif]

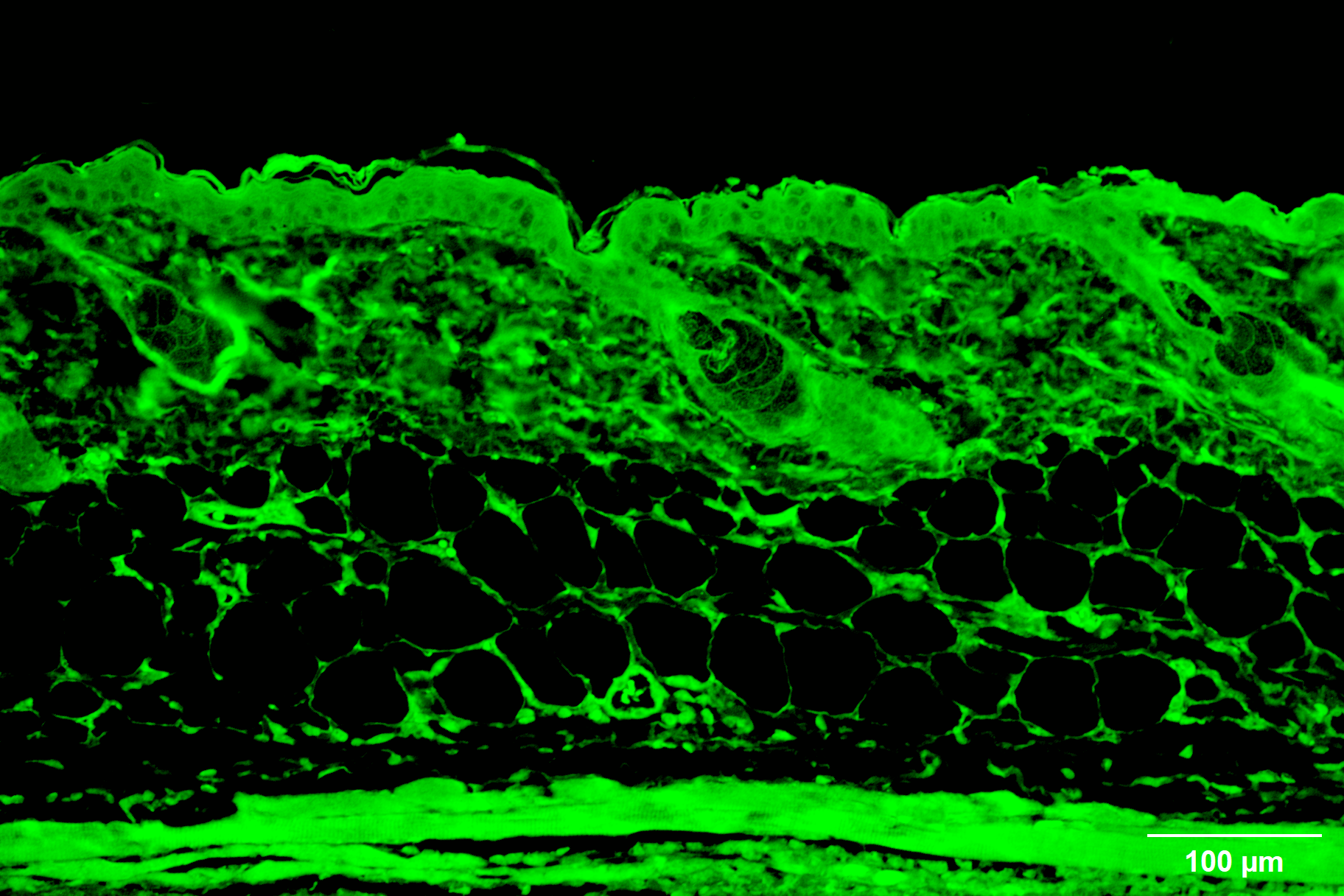

Supplement: Supplementary file 4 [file DataSheet4.zip › LA-Immunofluorescence staining image-Figure 4C/Figure 4C/2-2.tif]

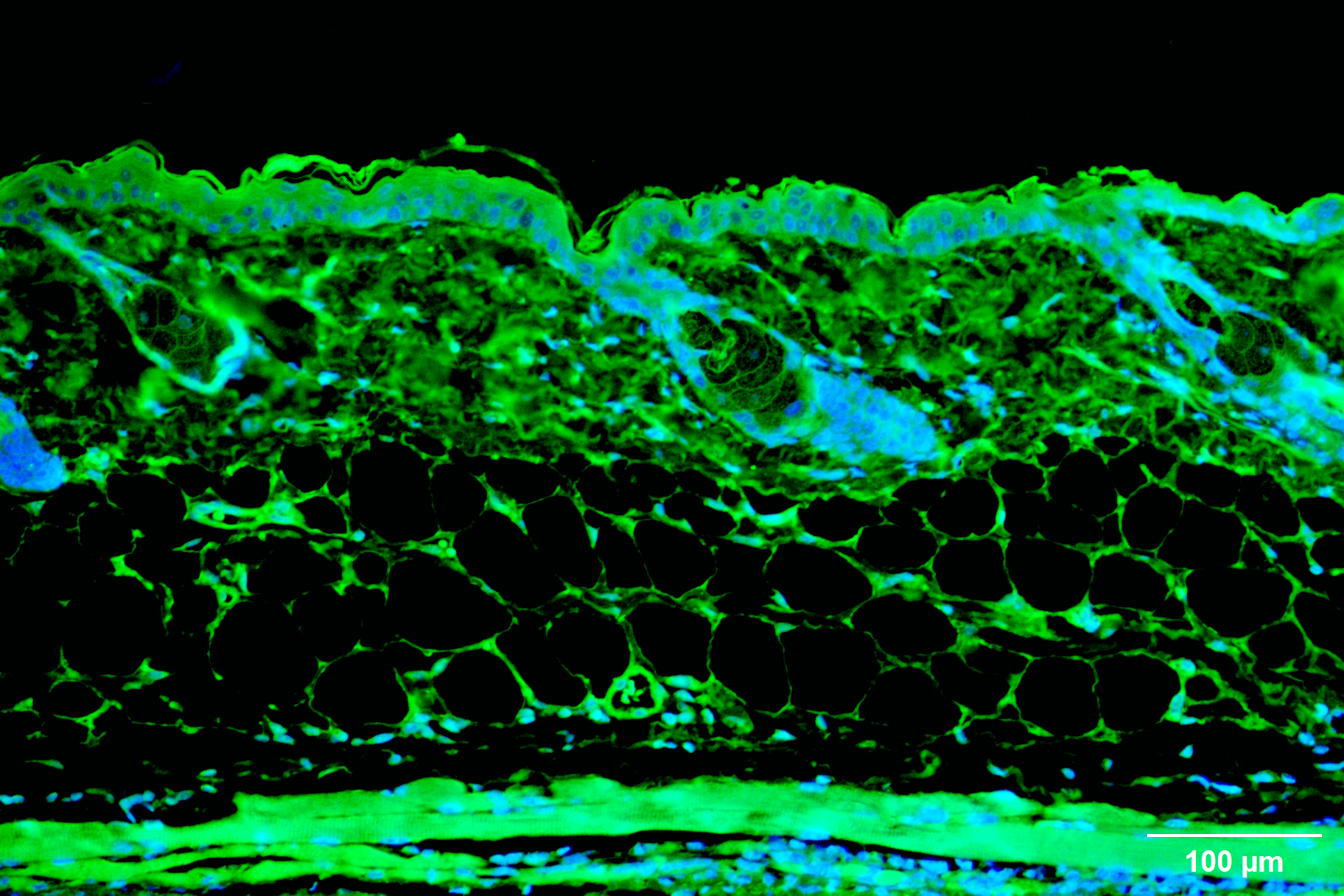

Supplement: Supplementary file 4 [file DataSheet4.zip › LA-Immunofluorescence staining image-Figure 4C/Figure 4C/2-3.tif]

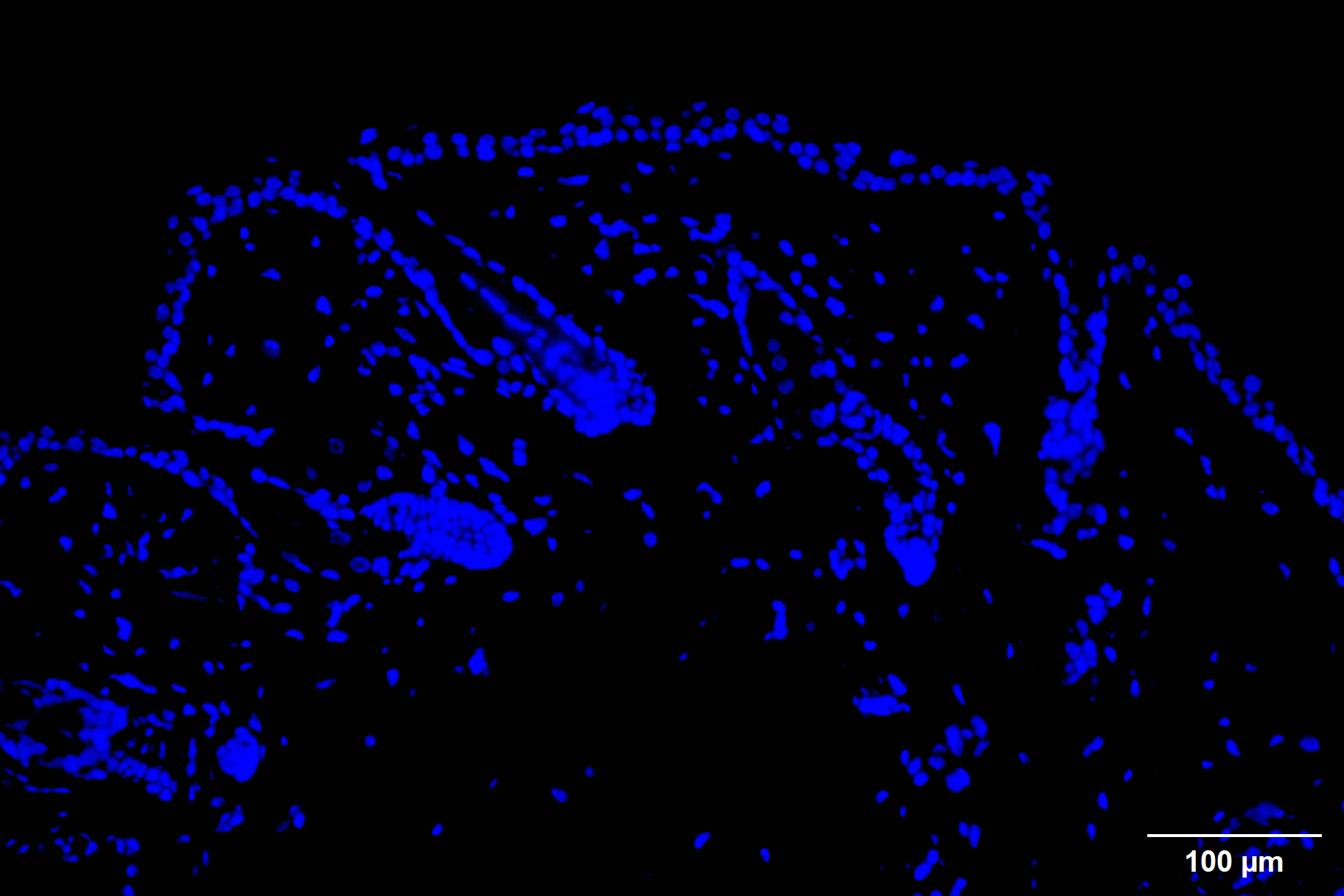

Supplement: Supplementary file 4 [file DataSheet4.zip › LA-Immunofluorescence staining image-Figure 4C/Figure 4C/3-1.tif]

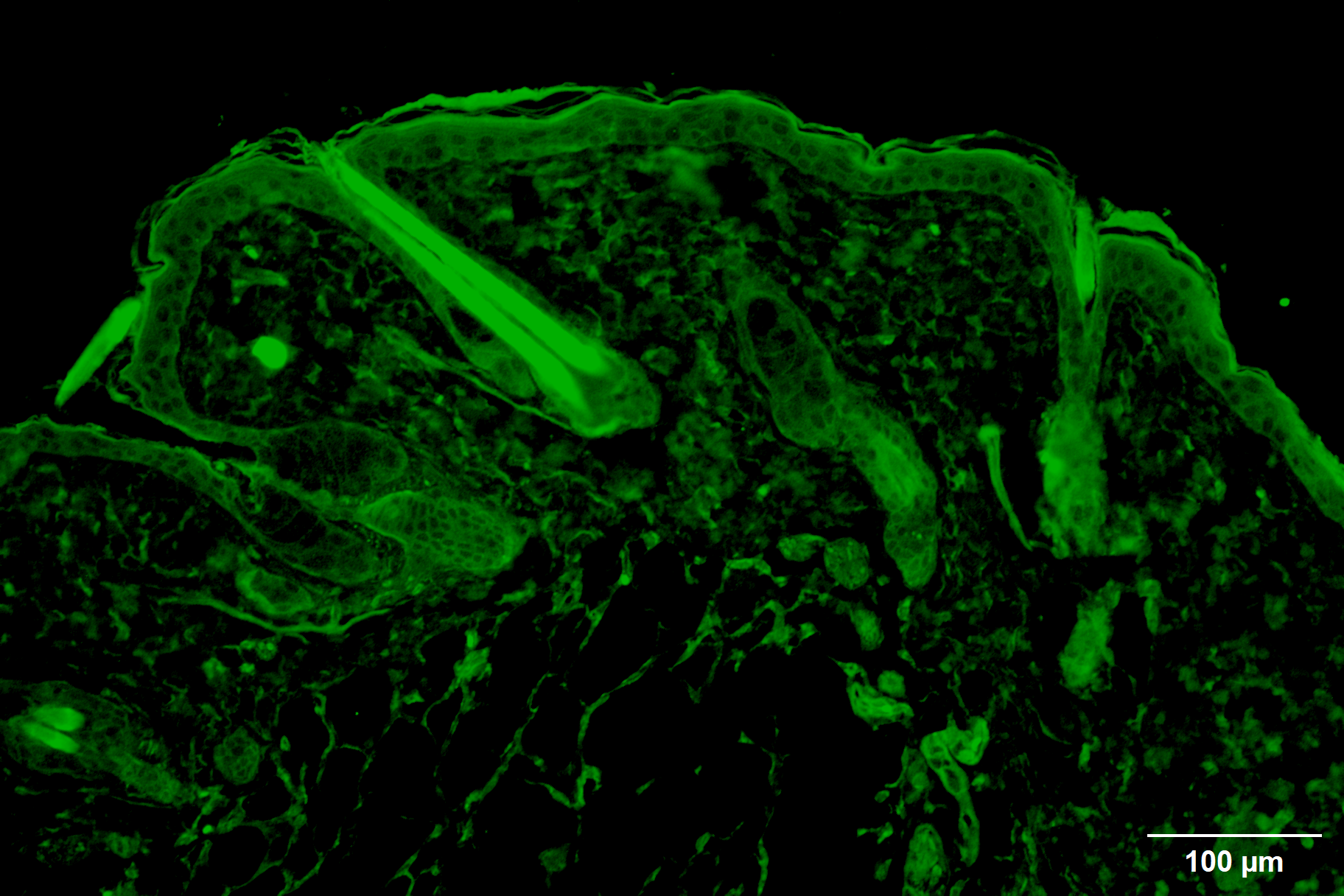

Supplement: Supplementary file 4 [file DataSheet4.zip › LA-Immunofluorescence staining image-Figure 4C/Figure 4C/3-2.tif]

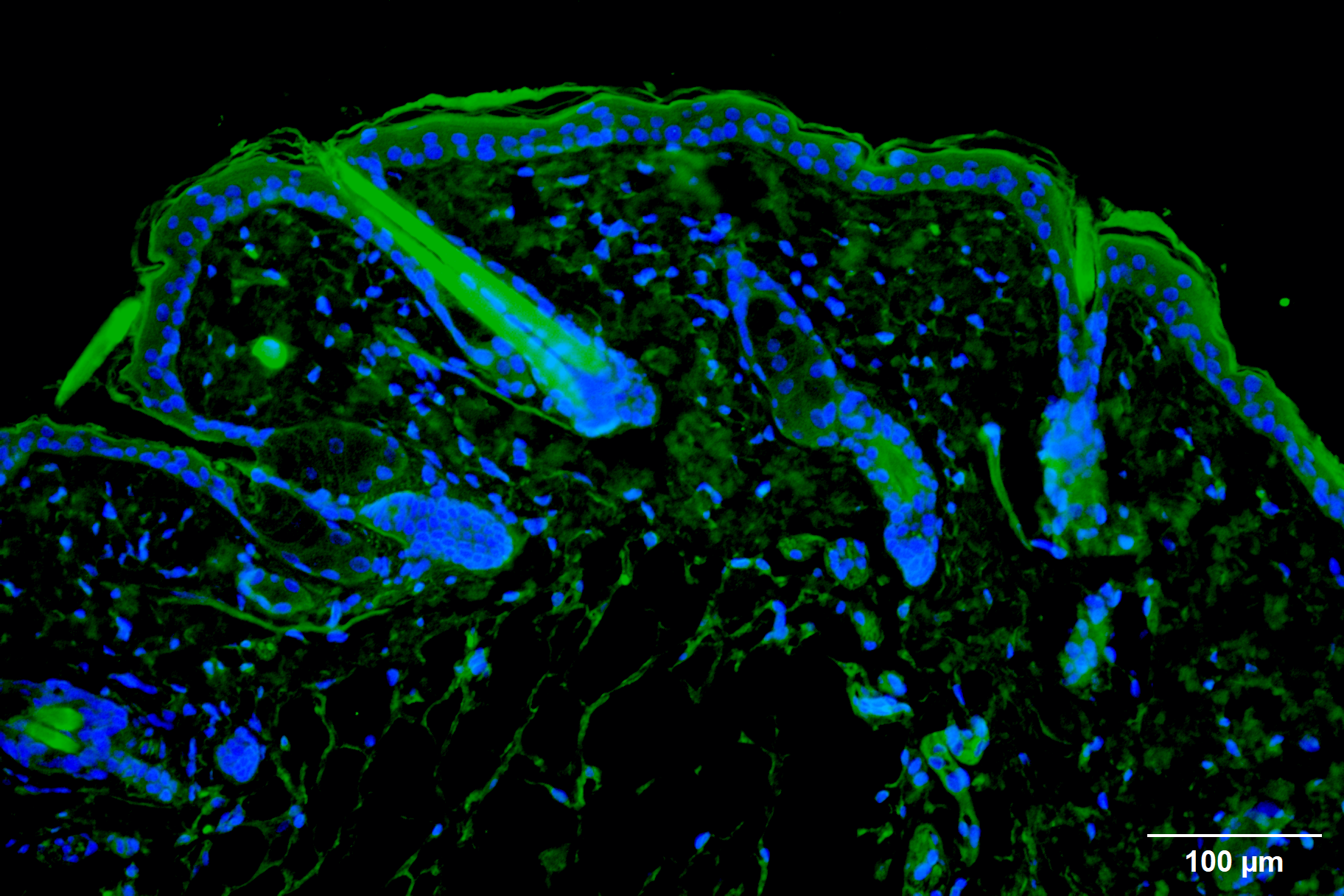

Supplement: Supplementary file 4 [file DataSheet4.zip › LA-Immunofluorescence staining image-Figure 4C/Figure 4C/3-3.tif]

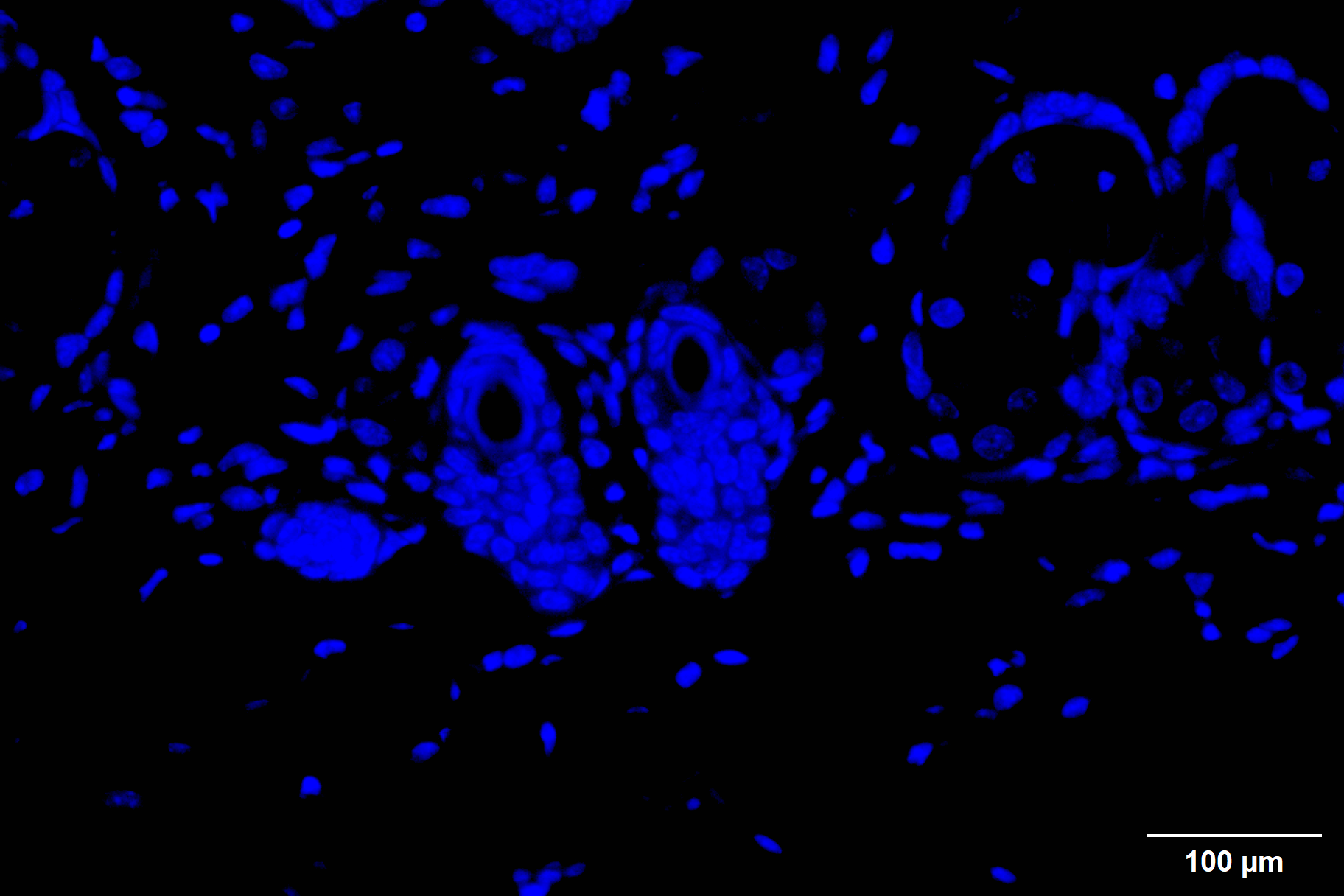

Supplement: Supplementary file 4 [file DataSheet4.zip › LA-Immunofluorescence staining image-Figure 4C/Figure 4C/4-1.tif]

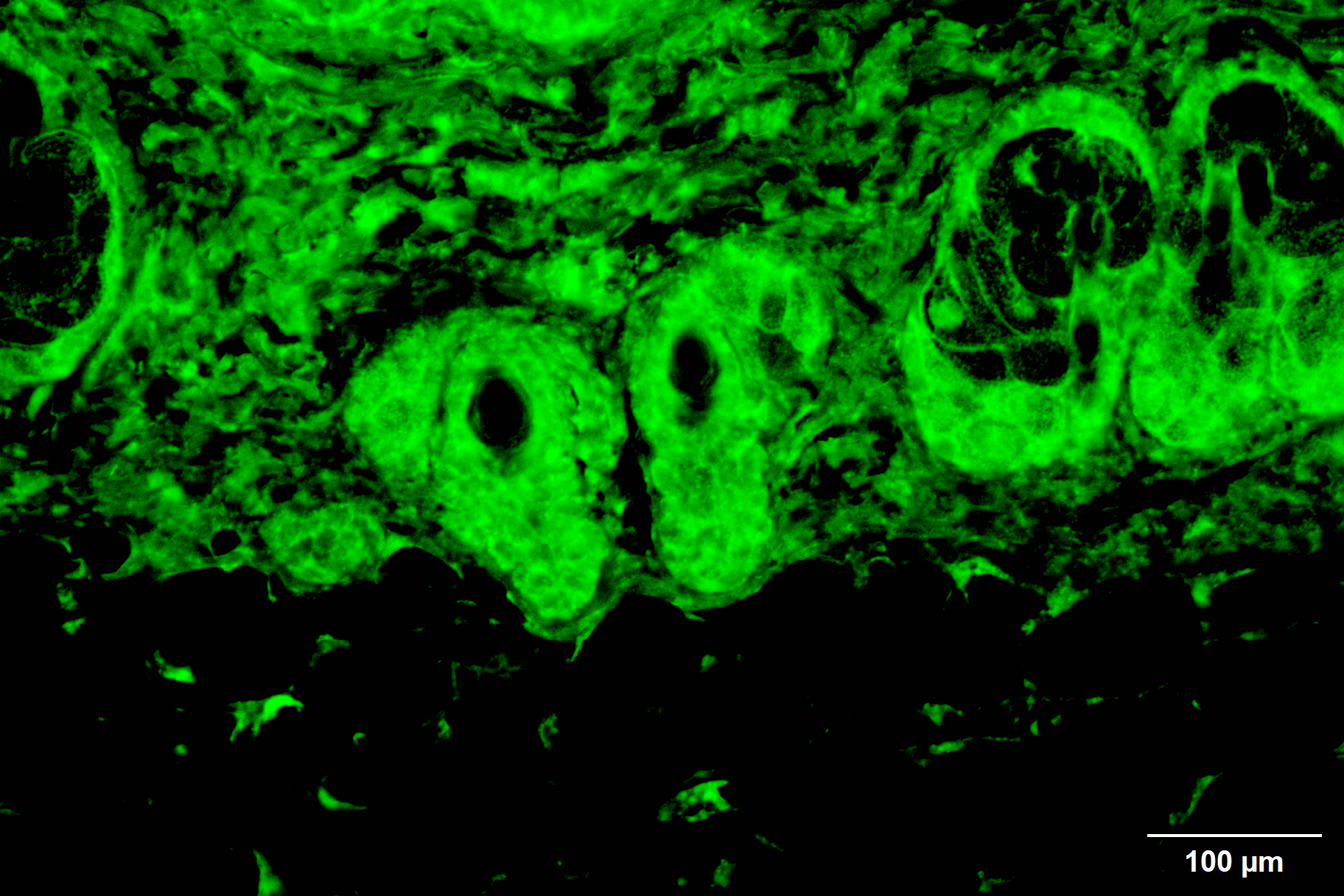

Supplement: Supplementary file 4 [file DataSheet4.zip › LA-Immunofluorescence staining image-Figure 4C/Figure 4C/4-2.tif]

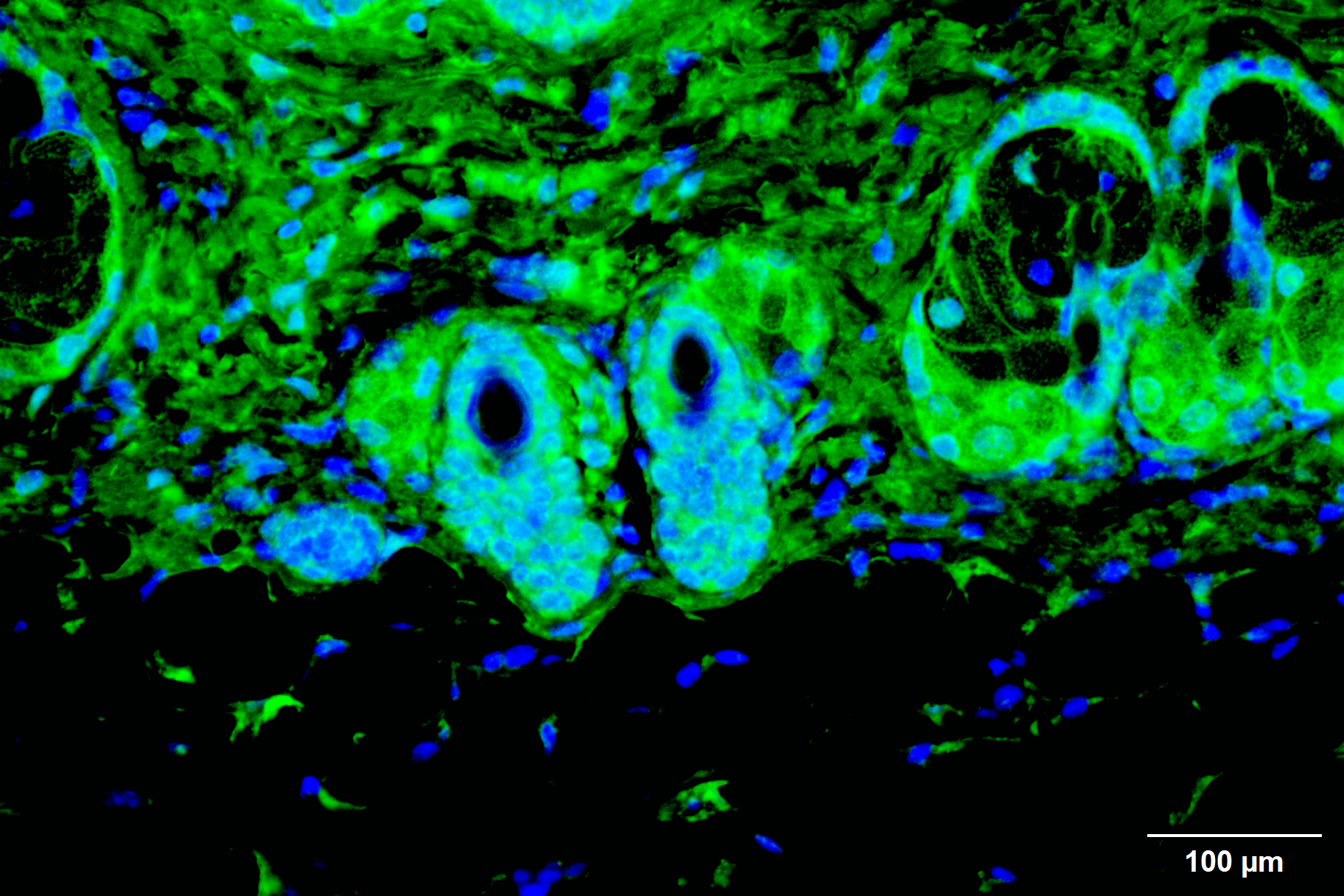

Supplement: Supplementary file 4 [file DataSheet4.zip › LA-Immunofluorescence staining image-Figure 4C/Figure 4C/4-3.tif]

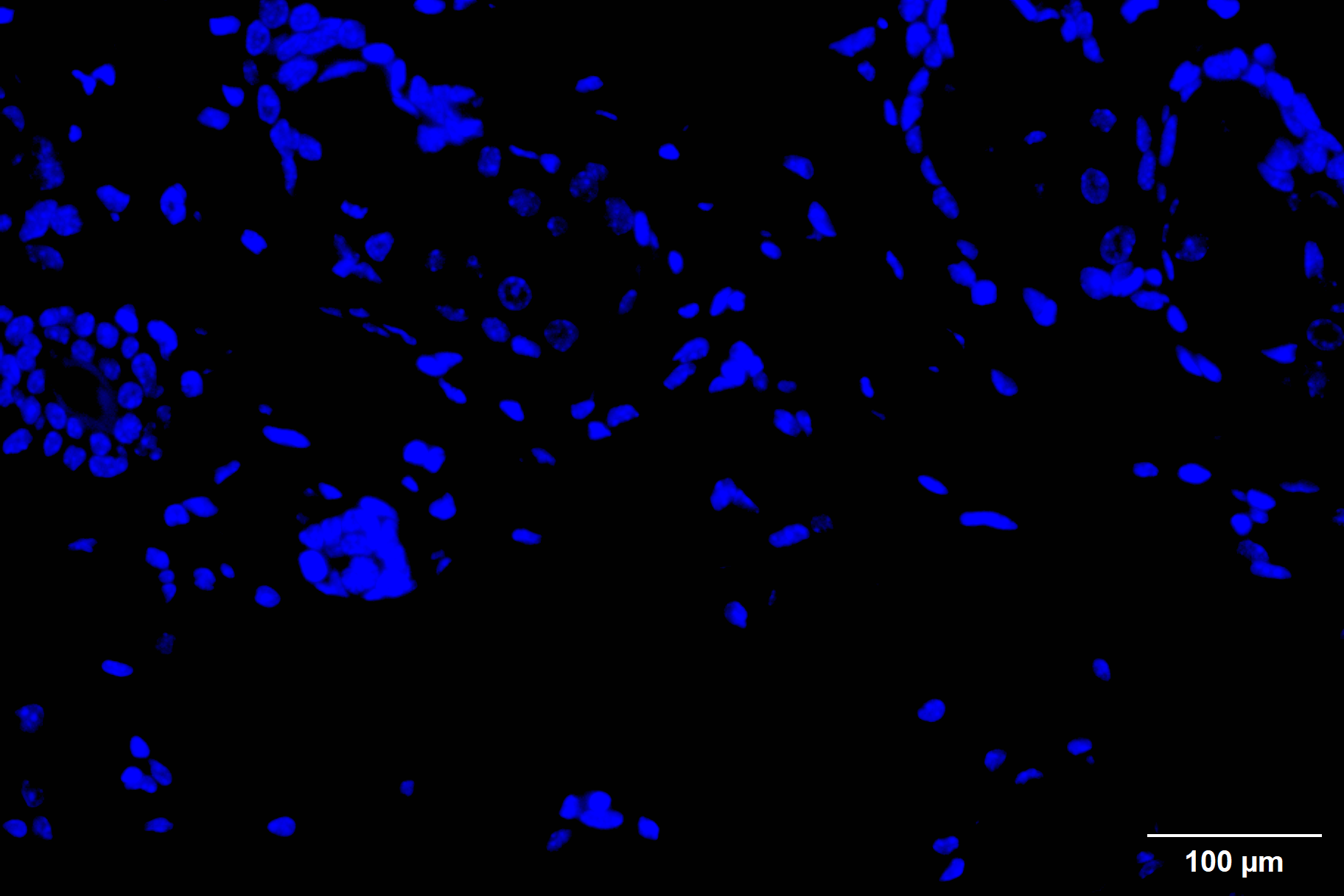

Supplement: Supplementary file 4 [file DataSheet4.zip › LA-Immunofluorescence staining image-Figure 4C/Figure 4C/5-1.tif]

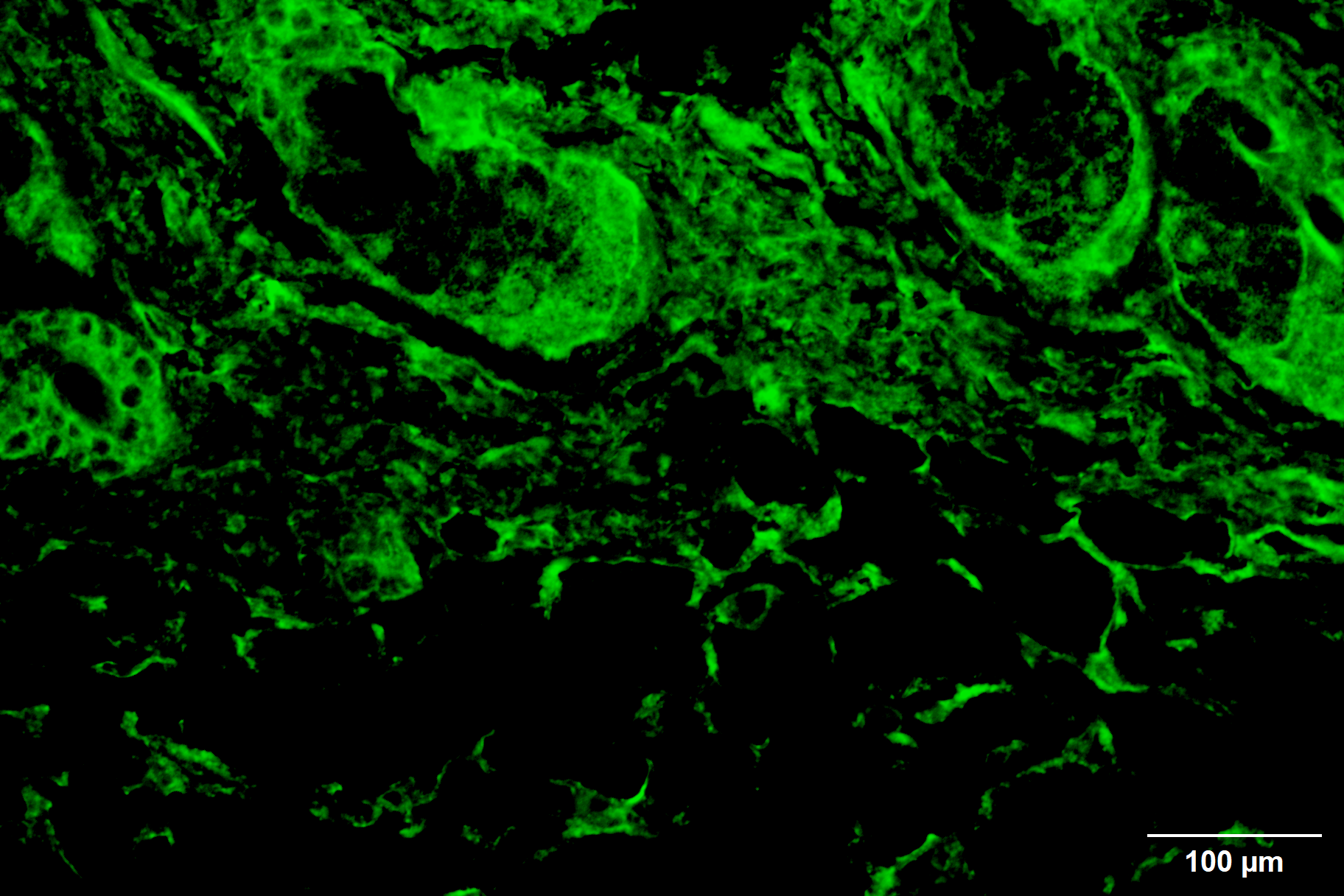

Supplement: Supplementary file 4 [file DataSheet4.zip › LA-Immunofluorescence staining image-Figure 4C/Figure 4C/5-2.tif]

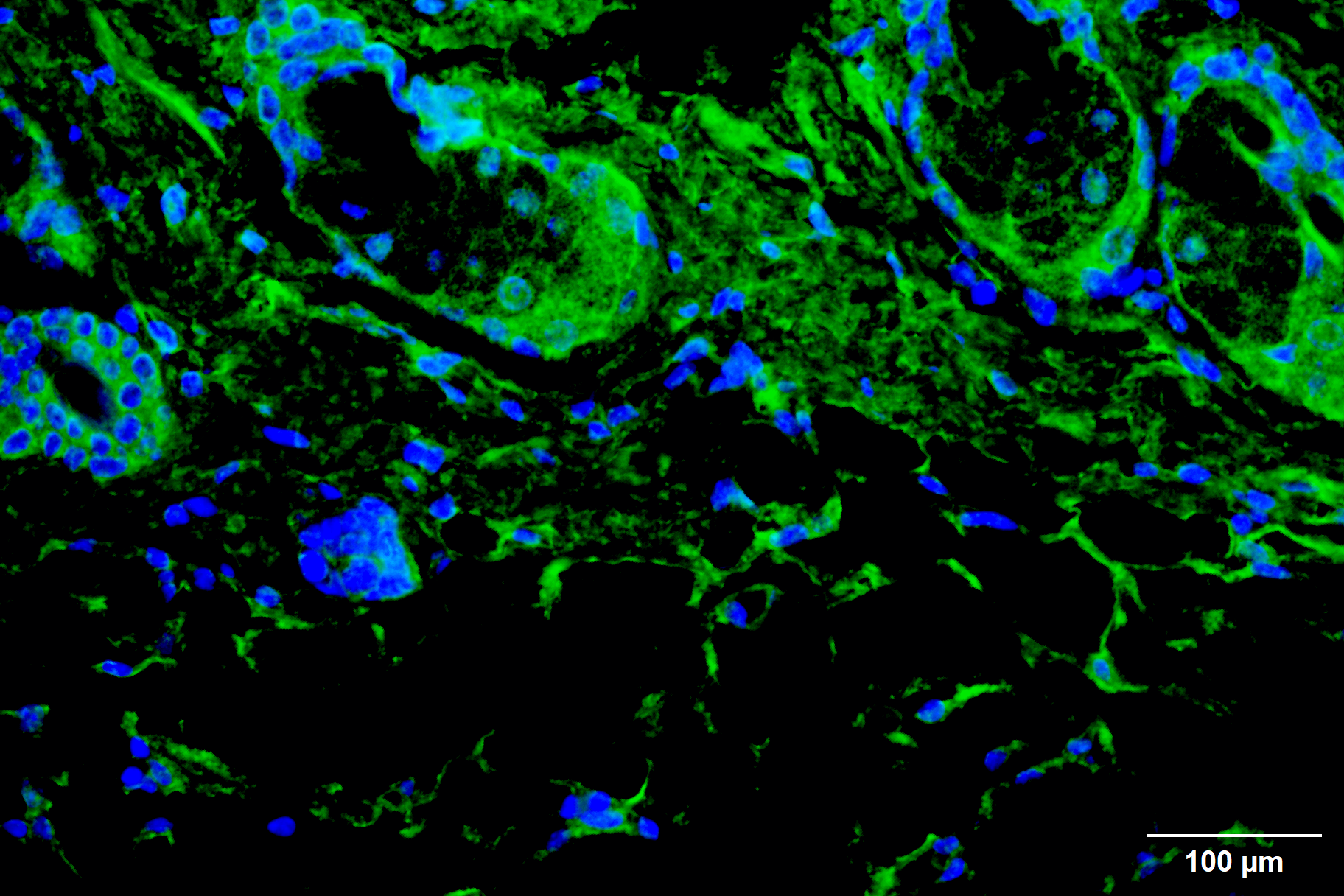

Supplement: Supplementary file 4 [file DataSheet4.zip › LA-Immunofluorescence staining image-Figure 4C/Figure 4C/5-3.tif]

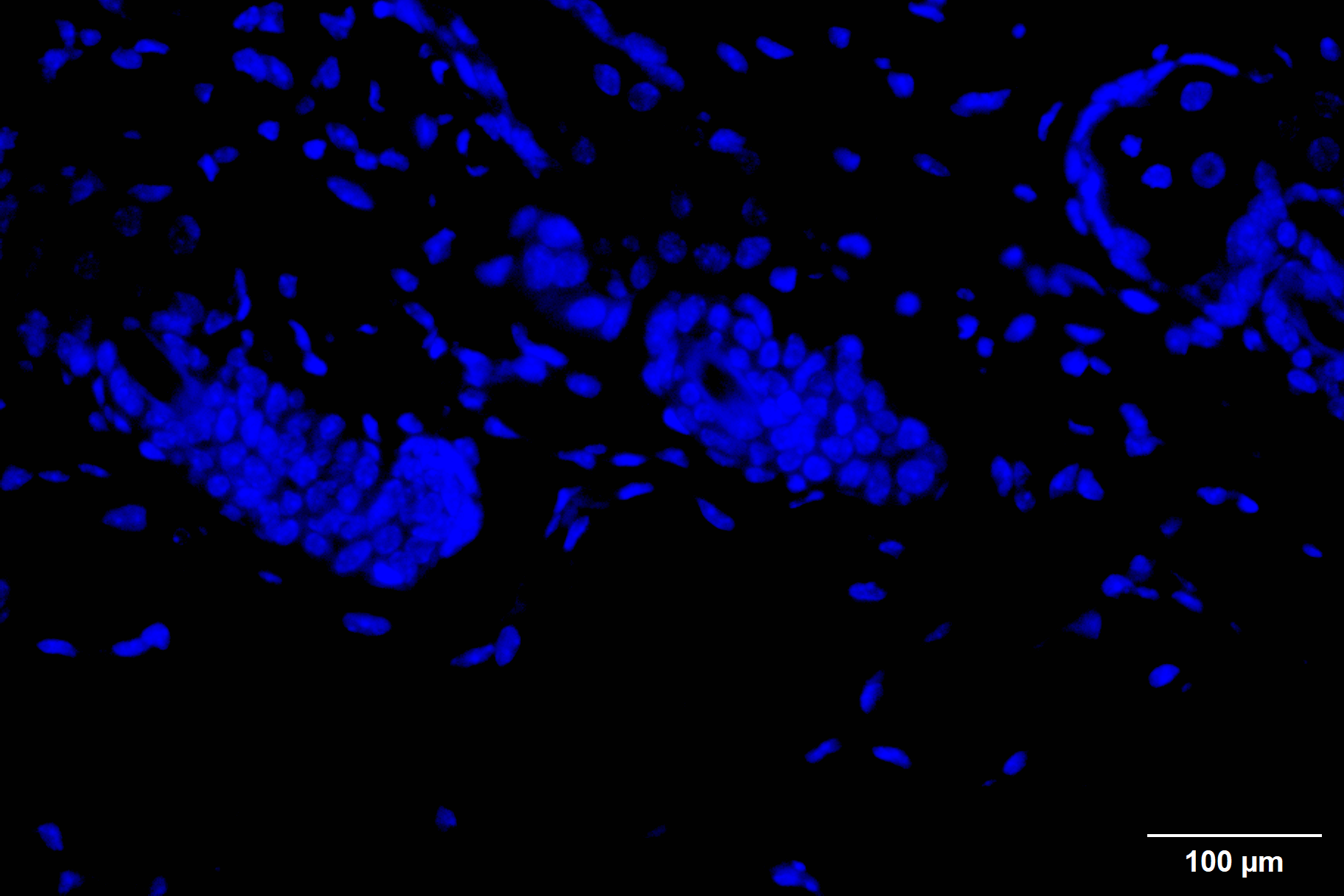

Supplement: Supplementary file 4 [file DataSheet4.zip › LA-Immunofluorescence staining image-Figure 4C/Figure 4C/6-1.tif]

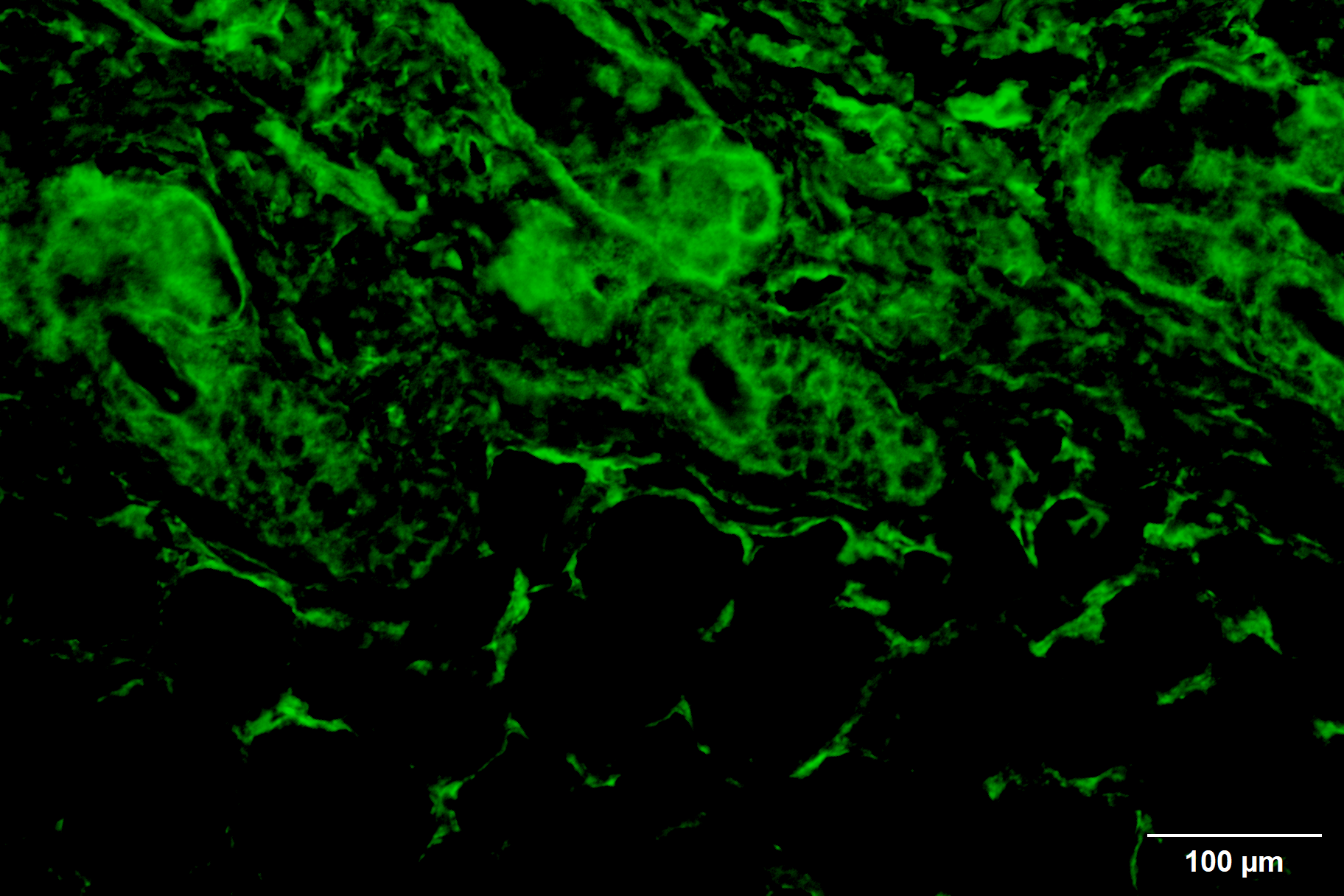

Supplement: Supplementary file 4 [file DataSheet4.zip › LA-Immunofluorescence staining image-Figure 4C/Figure 4C/6-2.tif]

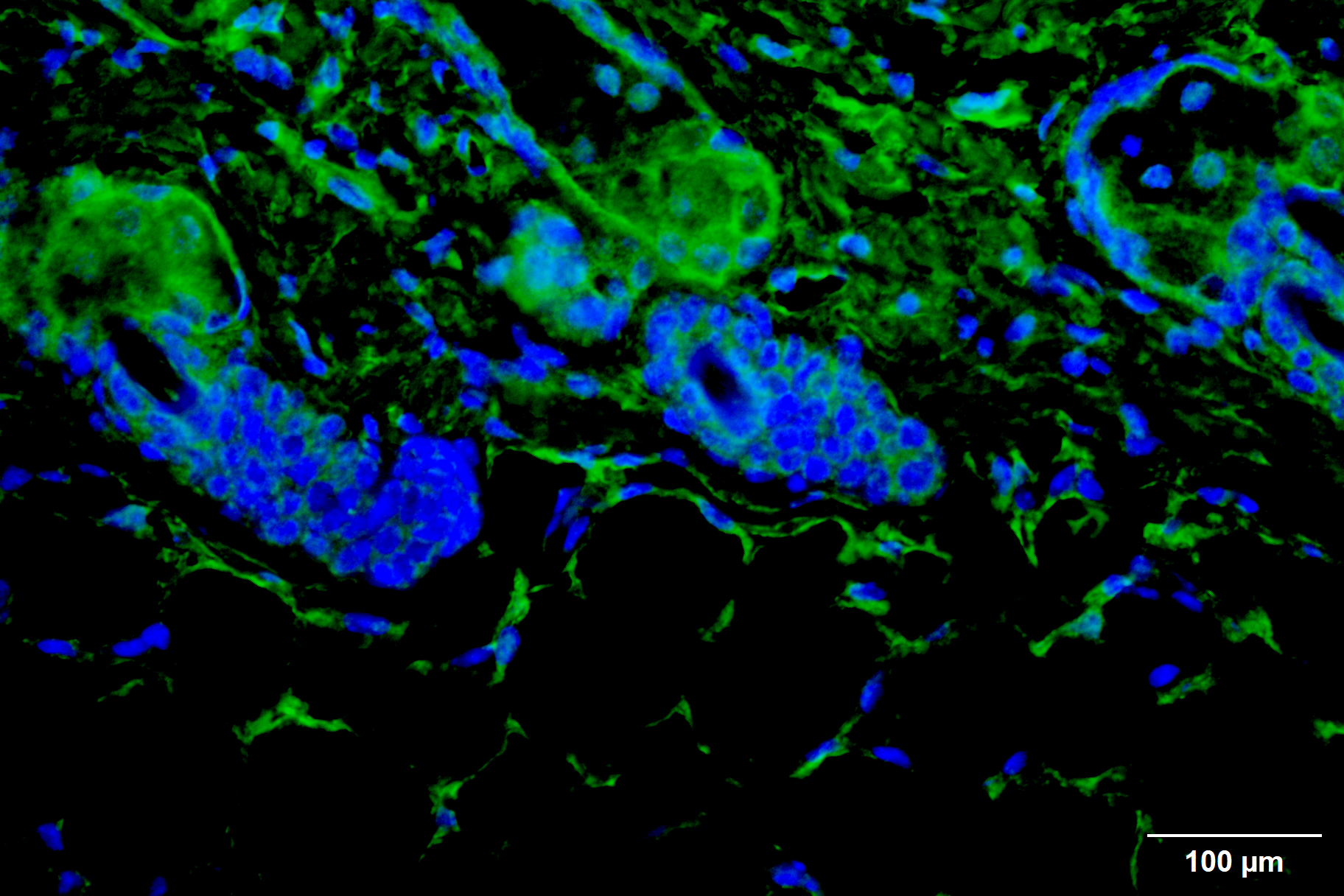

Supplement: Supplementary file 4 [file DataSheet4.zip › LA-Immunofluorescence staining image-Figure 4C/Figure 4C/6-3.tif]

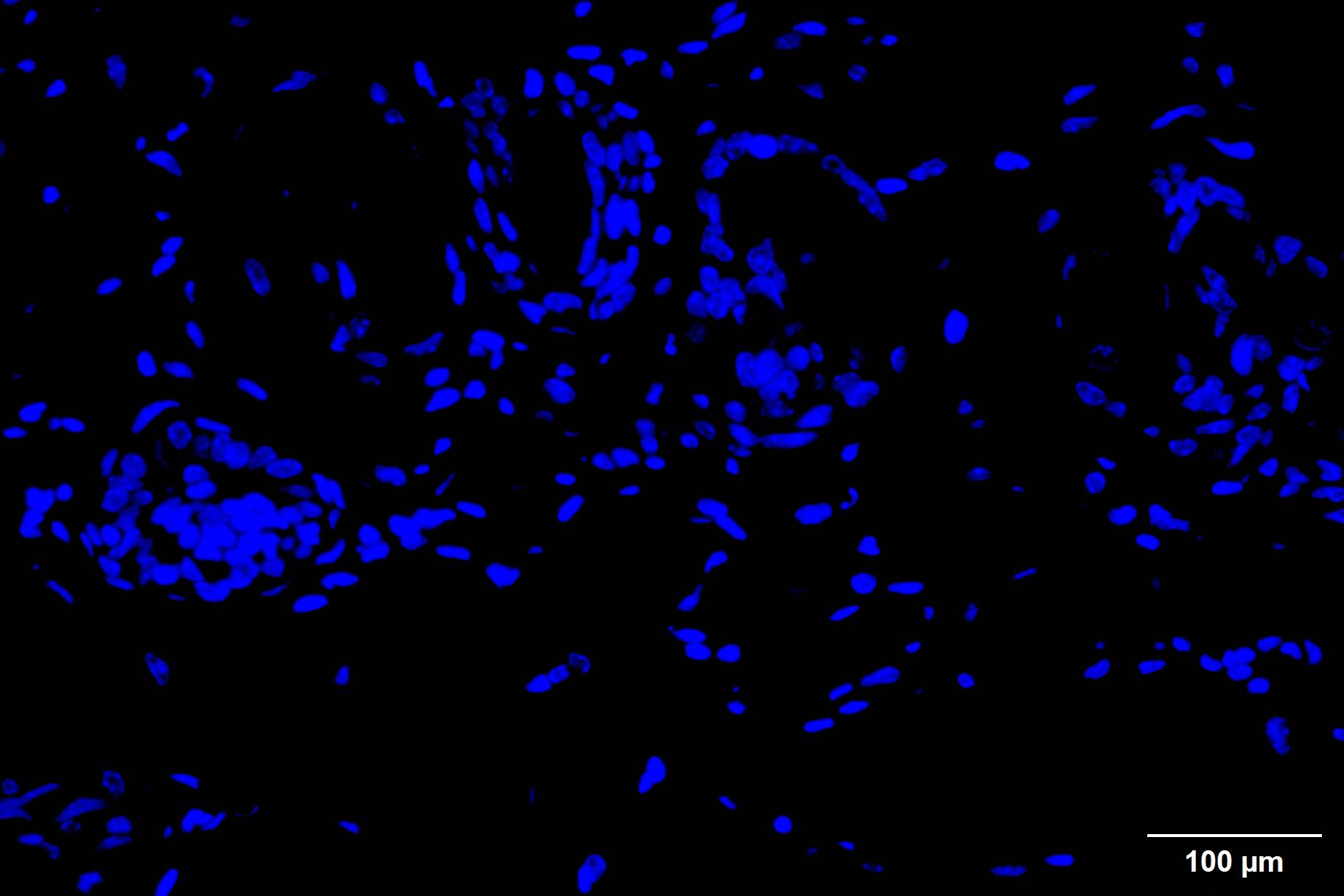

Supplement: Supplementary file 4 [file DataSheet4.zip › LA-Immunofluorescence staining image-Figure 4C/Figure 4C/7-1.tif]

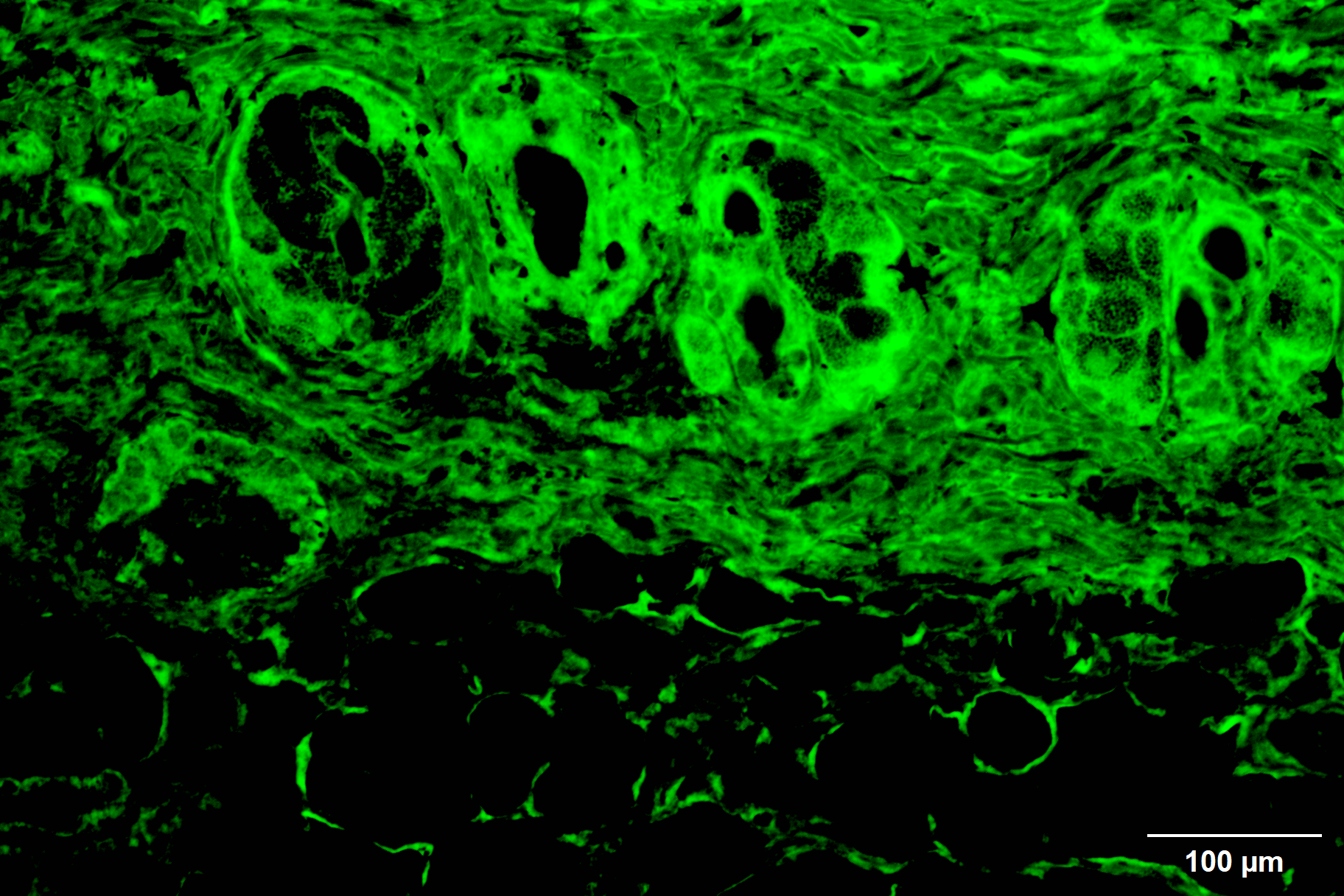

Supplement: Supplementary file 4 [file DataSheet4.zip › LA-Immunofluorescence staining image-Figure 4C/Figure 4C/7-2.tif]

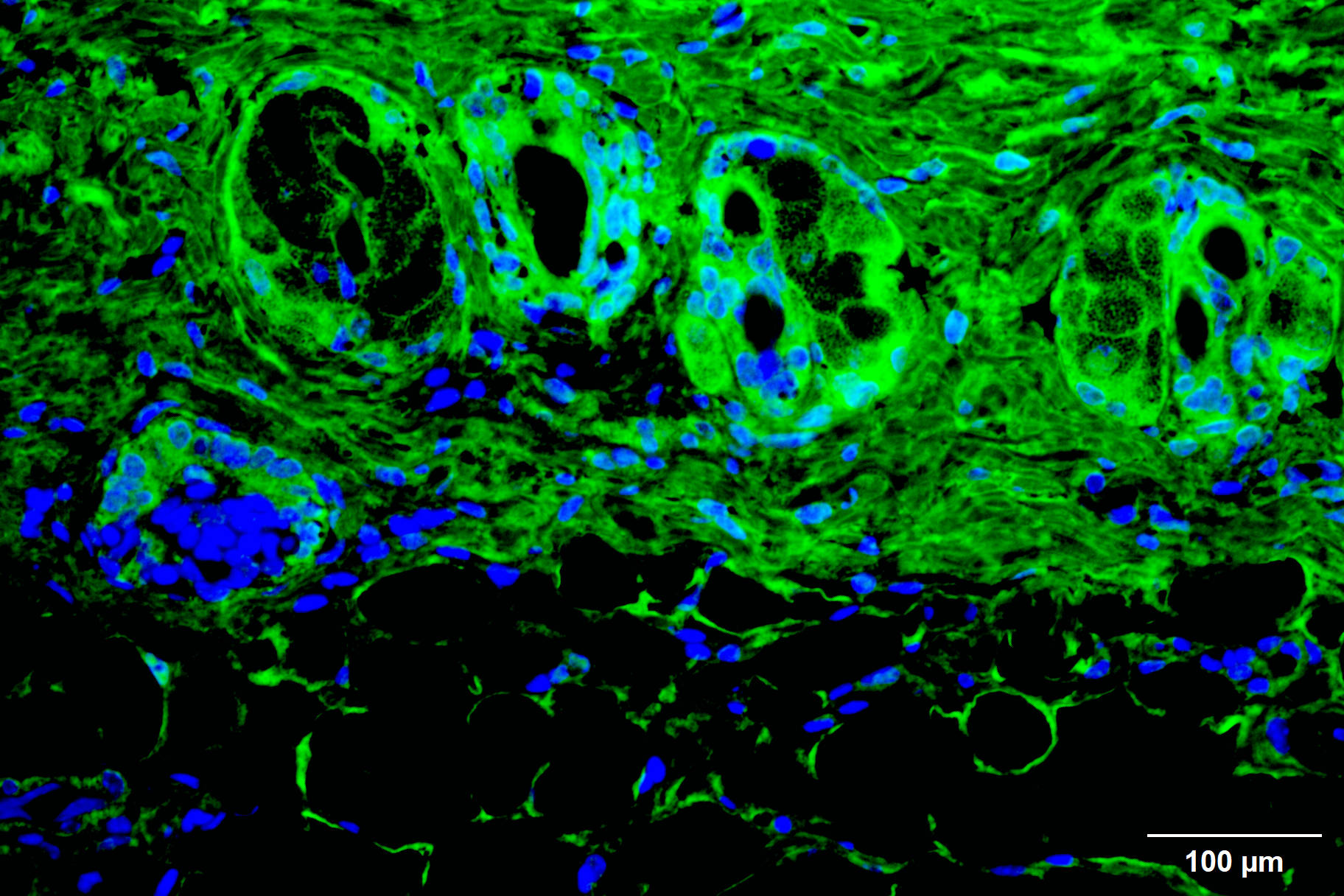

Supplement: Supplementary file 4 [file DataSheet4.zip › LA-Immunofluorescence staining image-Figure 4C/Figure 4C/7-3.tif]

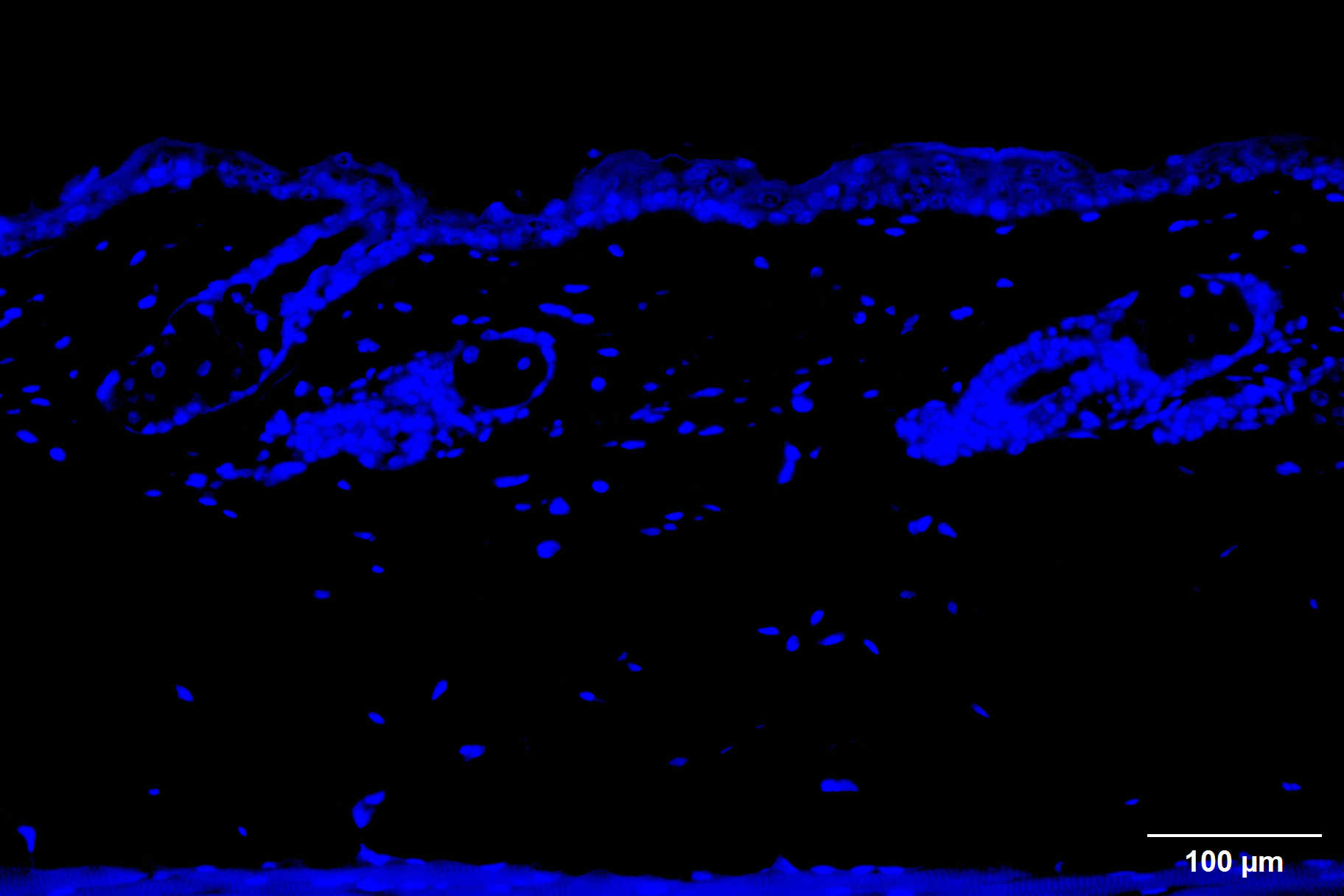

Supplement: Supplementary file 5 [file DataSheet5.zip › LA-Immunofluorescence staining image-Figure 5A/Figure 5A/1-1.tif]

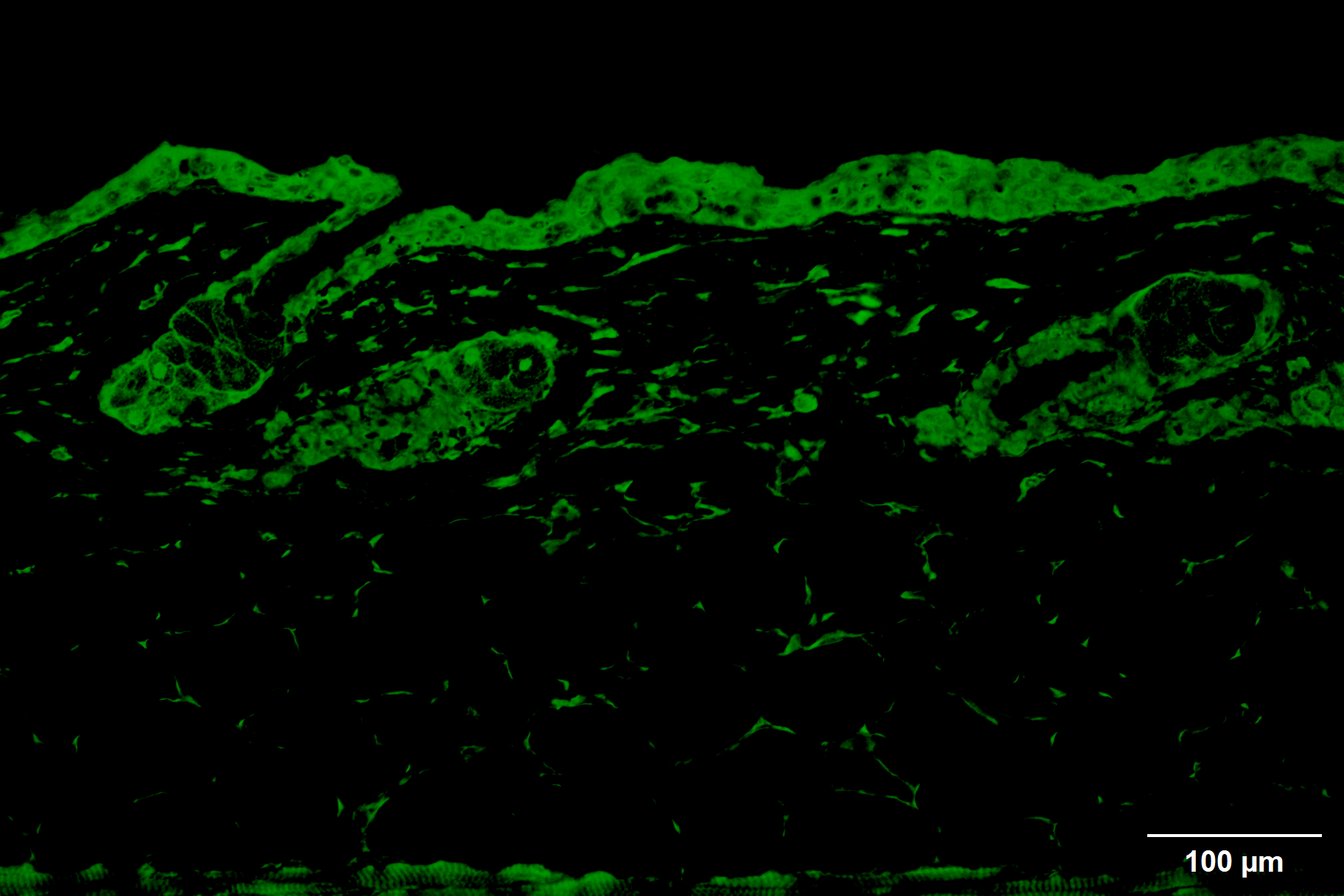

Supplement: Supplementary file 5 [file DataSheet5.zip › LA-Immunofluorescence staining image-Figure 5A/Figure 5A/1-2.tif]

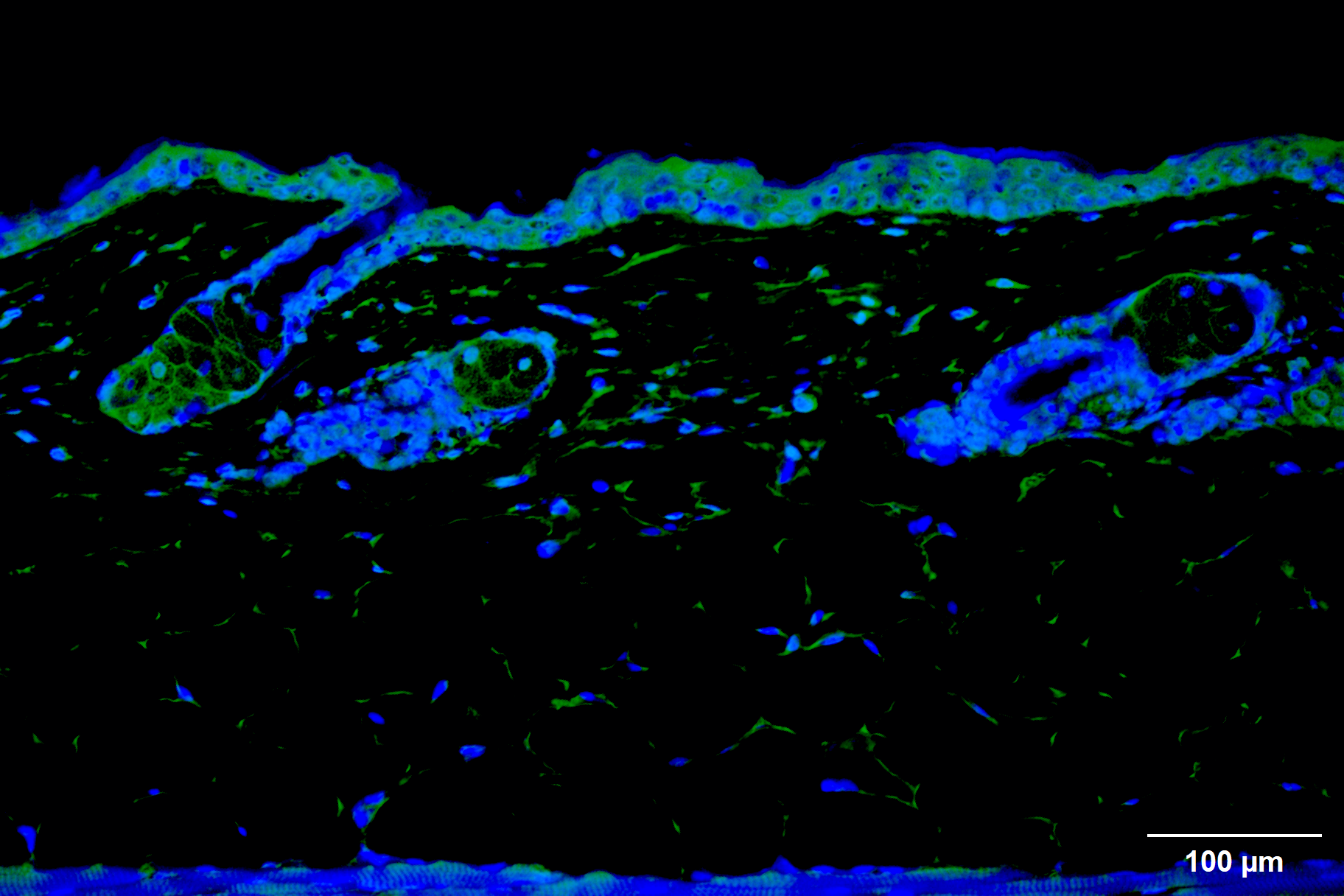

Supplement: Supplementary file 5 [file DataSheet5.zip › LA-Immunofluorescence staining image-Figure 5A/Figure 5A/1-3.tif]

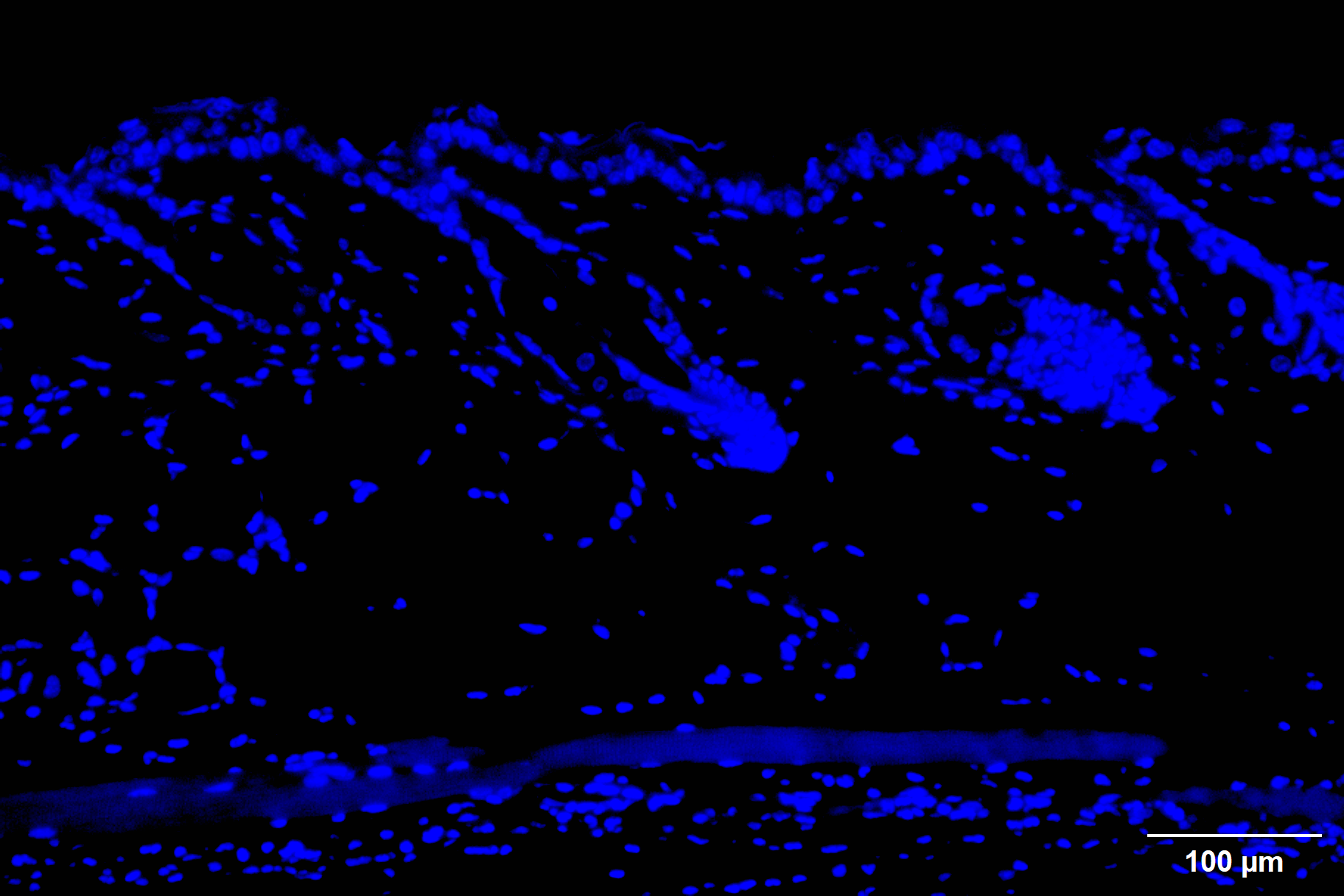

Supplement: Supplementary file 5 [file DataSheet5.zip › LA-Immunofluorescence staining image-Figure 5A/Figure 5A/2-1.tif]

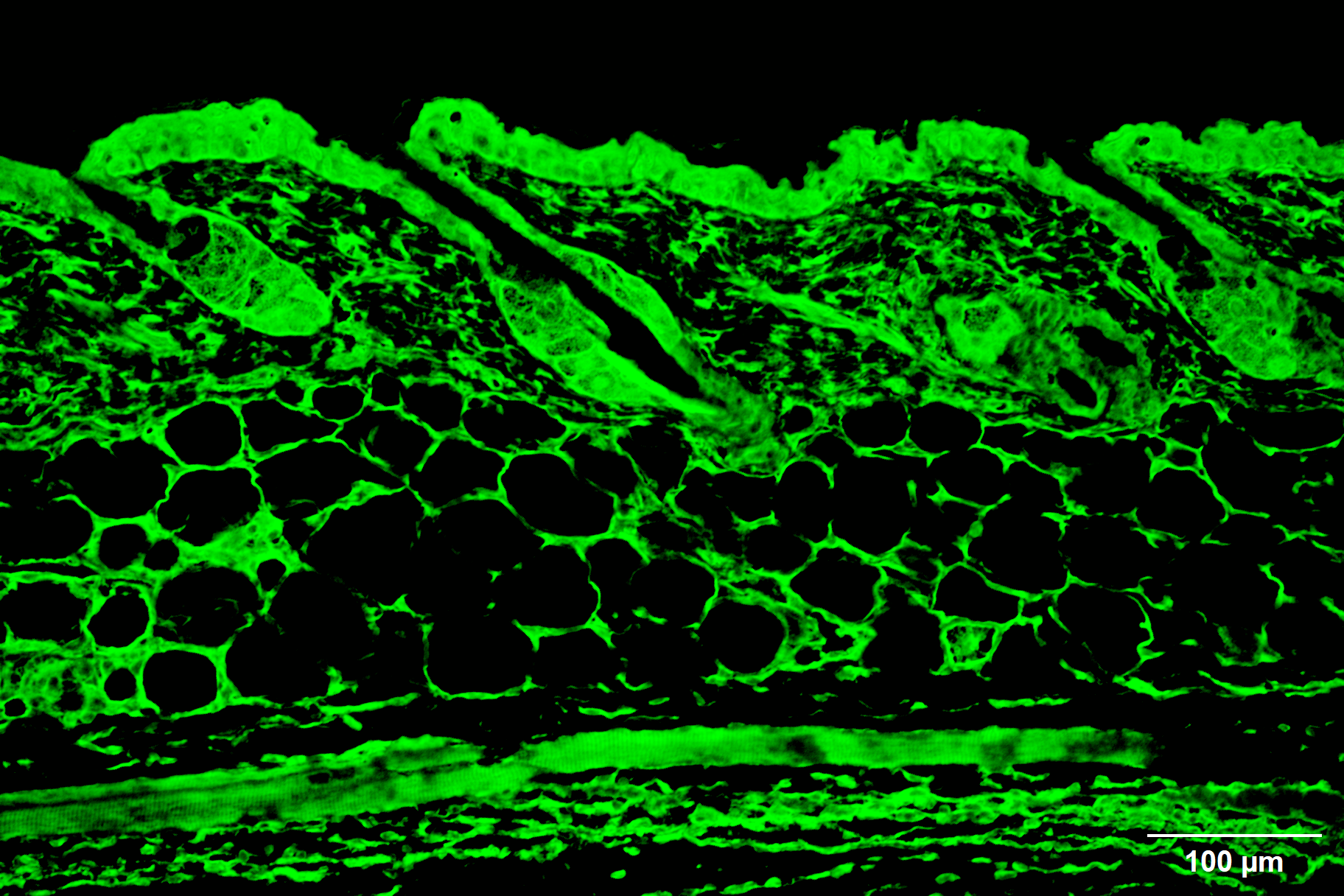

Supplement: Supplementary file 5 [file DataSheet5.zip › LA-Immunofluorescence staining image-Figure 5A/Figure 5A/2-2.tif]

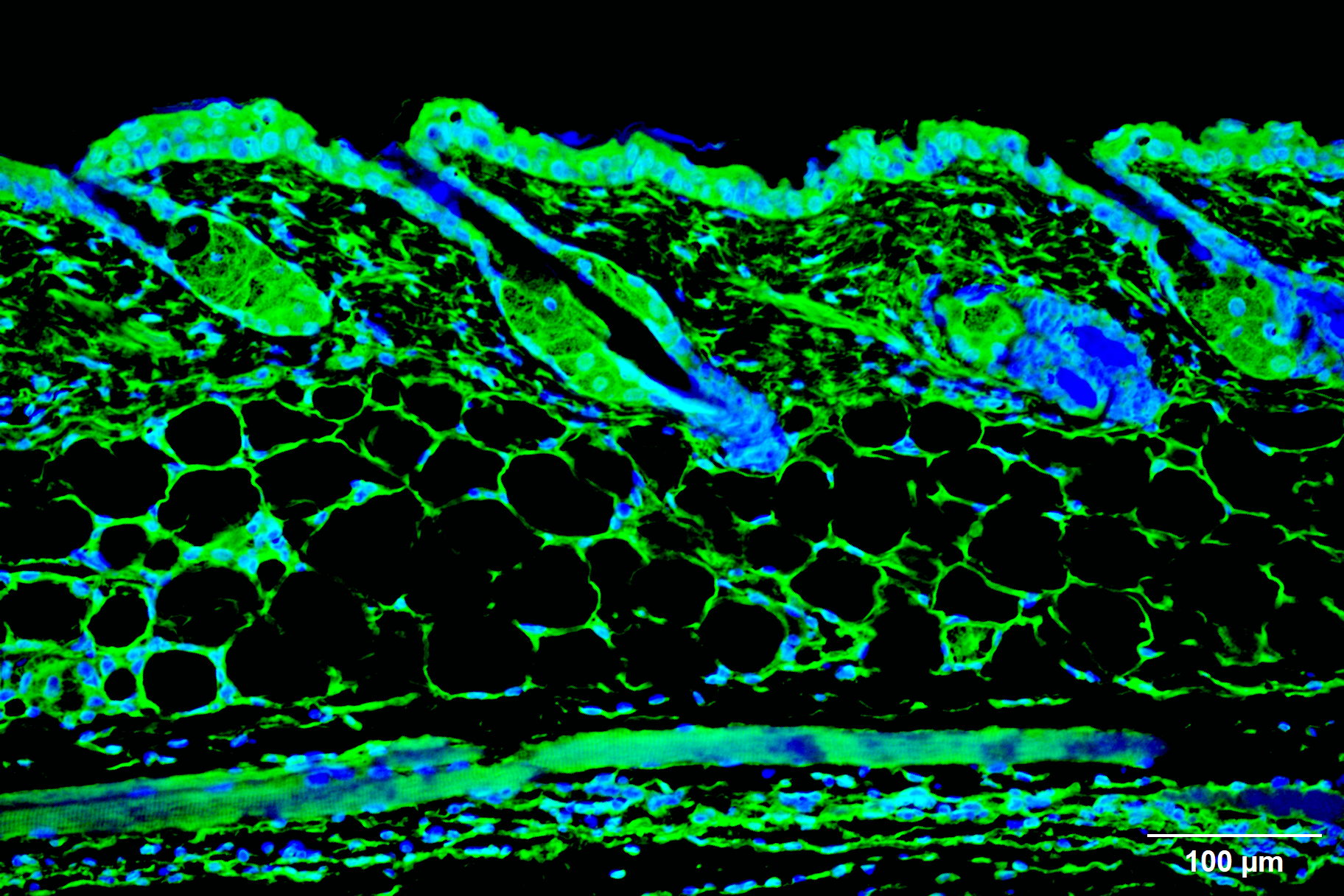

Supplement: Supplementary file 5 [file DataSheet5.zip › LA-Immunofluorescence staining image-Figure 5A/Figure 5A/2-3.tif]

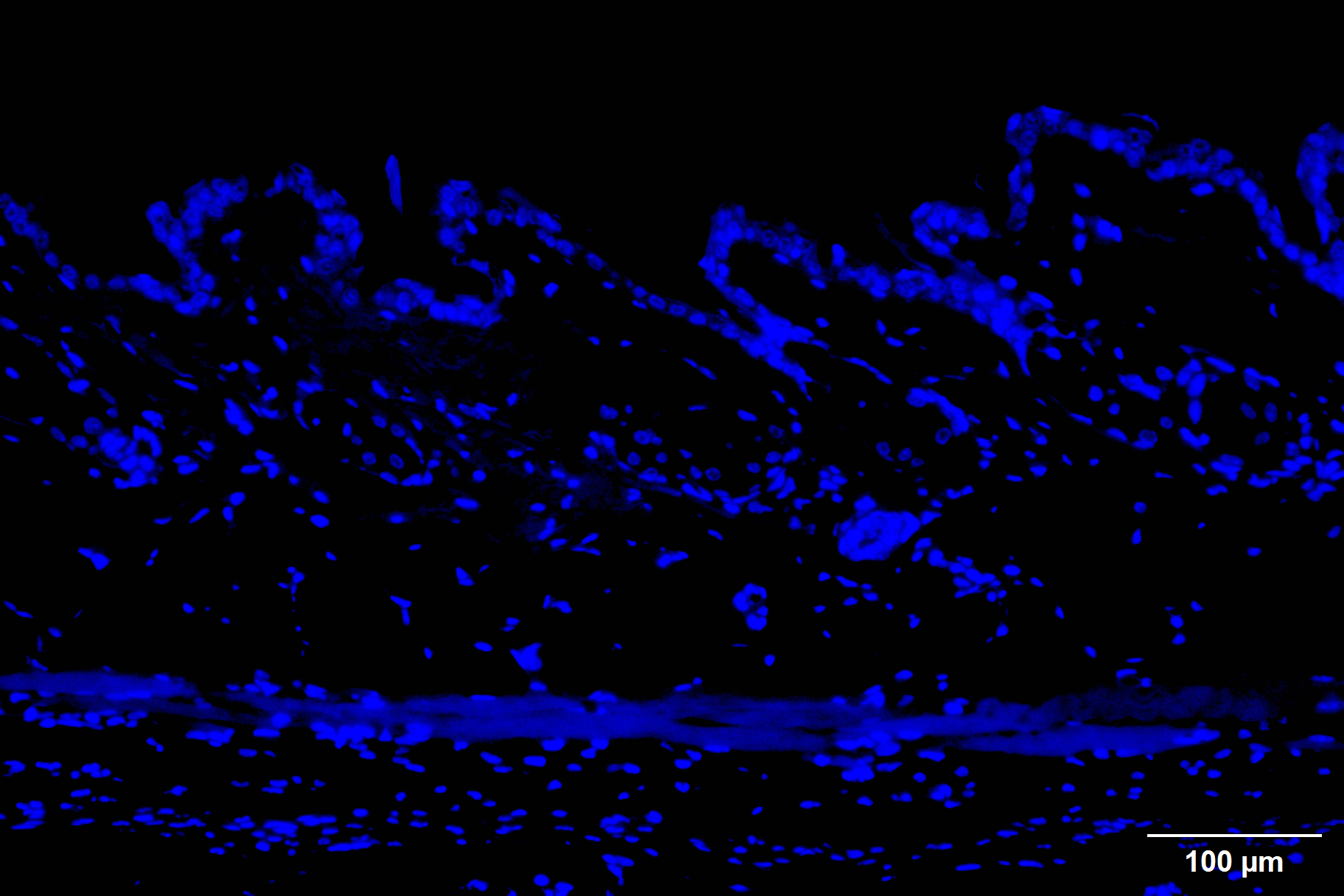

Supplement: Supplementary file 5 [file DataSheet5.zip › LA-Immunofluorescence staining image-Figure 5A/Figure 5A/3-1.tif]

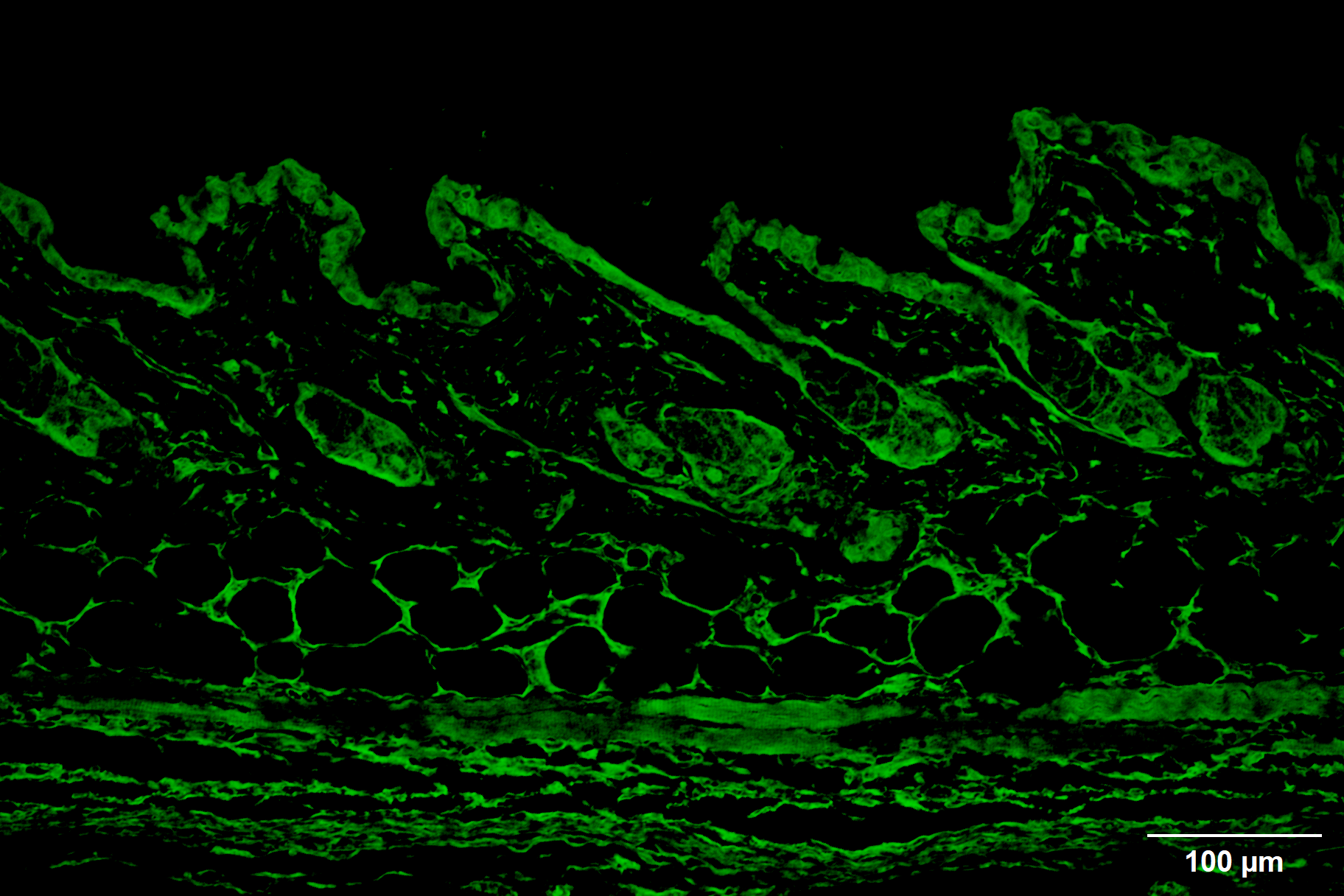

Supplement: Supplementary file 5 [file DataSheet5.zip › LA-Immunofluorescence staining image-Figure 5A/Figure 5A/3-2.tif]

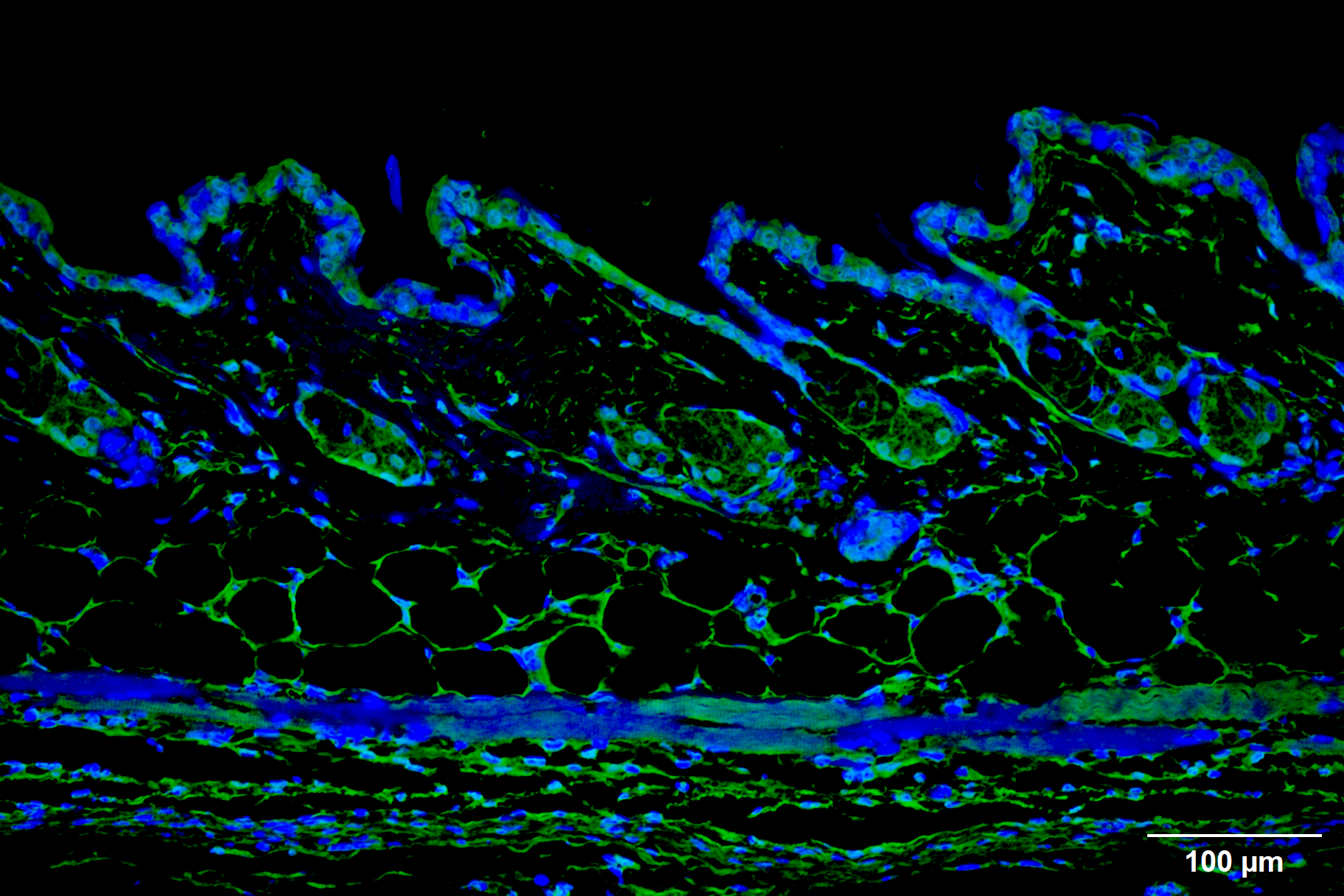

Supplement: Supplementary file 5 [file DataSheet5.zip › LA-Immunofluorescence staining image-Figure 5A/Figure 5A/3-3.tif]

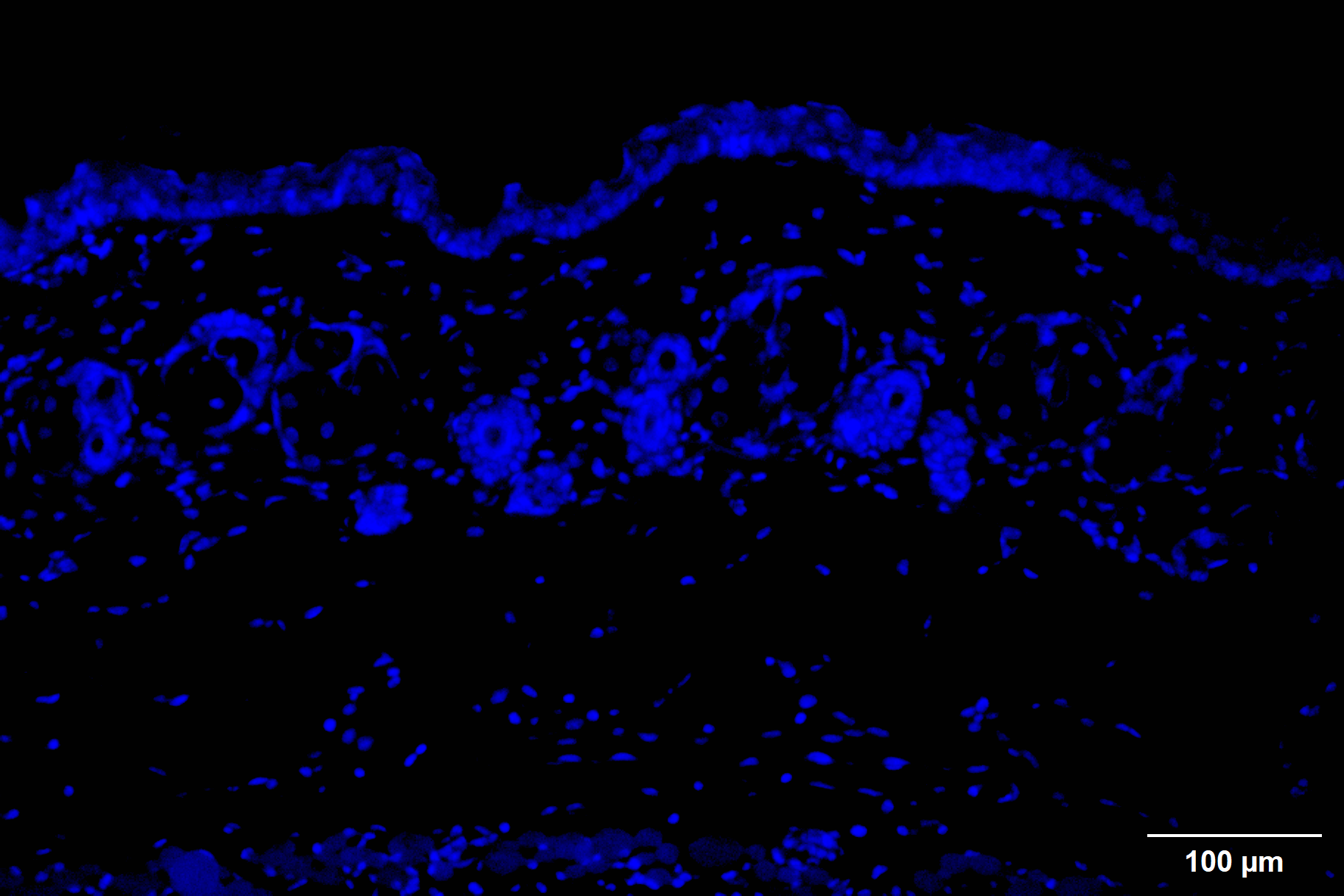

Supplement: Supplementary file 5 [file DataSheet5.zip › LA-Immunofluorescence staining image-Figure 5A/Figure 5A/4-1.tif]

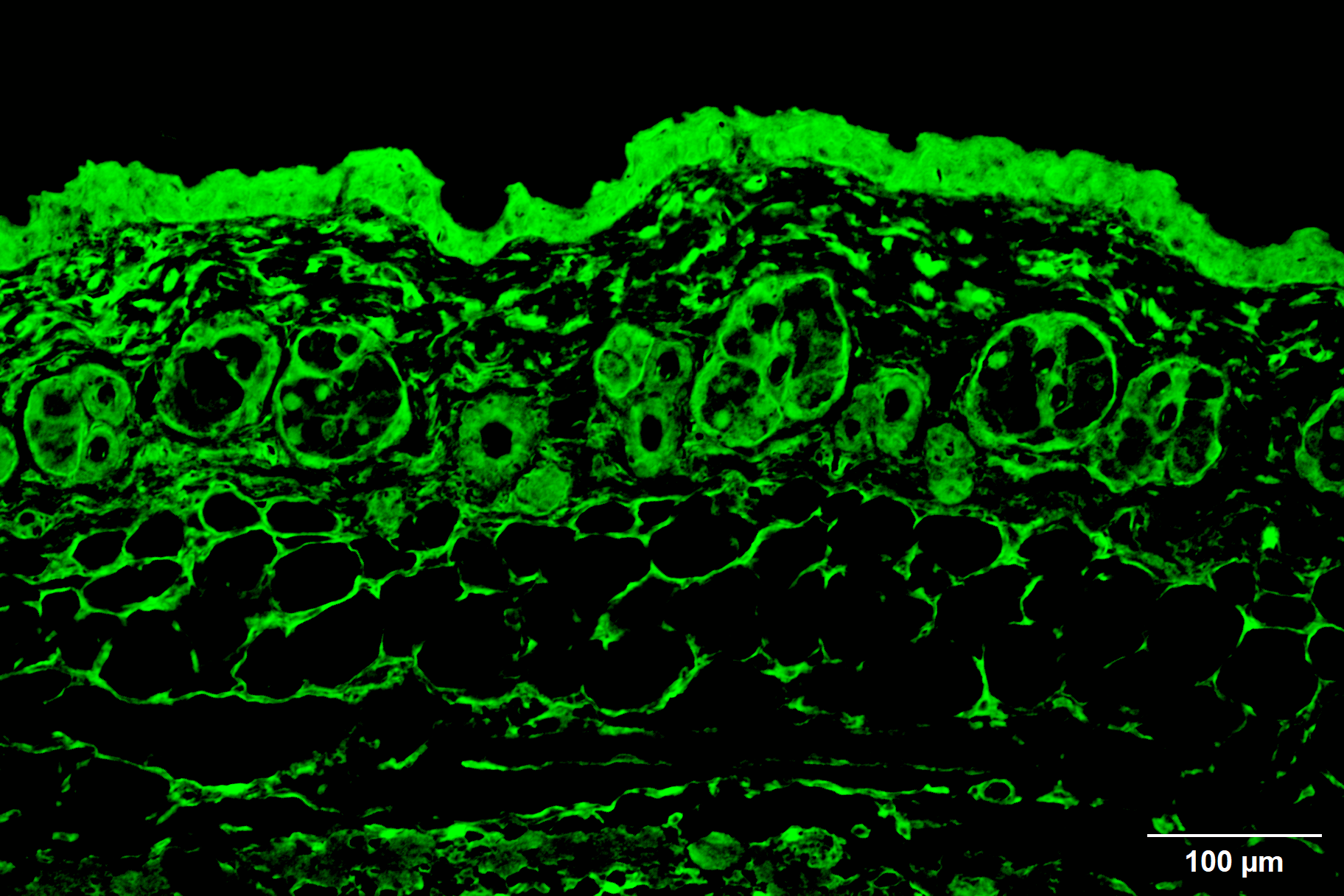

Supplement: Supplementary file 5 [file DataSheet5.zip › LA-Immunofluorescence staining image-Figure 5A/Figure 5A/4-2.tif]

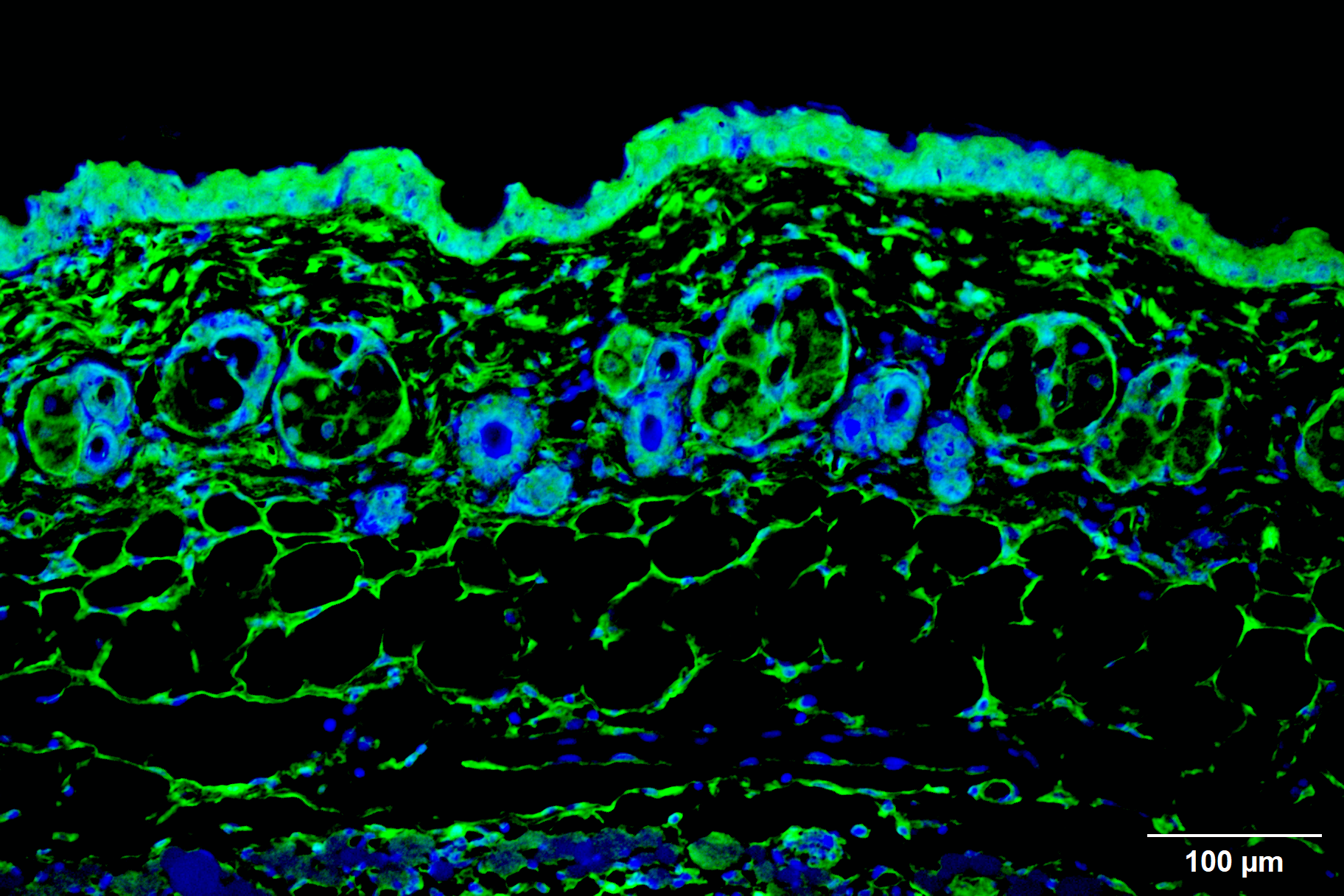

Supplement: Supplementary file 5 [file DataSheet5.zip › LA-Immunofluorescence staining image-Figure 5A/Figure 5A/4-3.tif]

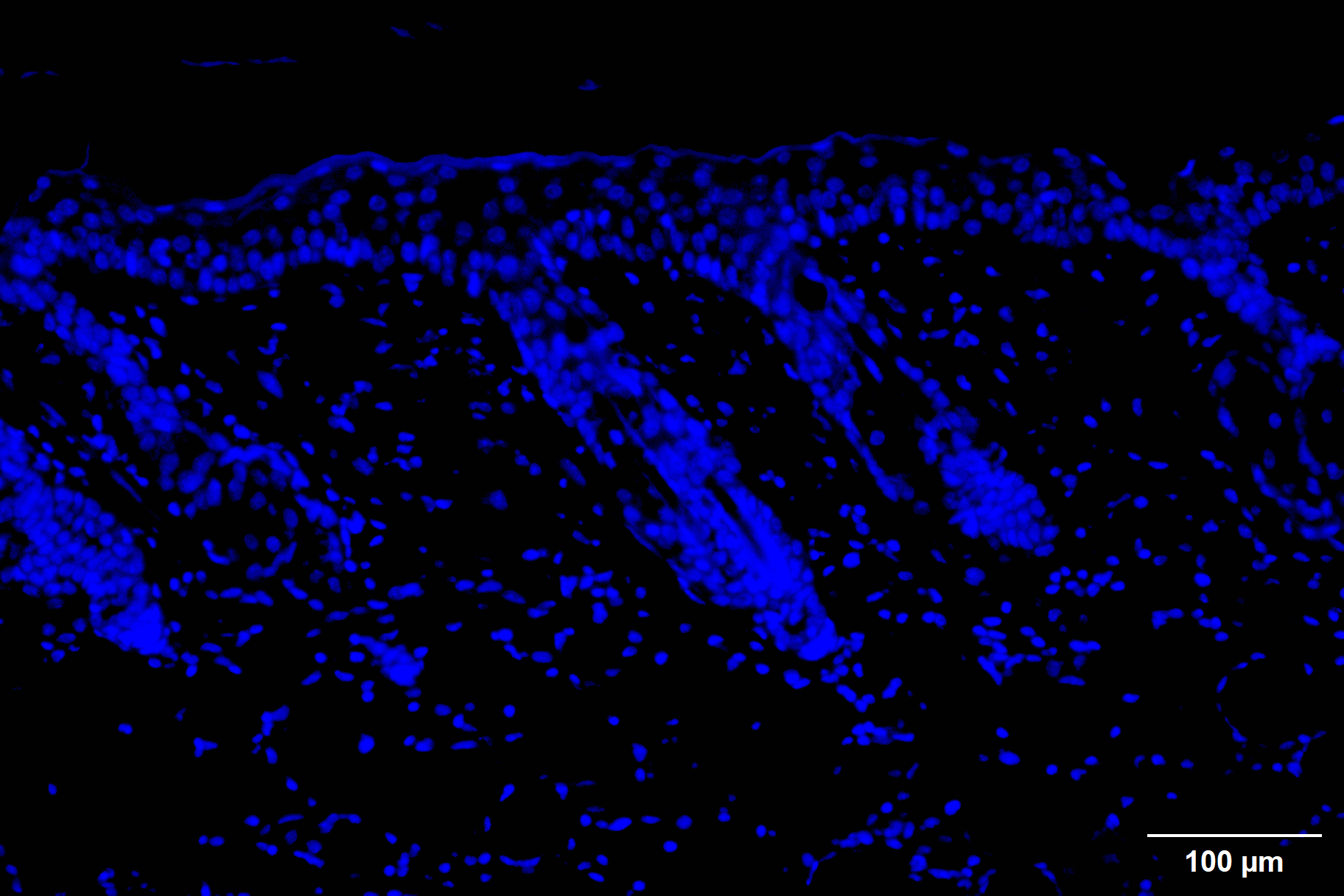

Supplement: Supplementary file 5 [file DataSheet5.zip › LA-Immunofluorescence staining image-Figure 5A/Figure 5A/5-1.tif]

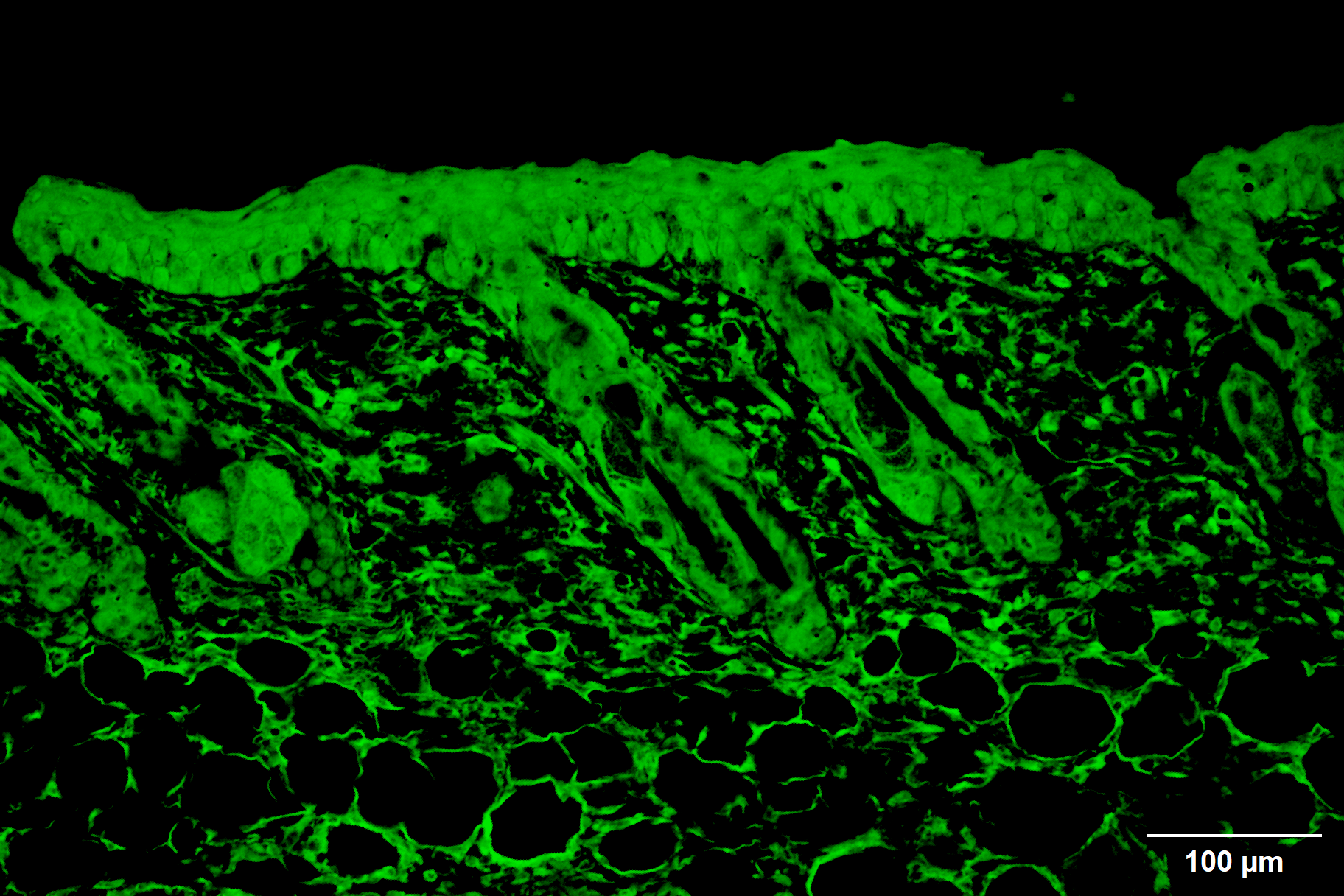

Supplement: Supplementary file 5 [file DataSheet5.zip › LA-Immunofluorescence staining image-Figure 5A/Figure 5A/5-2.tif]

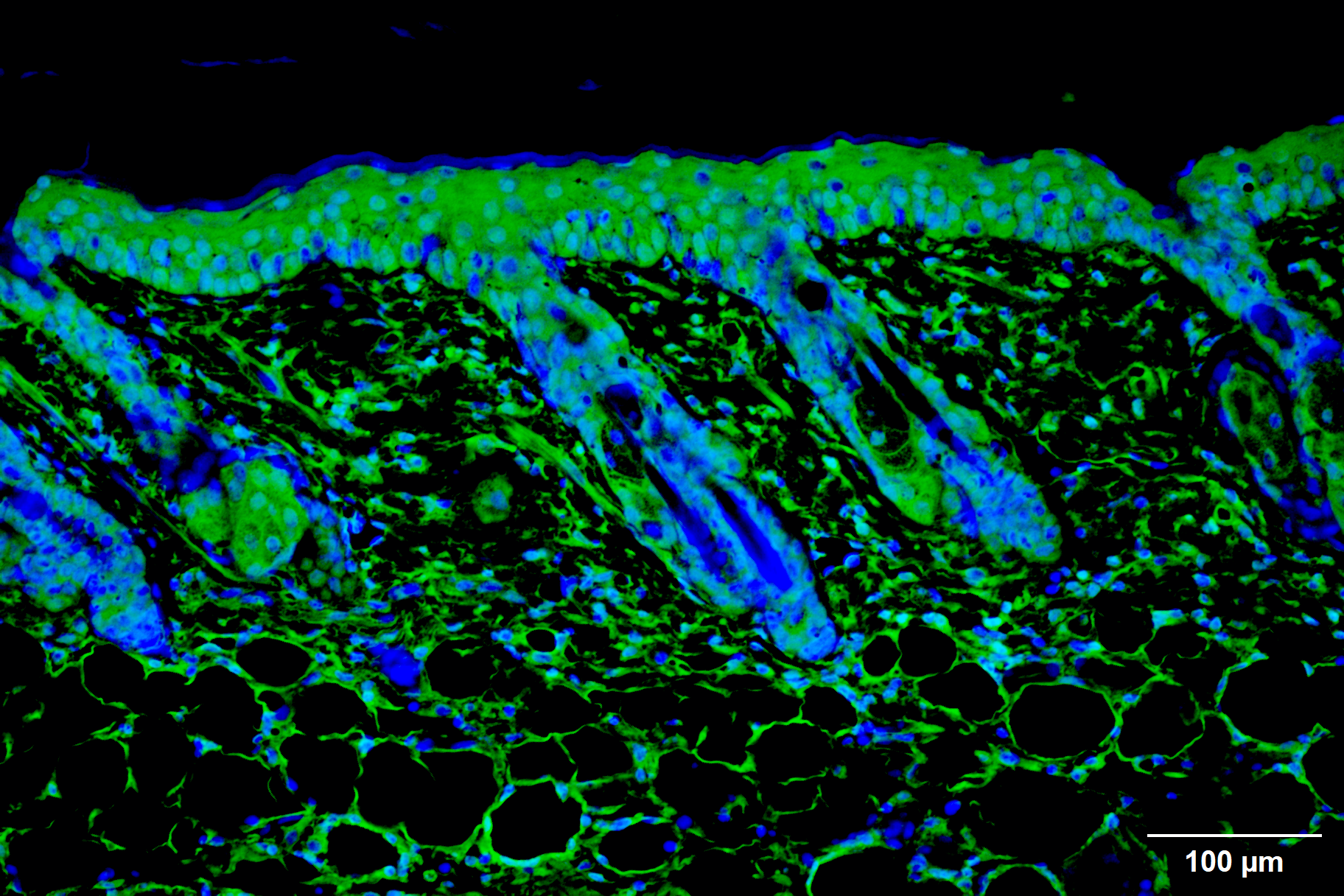

Supplement: Supplementary file 5 [file DataSheet5.zip › LA-Immunofluorescence staining image-Figure 5A/Figure 5A/5-3.tif]

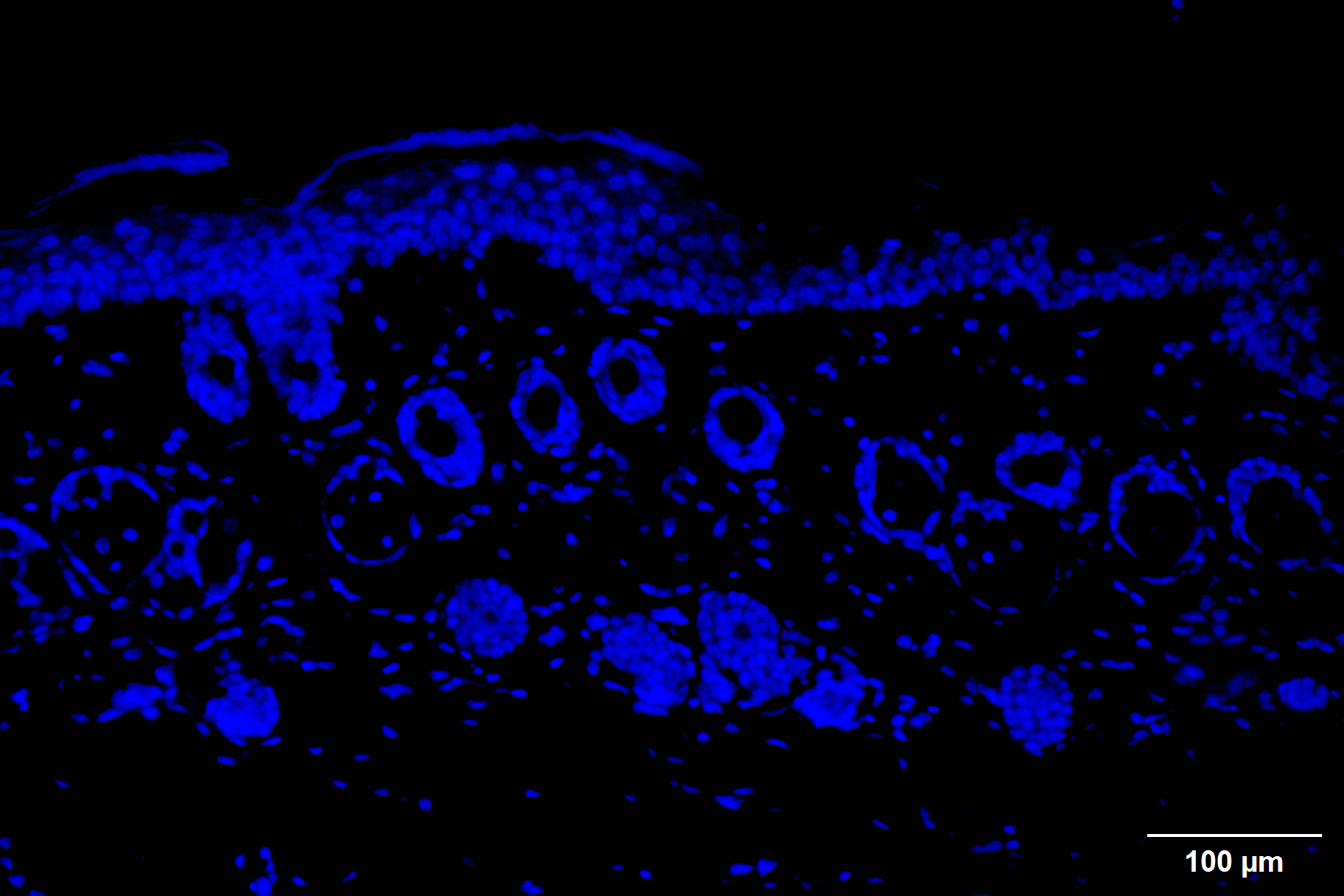

Supplement: Supplementary file 5 [file DataSheet5.zip › LA-Immunofluorescence staining image-Figure 5A/Figure 5A/6-1.tif]

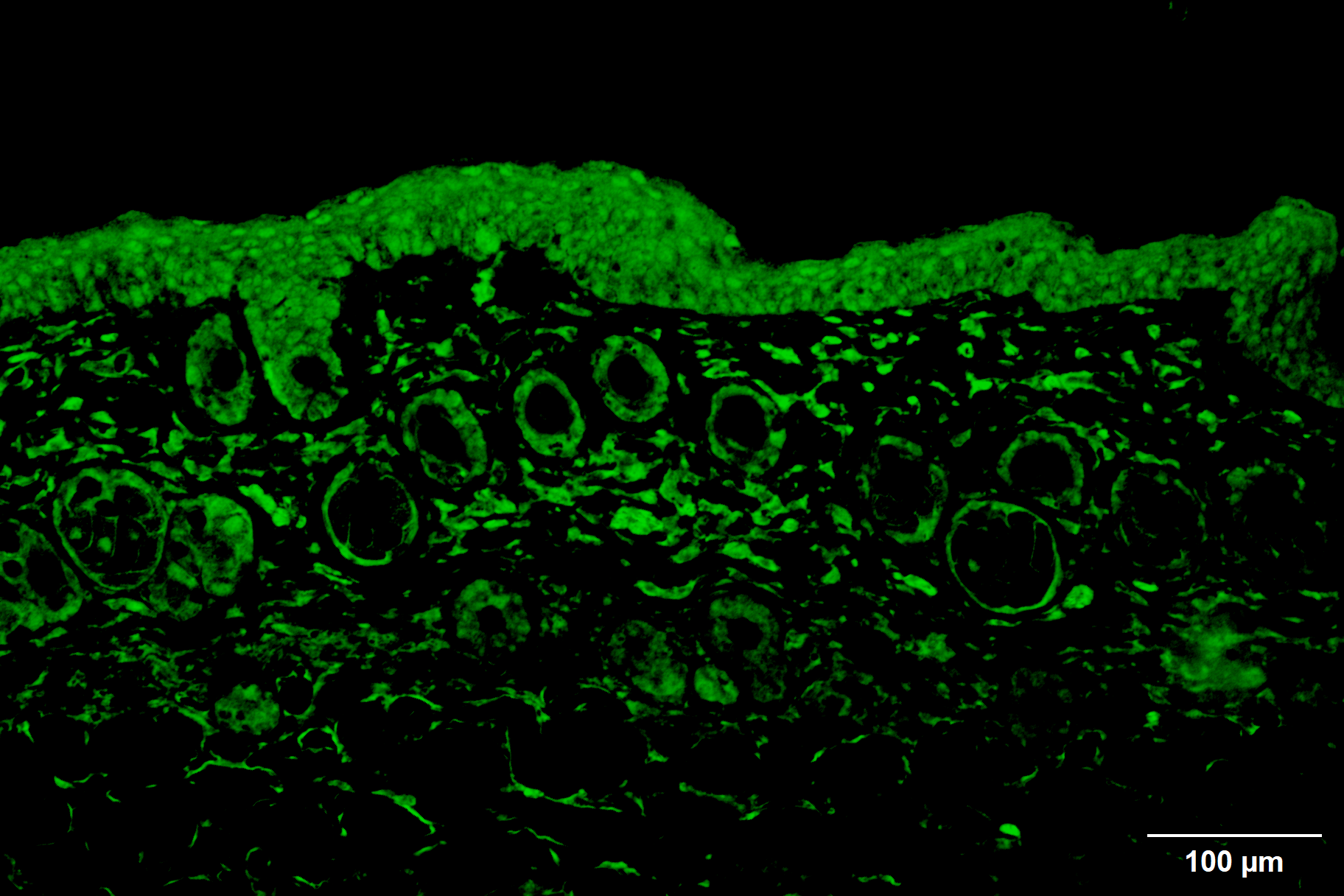

Supplement: Supplementary file 5 [file DataSheet5.zip › LA-Immunofluorescence staining image-Figure 5A/Figure 5A/6-2.tif]

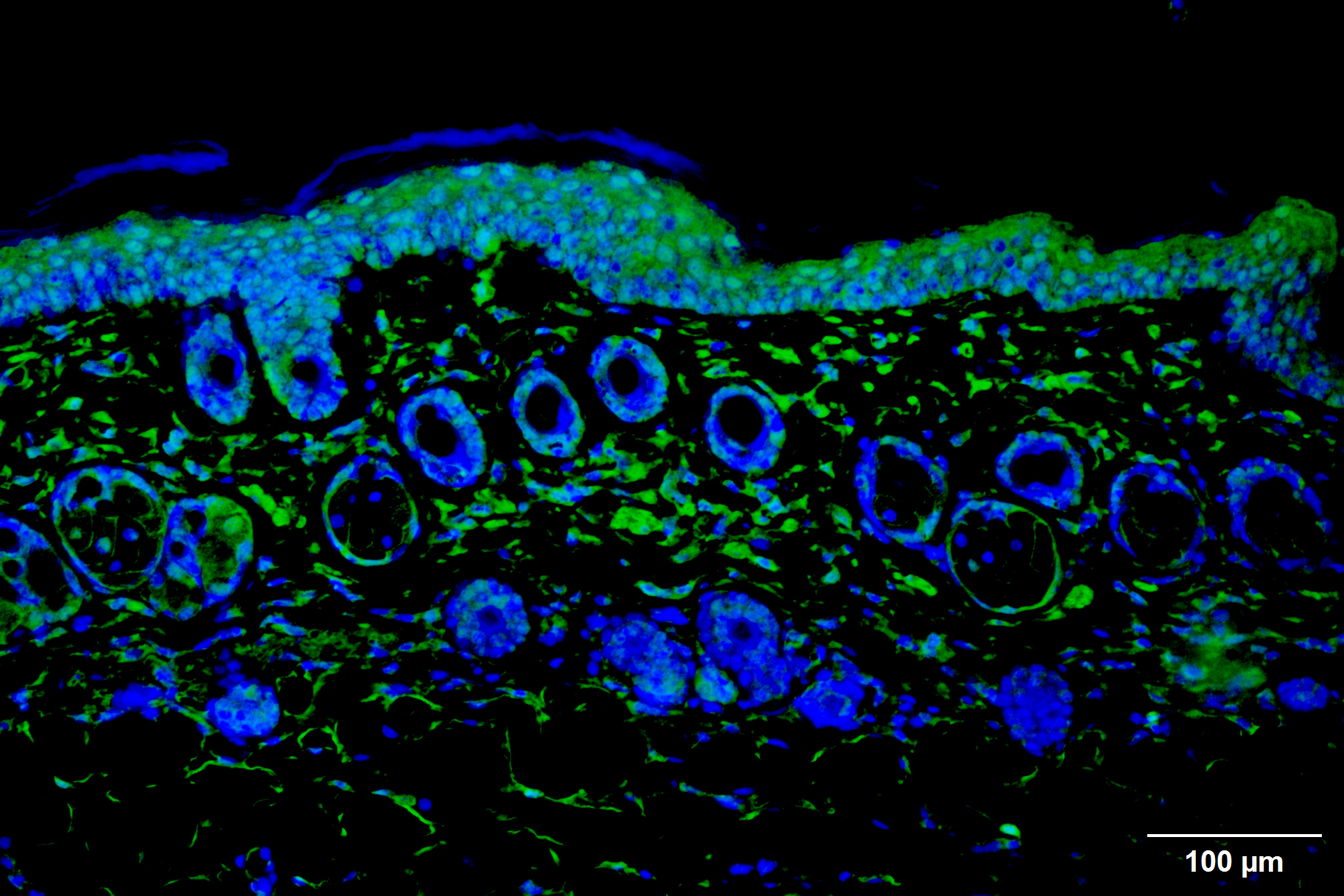

Supplement: Supplementary file 5 [file DataSheet5.zip › LA-Immunofluorescence staining image-Figure 5A/Figure 5A/6-3.tif]

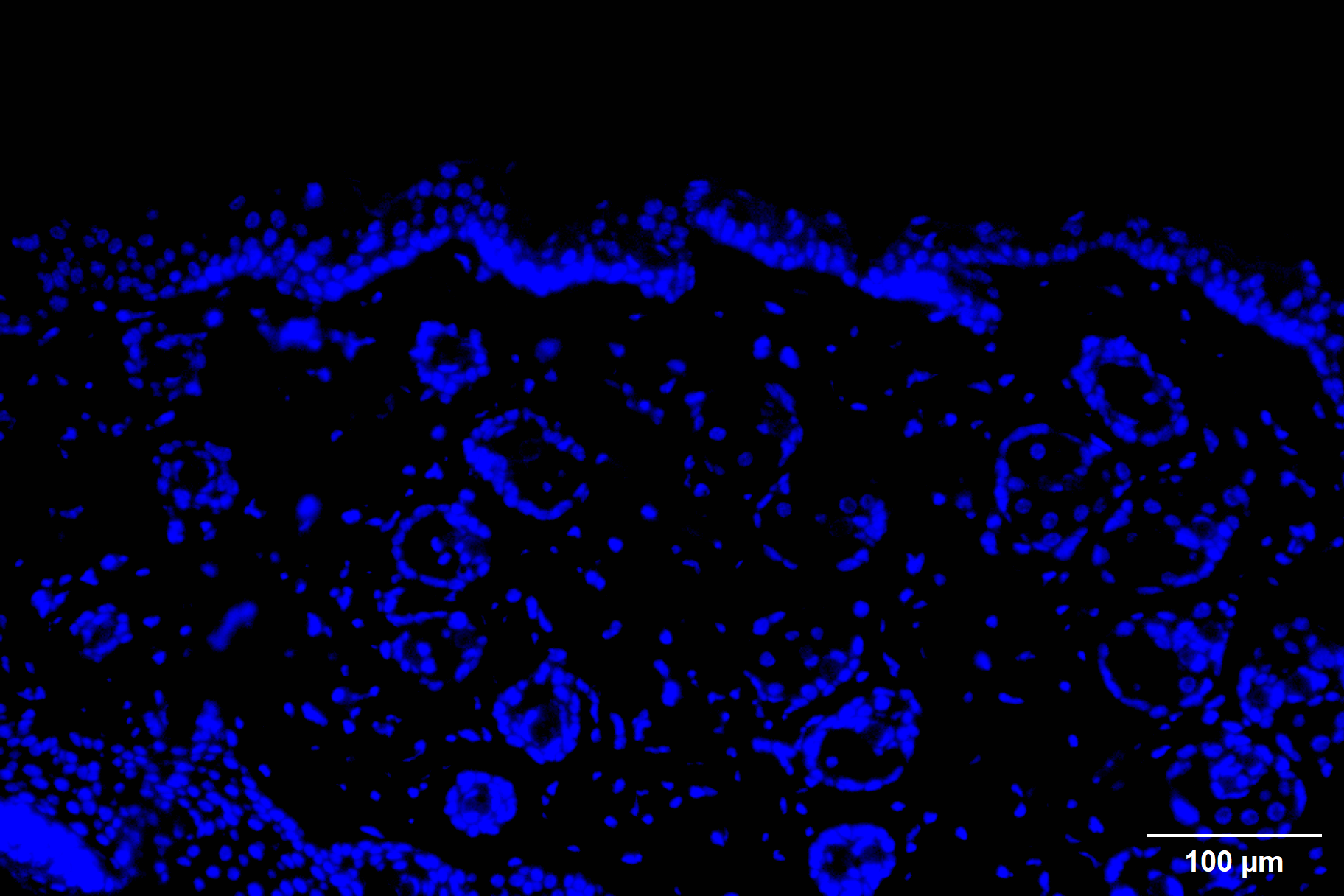

Supplement: Supplementary file 5 [file DataSheet5.zip › LA-Immunofluorescence staining image-Figure 5A/Figure 5A/7-1.tif]

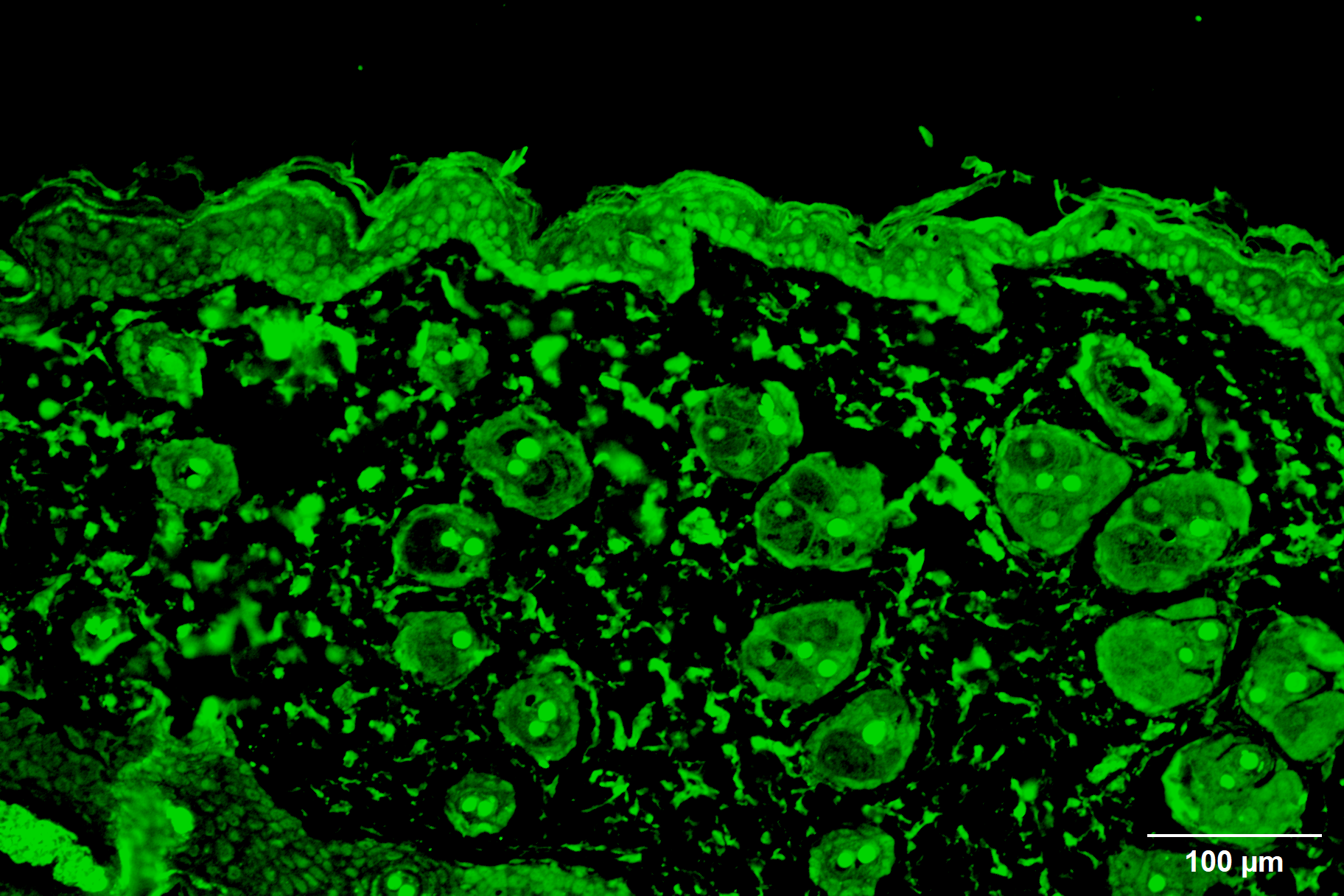

Supplement: Supplementary file 5 [file DataSheet5.zip › LA-Immunofluorescence staining image-Figure 5A/Figure 5A/7-2.tif]

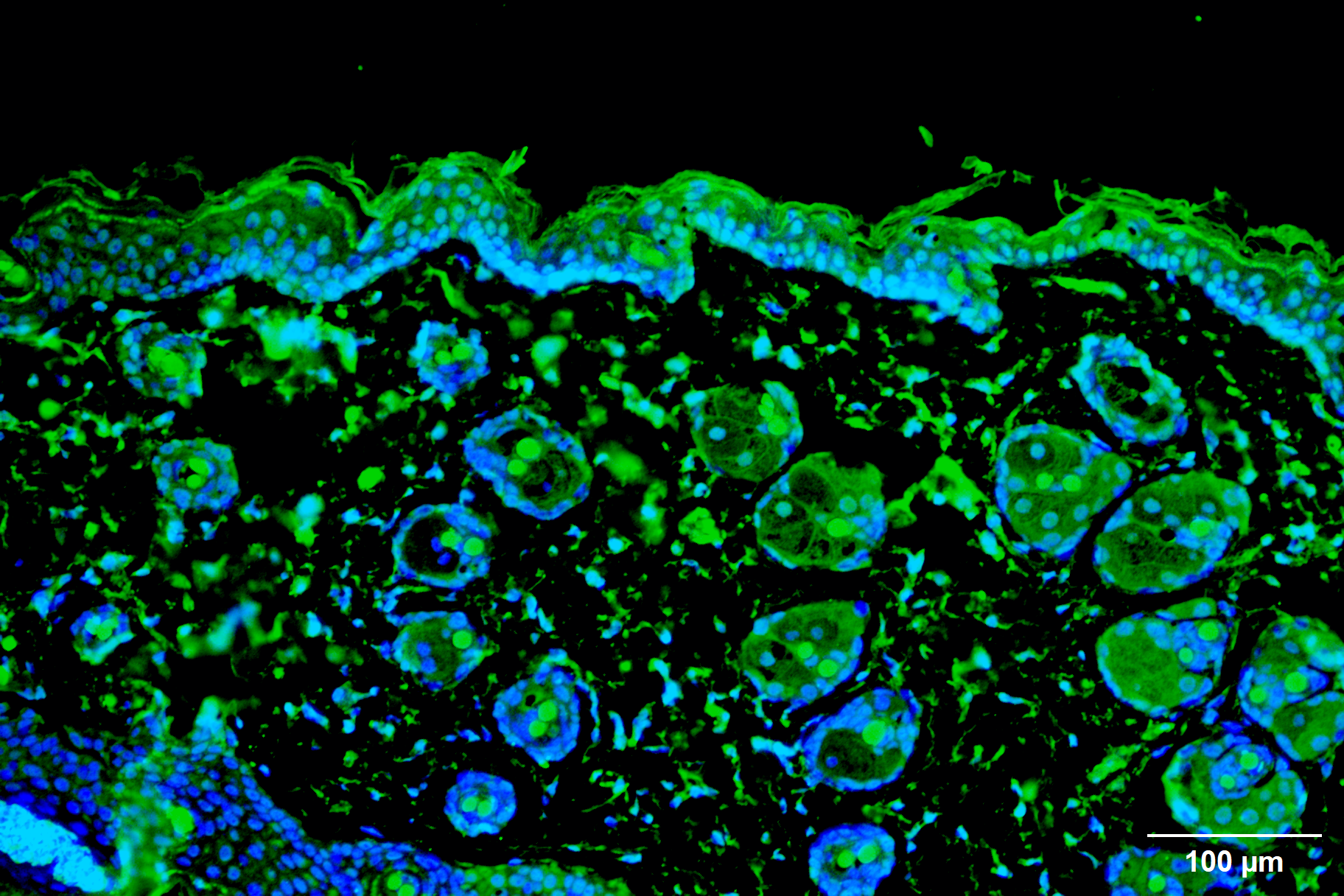

Supplement: Supplementary file 5 [file DataSheet5.zip › LA-Immunofluorescence staining image-Figure 5A/Figure 5A/7-3.tif]

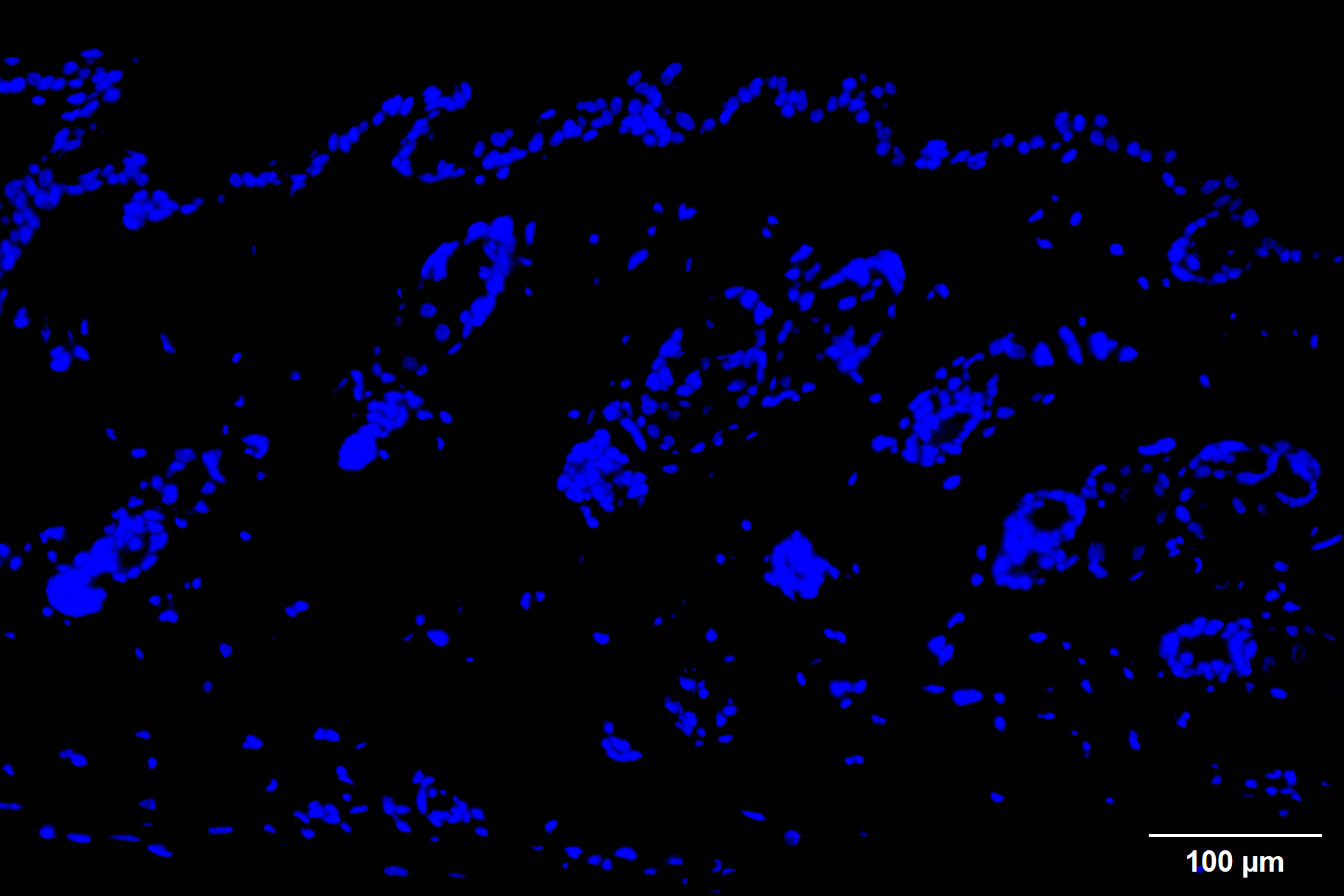

Supplement: Supplementary file 6 [file DataSheet6.zip › LA-Immunofluorescence staining image-Figure 5B/Figure 5B/1-1.tif]

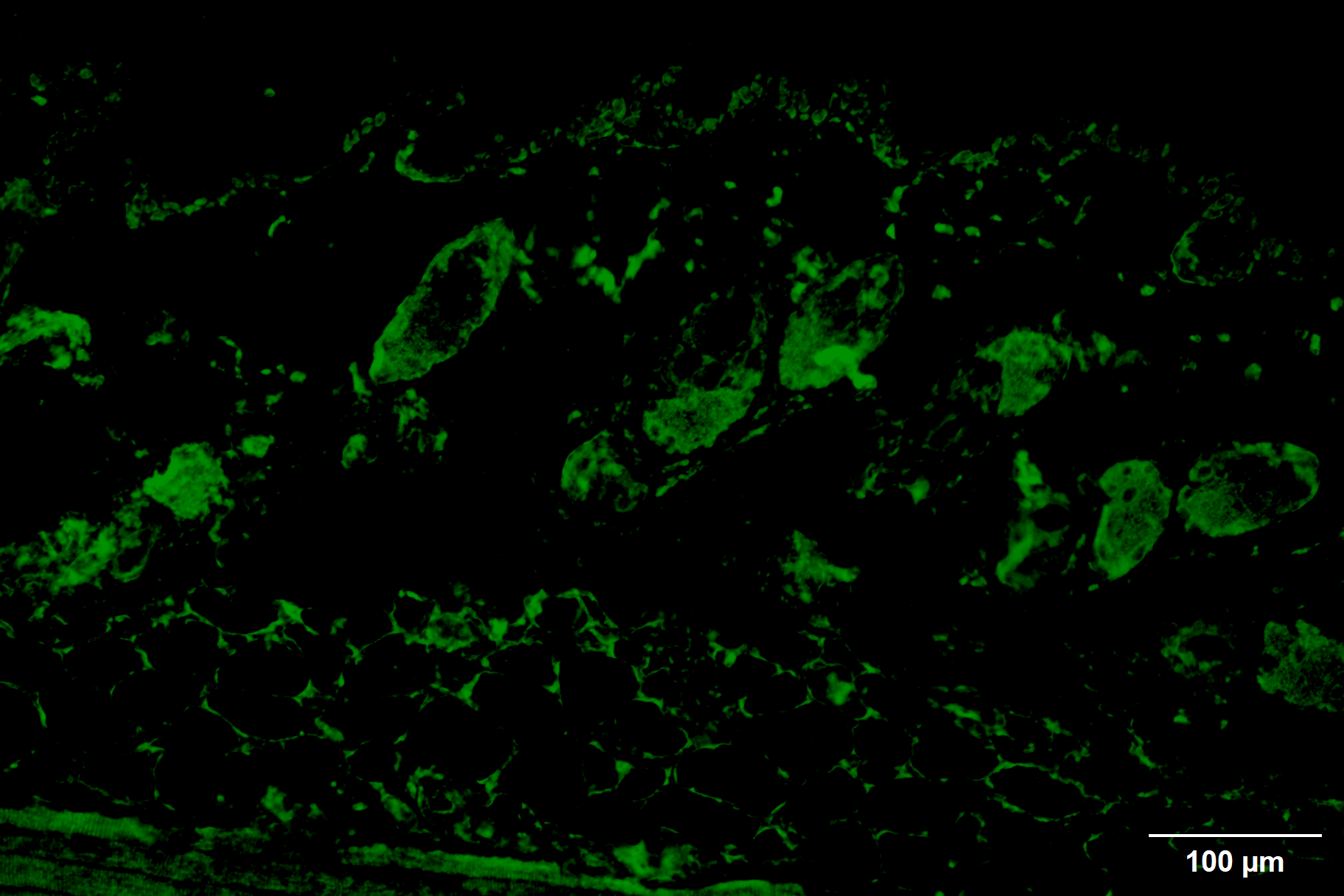

Supplement: Supplementary file 6 [file DataSheet6.zip › LA-Immunofluorescence staining image-Figure 5B/Figure 5B/1-2.tif]

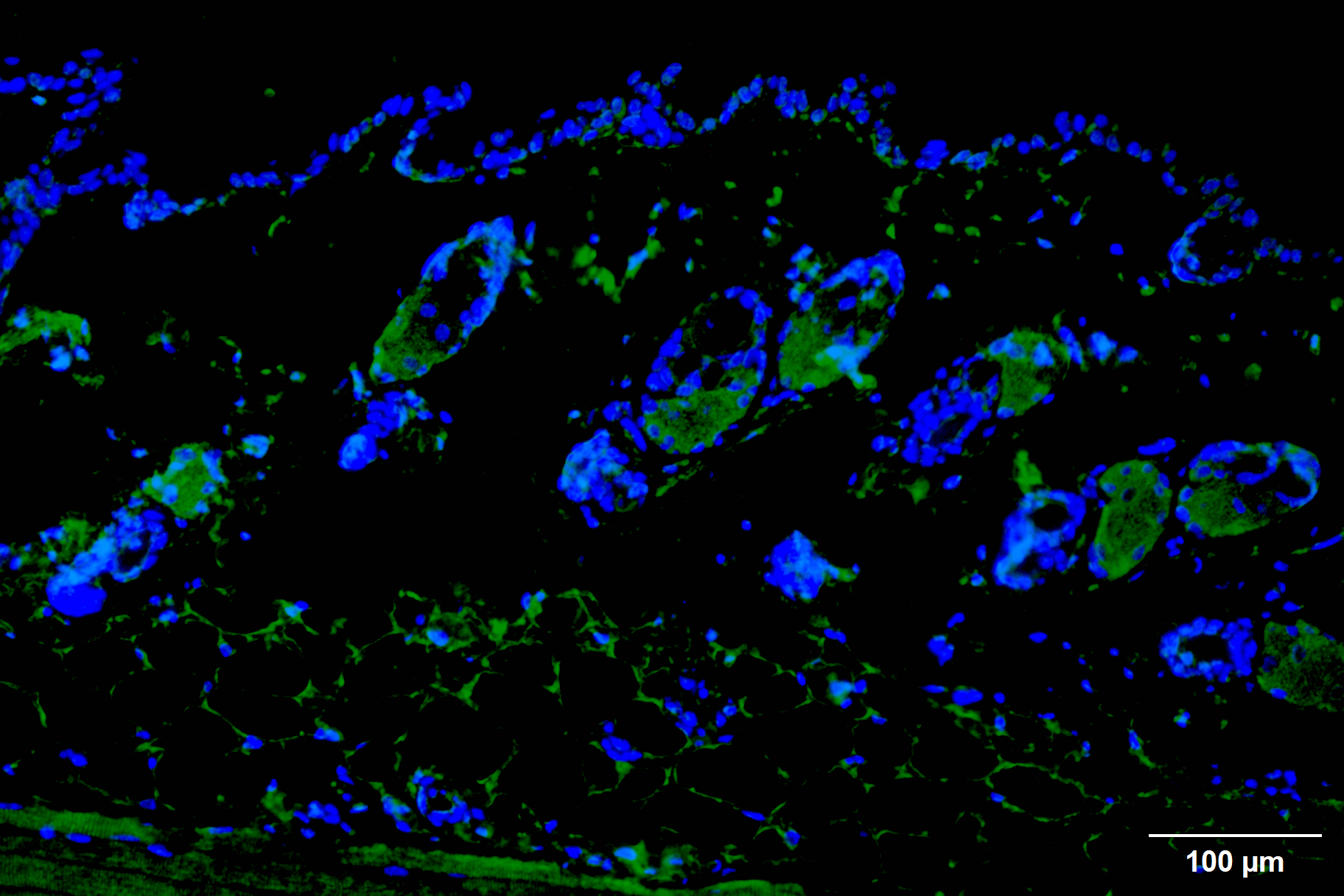

Supplement: Supplementary file 6 [file DataSheet6.zip › LA-Immunofluorescence staining image-Figure 5B/Figure 5B/1-3.tif]

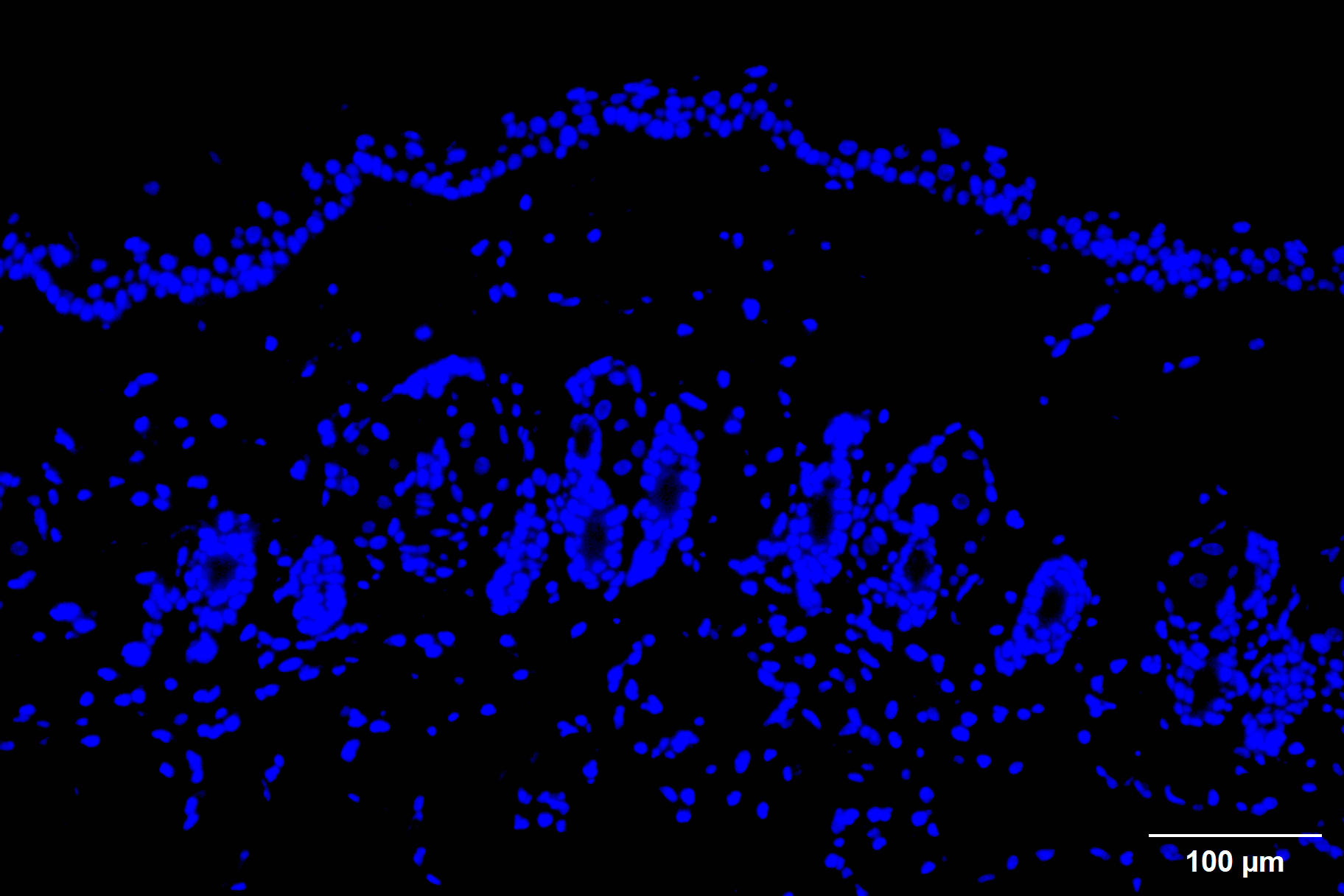

Supplement: Supplementary file 6 [file DataSheet6.zip › LA-Immunofluorescence staining image-Figure 5B/Figure 5B/2-1.tif]

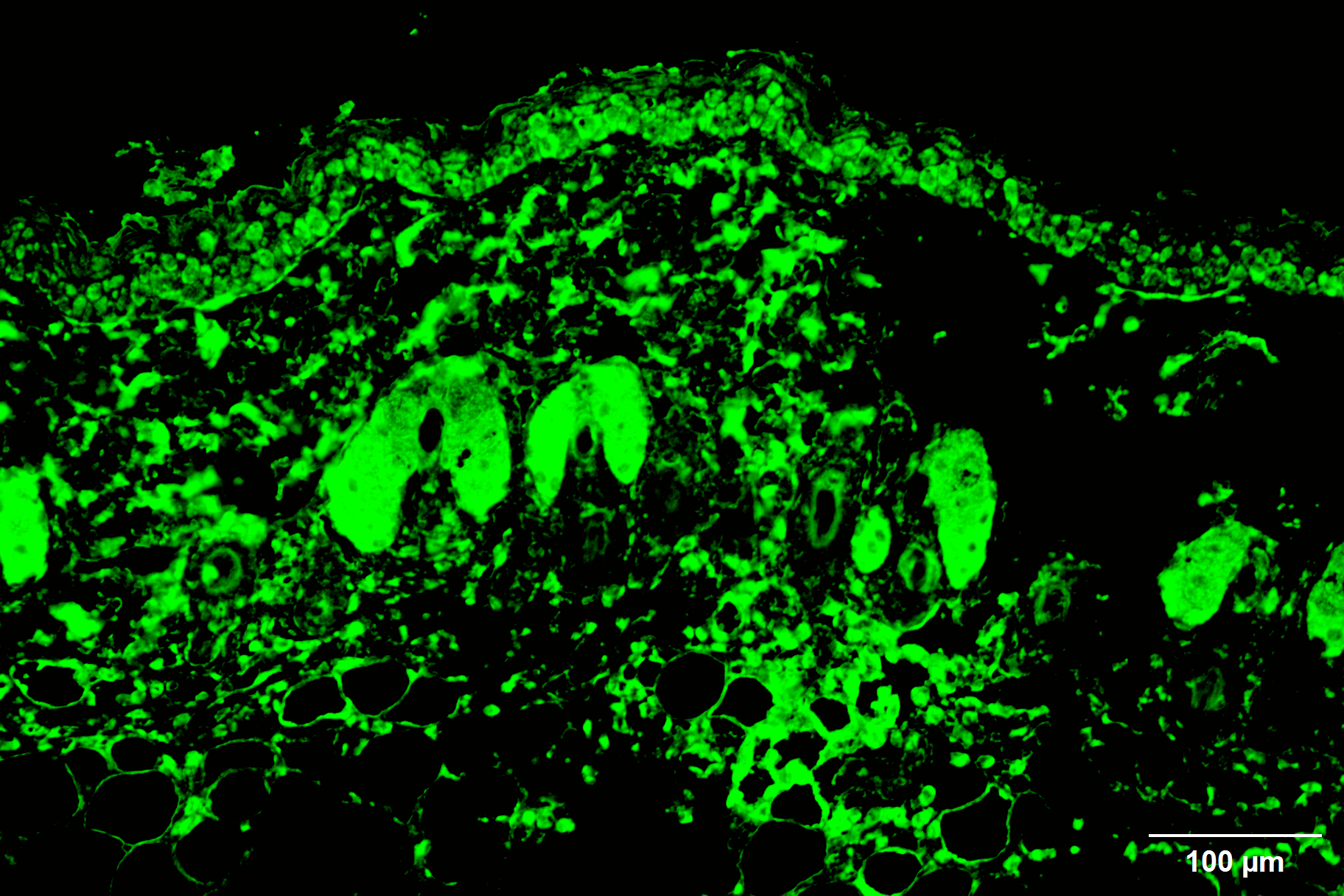

Supplement: Supplementary file 6 [file DataSheet6.zip › LA-Immunofluorescence staining image-Figure 5B/Figure 5B/2-2.tif]

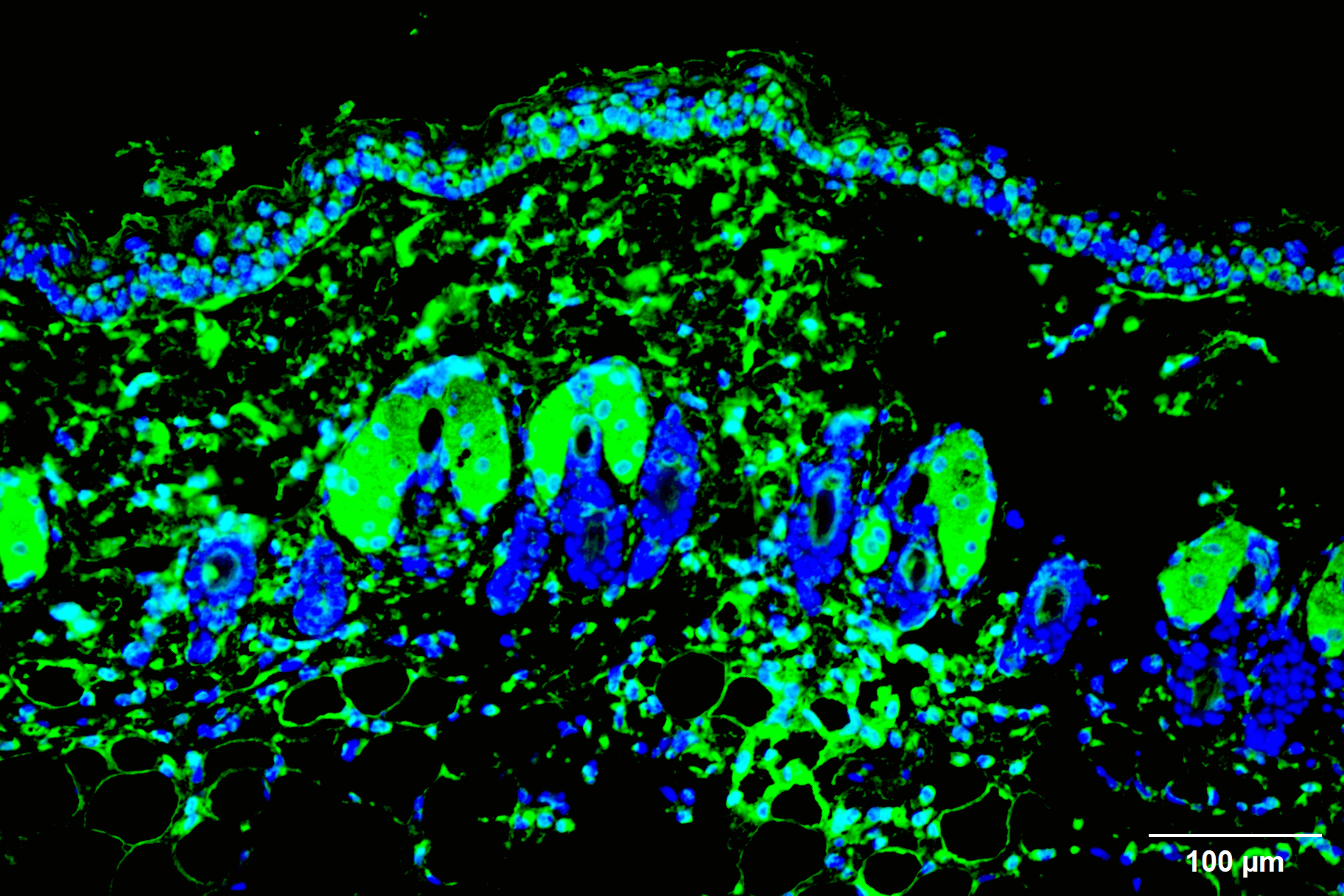

Supplement: Supplementary file 6 [file DataSheet6.zip › LA-Immunofluorescence staining image-Figure 5B/Figure 5B/2-3.tif]

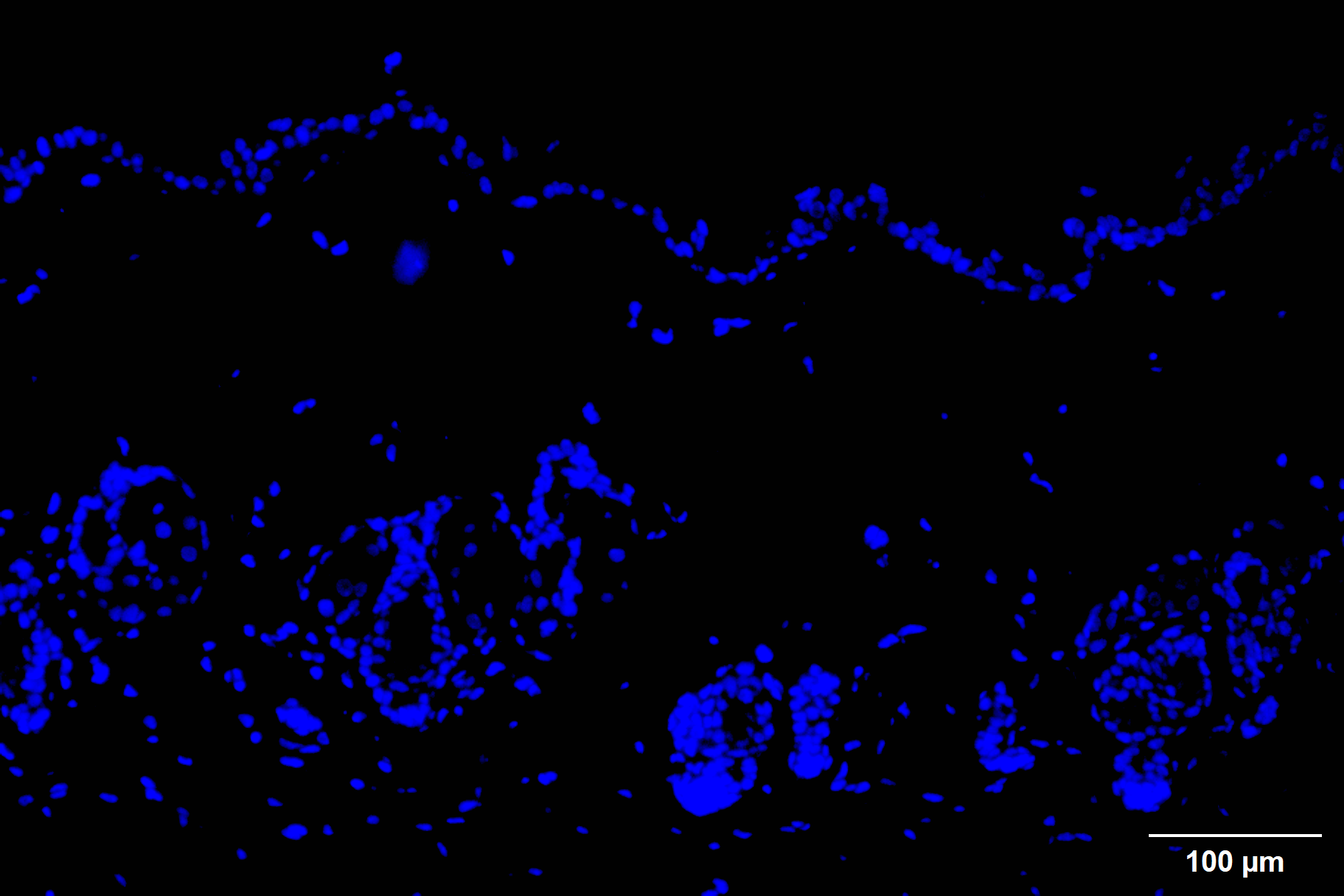

Supplement: Supplementary file 6 [file DataSheet6.zip › LA-Immunofluorescence staining image-Figure 5B/Figure 5B/3-1.tif]

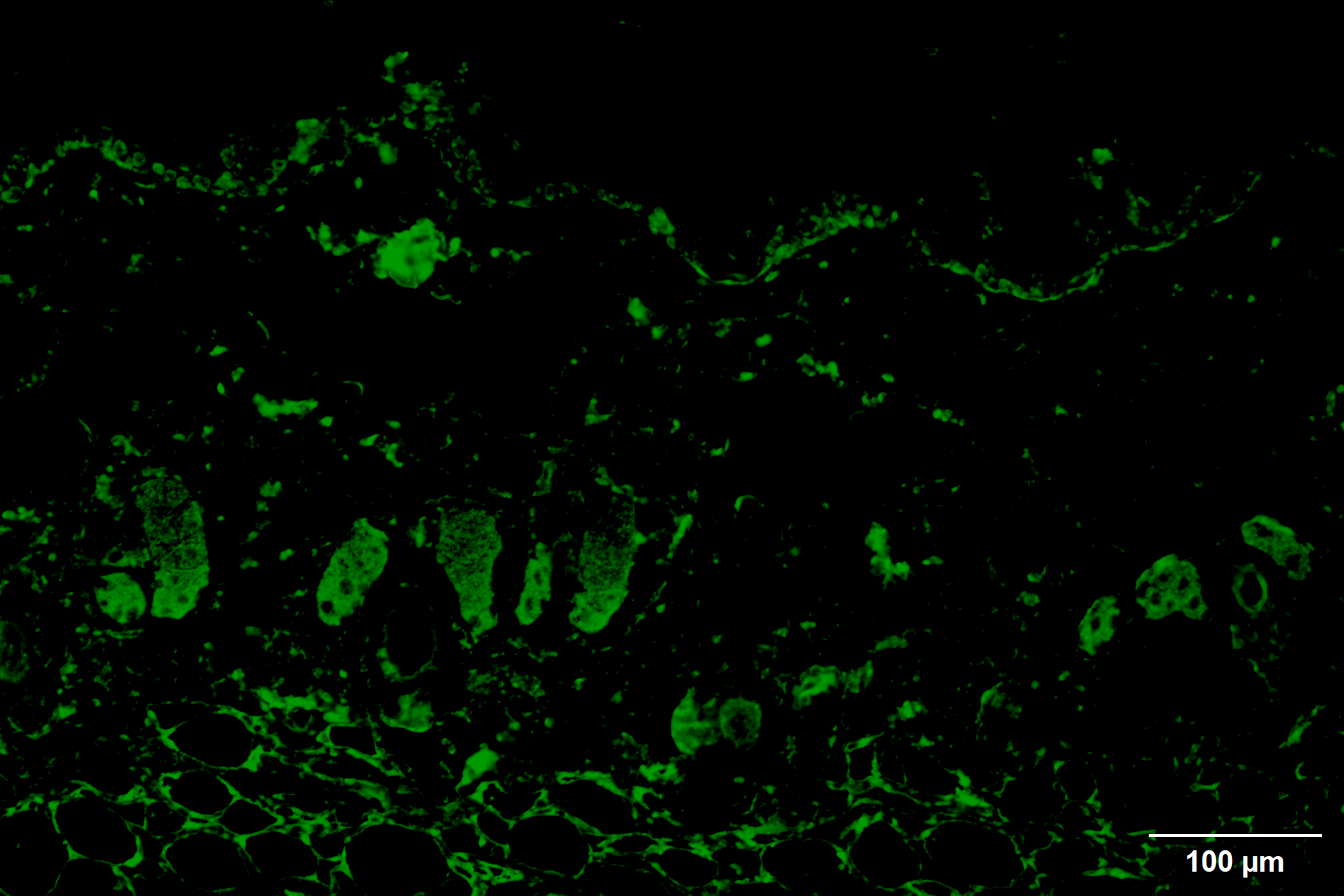

Supplement: Supplementary file 6 [file DataSheet6.zip › LA-Immunofluorescence staining image-Figure 5B/Figure 5B/3-2.tif]

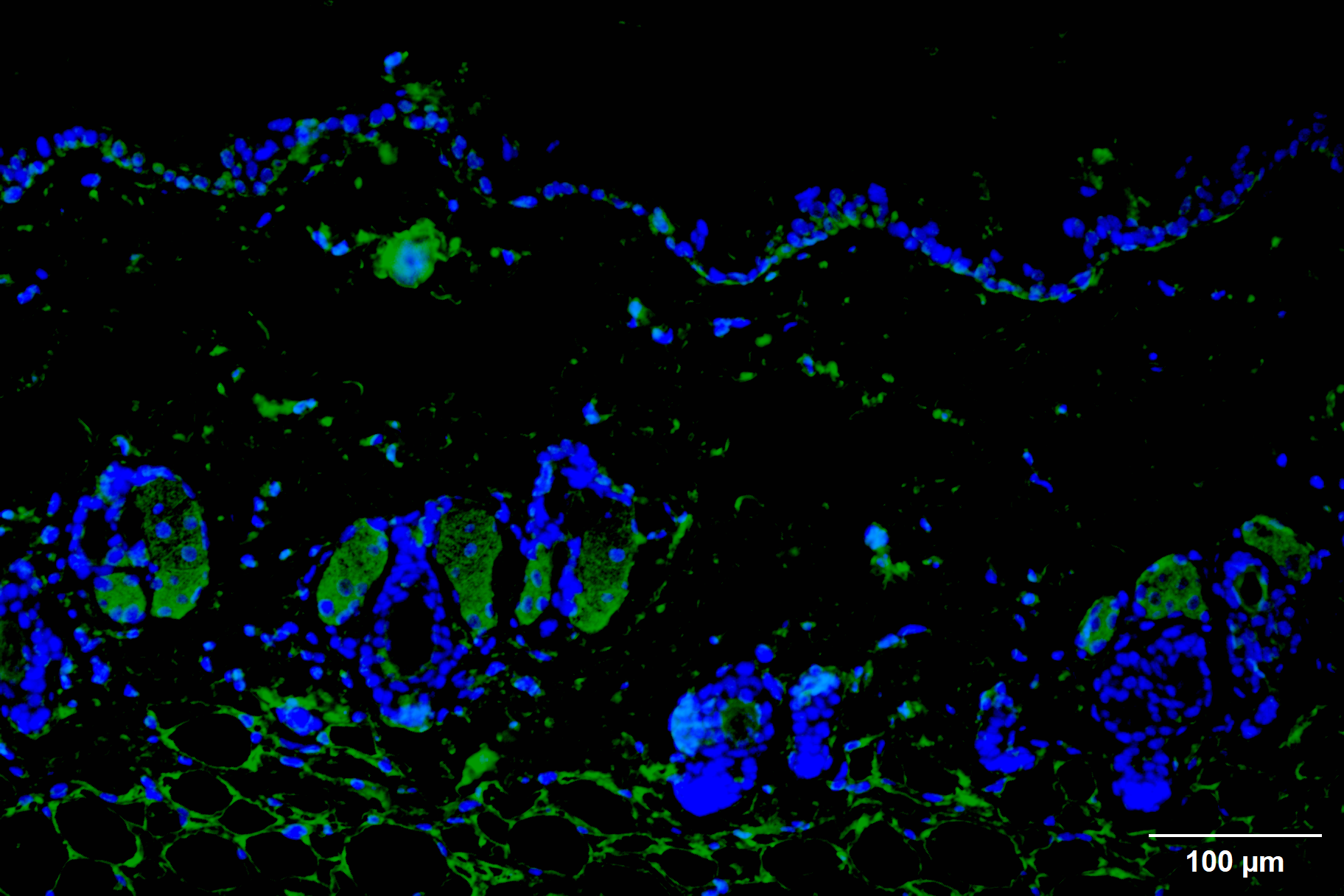

Supplement: Supplementary file 6 [file DataSheet6.zip › LA-Immunofluorescence staining image-Figure 5B/Figure 5B/3-3.tif]

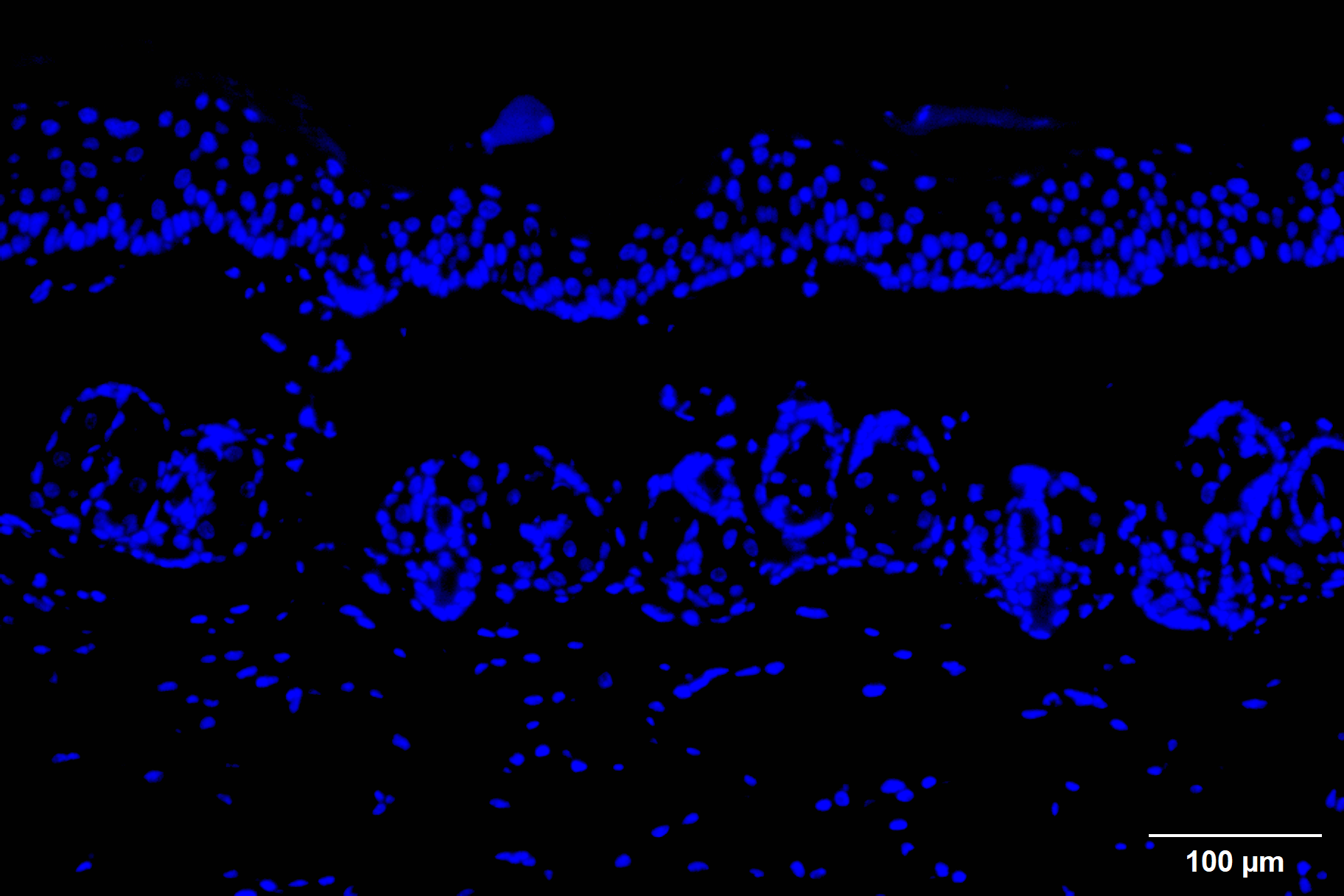

Supplement: Supplementary file 6 [file DataSheet6.zip › LA-Immunofluorescence staining image-Figure 5B/Figure 5B/4-1.tif]

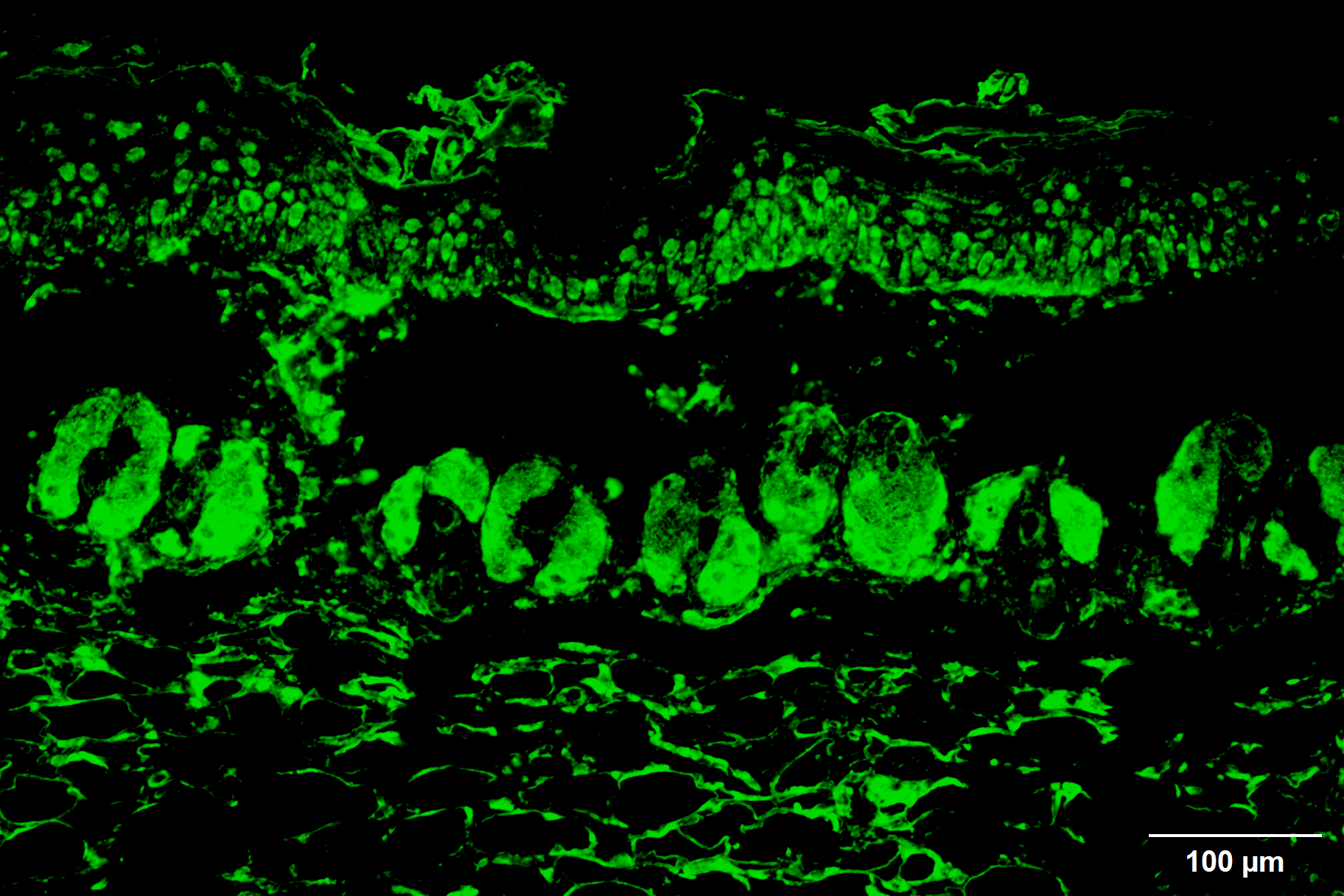

Supplement: Supplementary file 6 [file DataSheet6.zip › LA-Immunofluorescence staining image-Figure 5B/Figure 5B/4-2.tif]

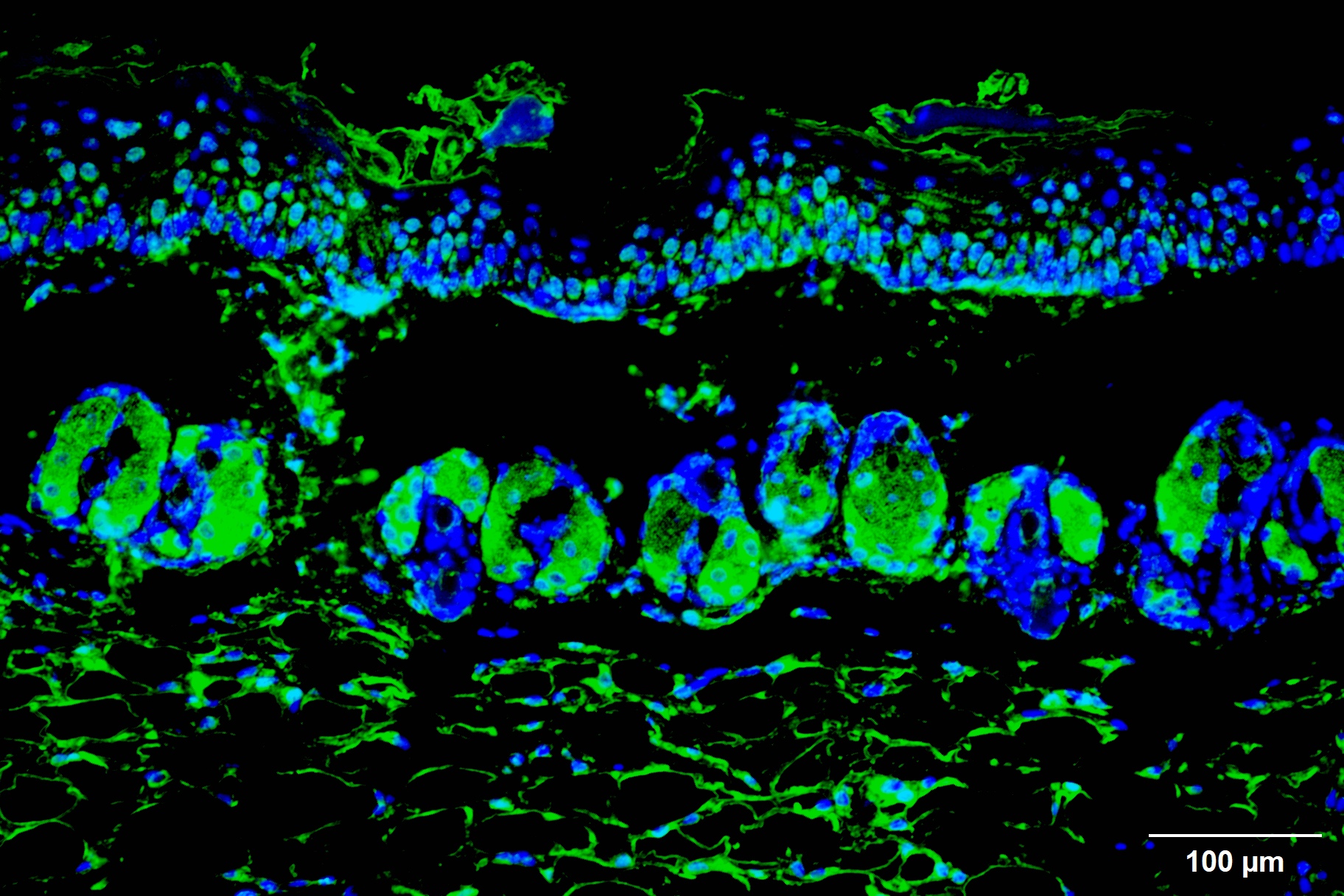

Supplement: Supplementary file 6 [file DataSheet6.zip › LA-Immunofluorescence staining image-Figure 5B/Figure 5B/4-3.tif]

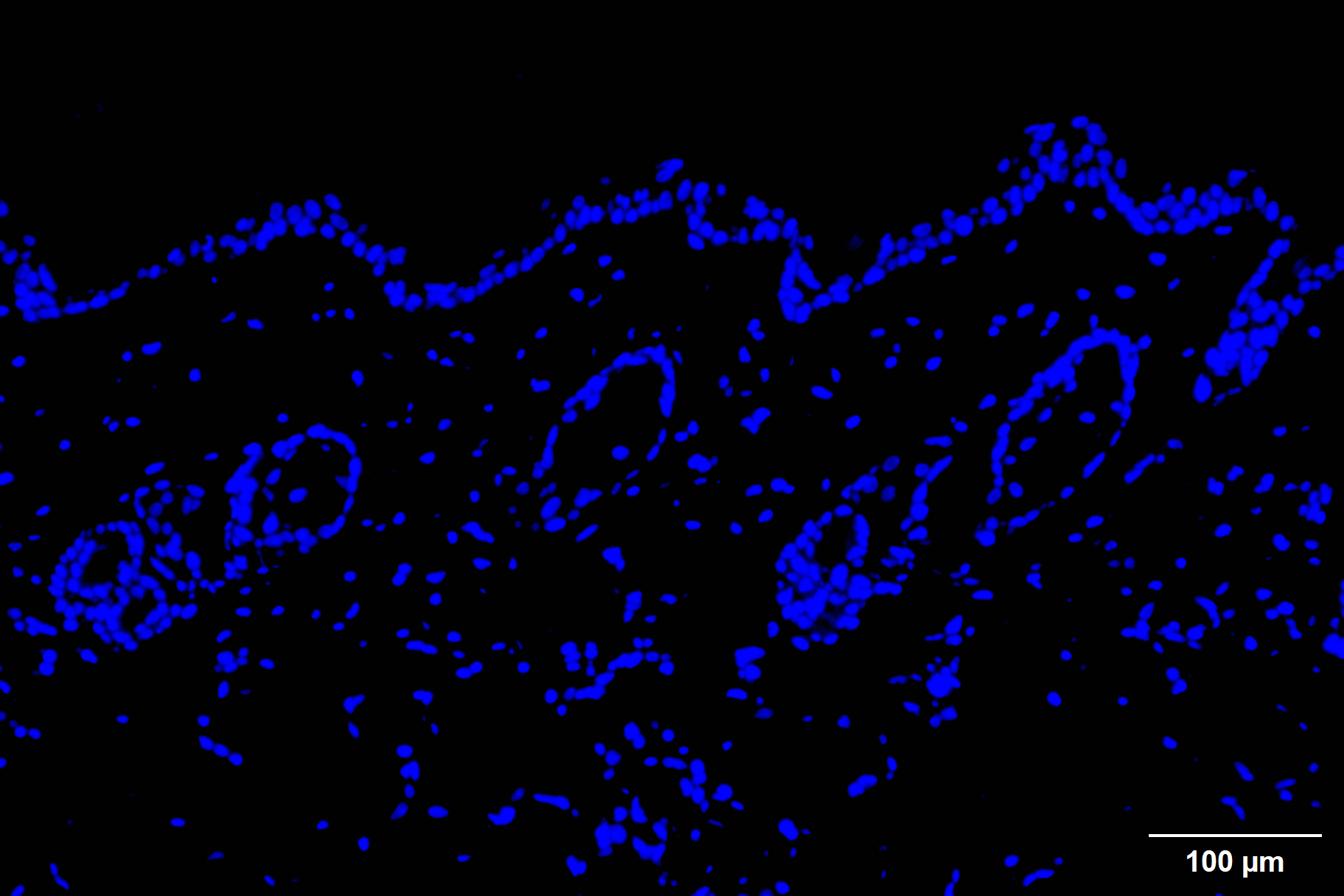

Supplement: Supplementary file 6 [file DataSheet6.zip › LA-Immunofluorescence staining image-Figure 5B/Figure 5B/5-1.tif]

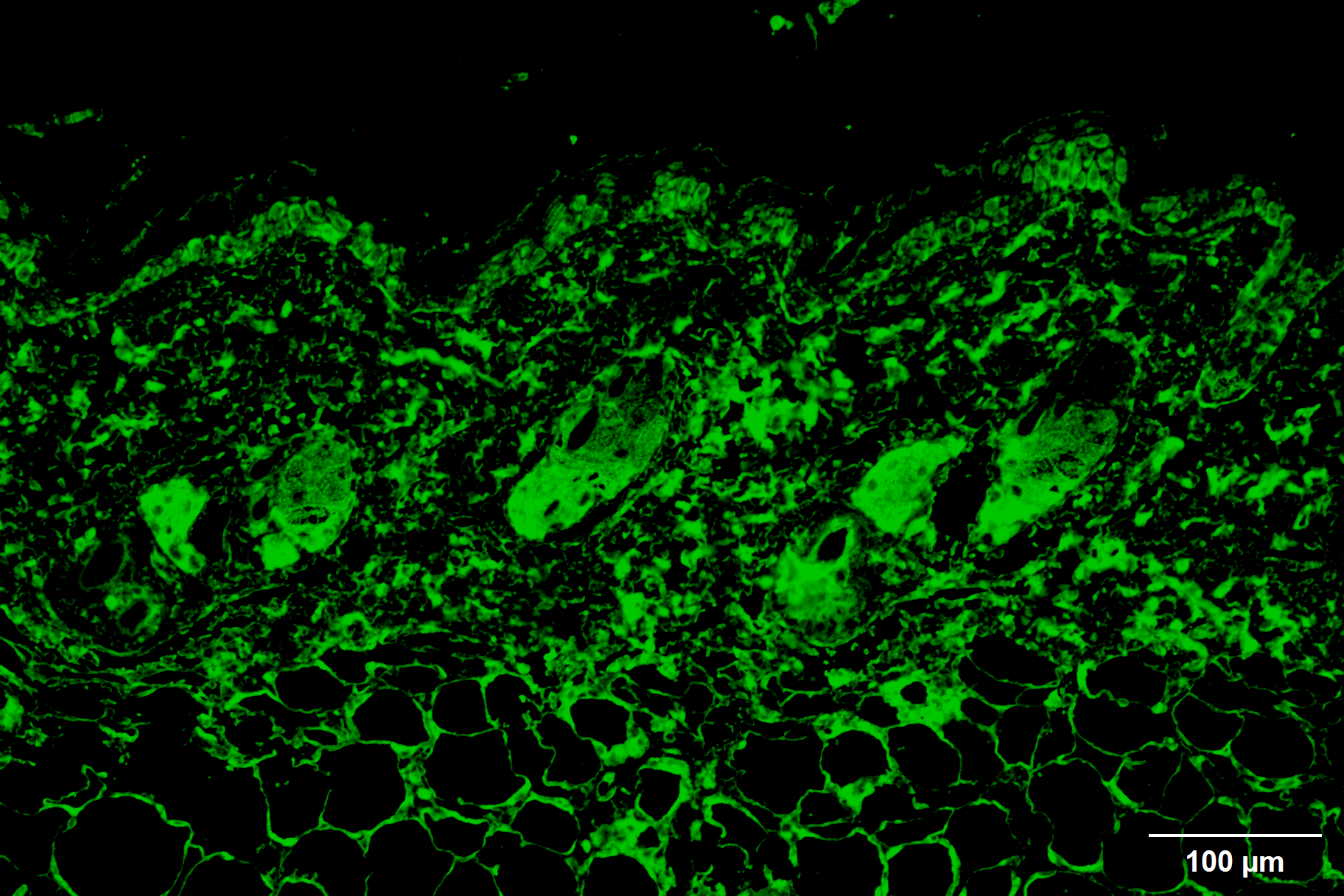

Supplement: Supplementary file 6 [file DataSheet6.zip › LA-Immunofluorescence staining image-Figure 5B/Figure 5B/5-2.tif]

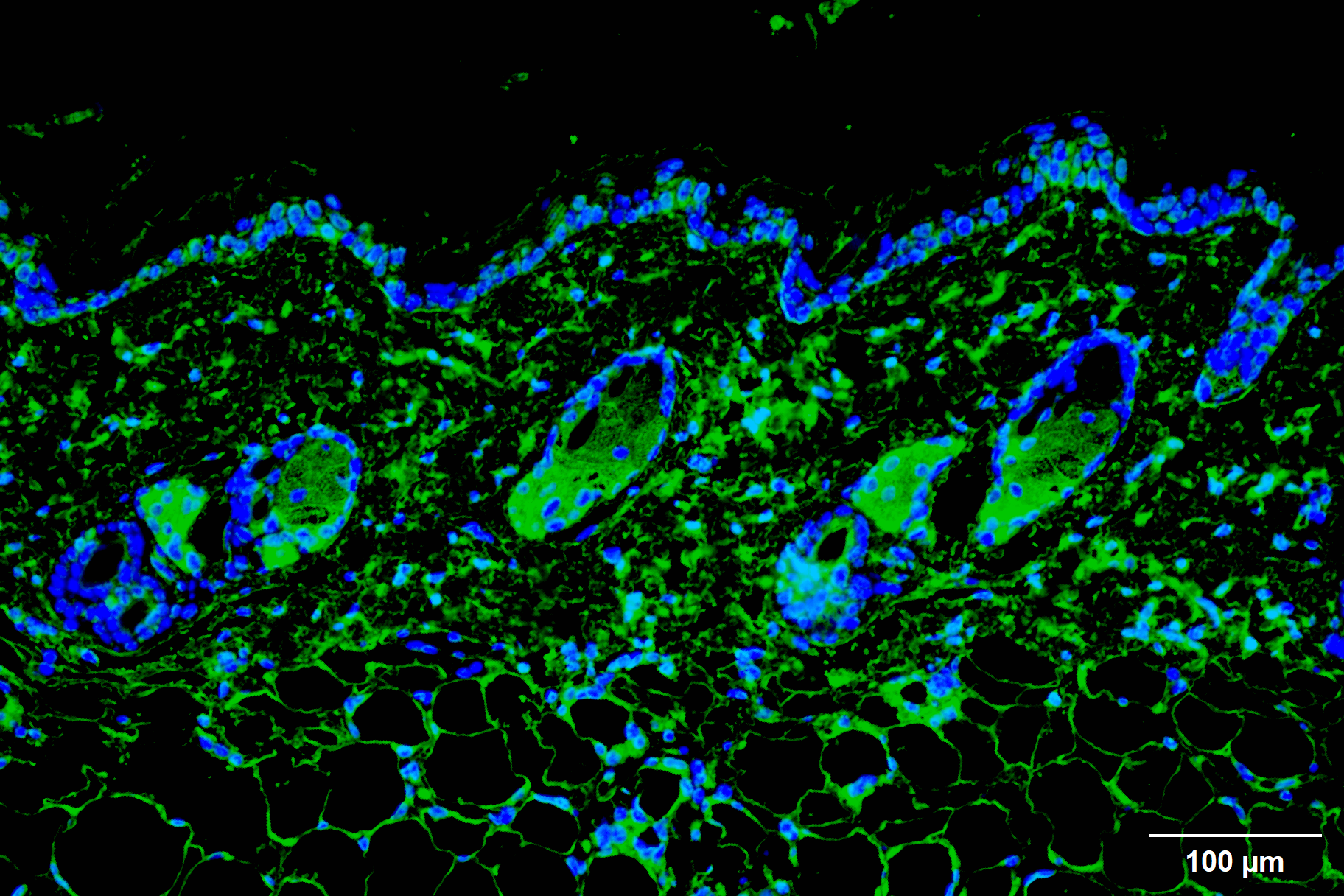

Supplement: Supplementary file 6 [file DataSheet6.zip › LA-Immunofluorescence staining image-Figure 5B/Figure 5B/5-3.tif]

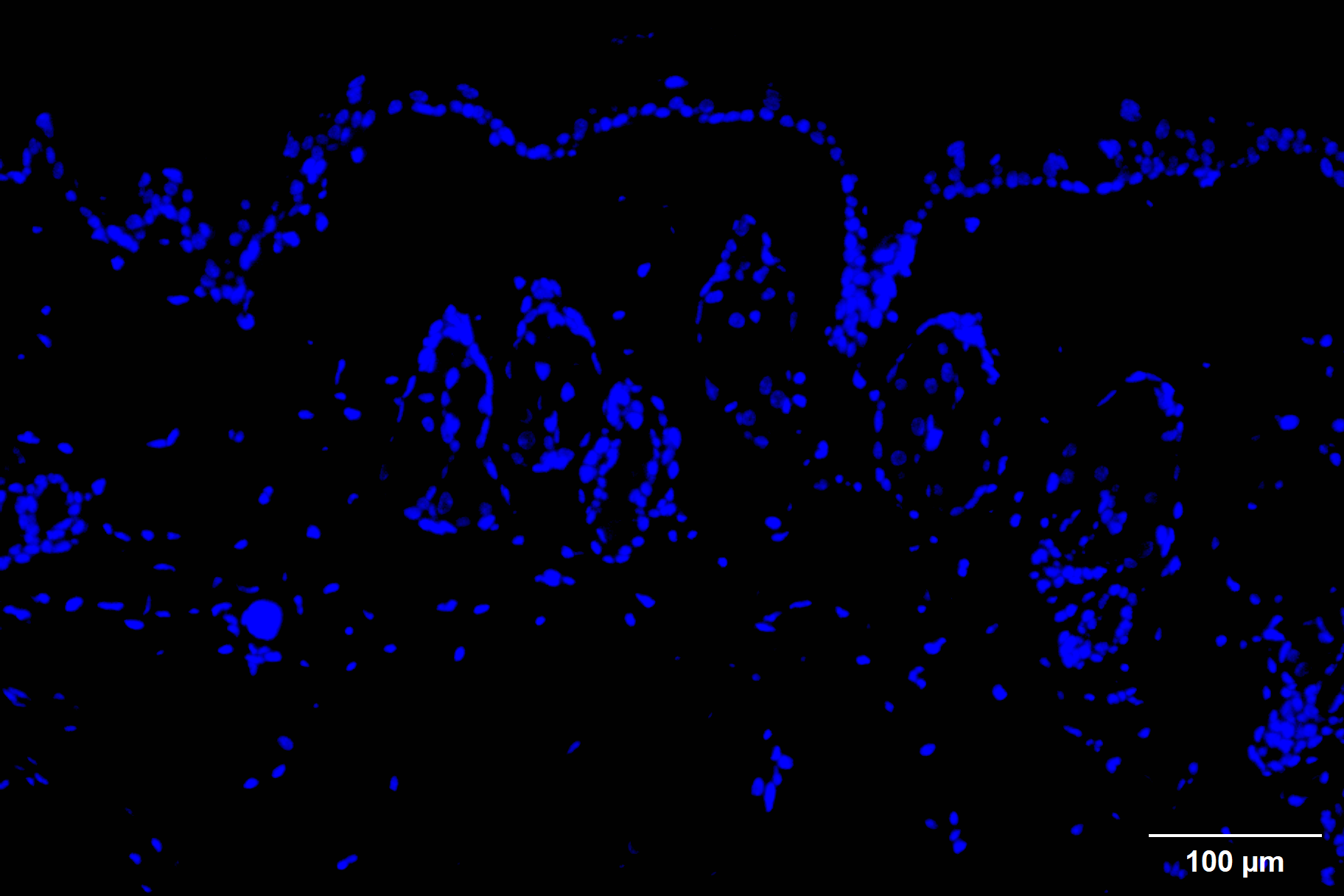

Supplement: Supplementary file 6 [file DataSheet6.zip › LA-Immunofluorescence staining image-Figure 5B/Figure 5B/6-1.tif]

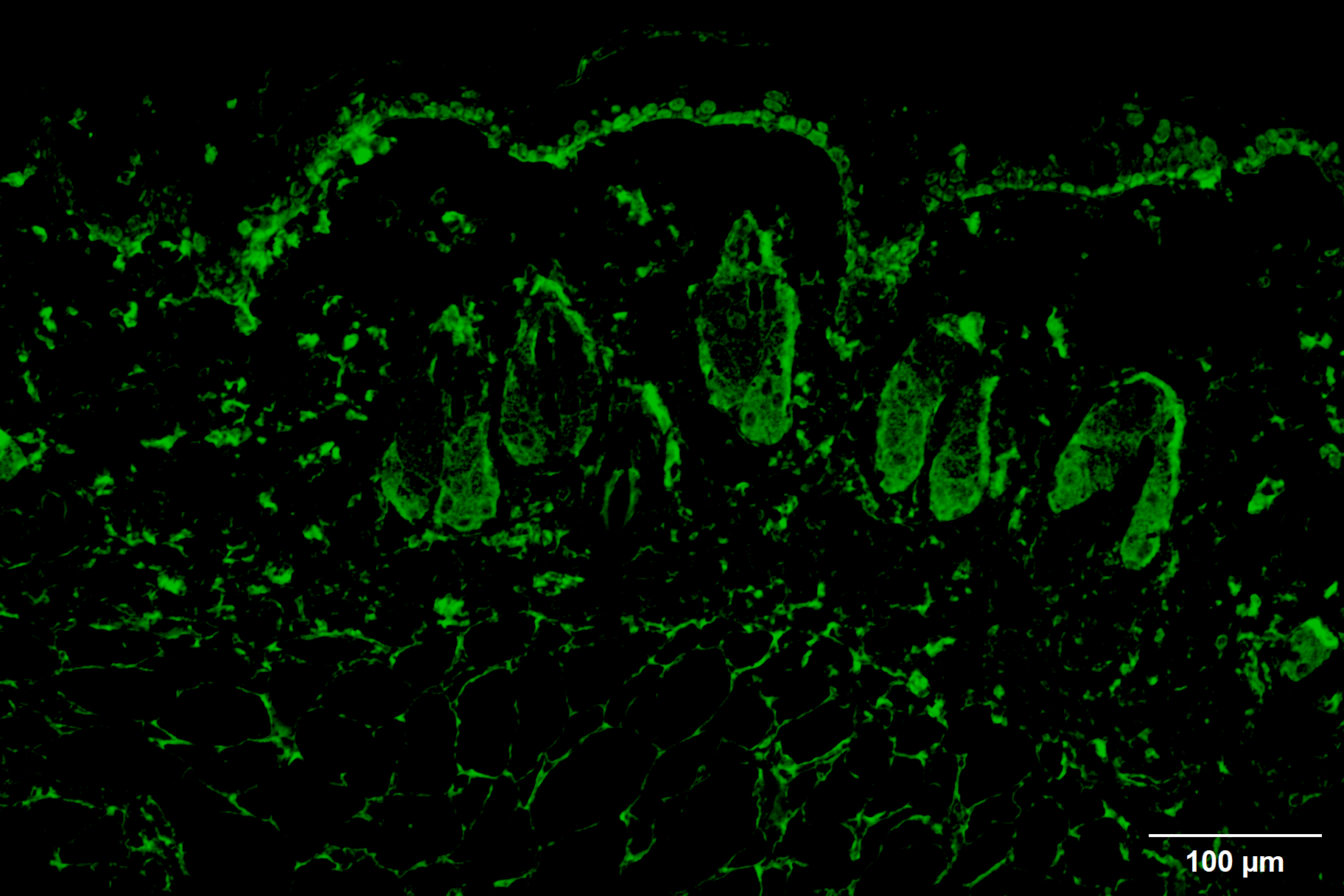

Supplement: Supplementary file 6 [file DataSheet6.zip › LA-Immunofluorescence staining image-Figure 5B/Figure 5B/6-2.tif]

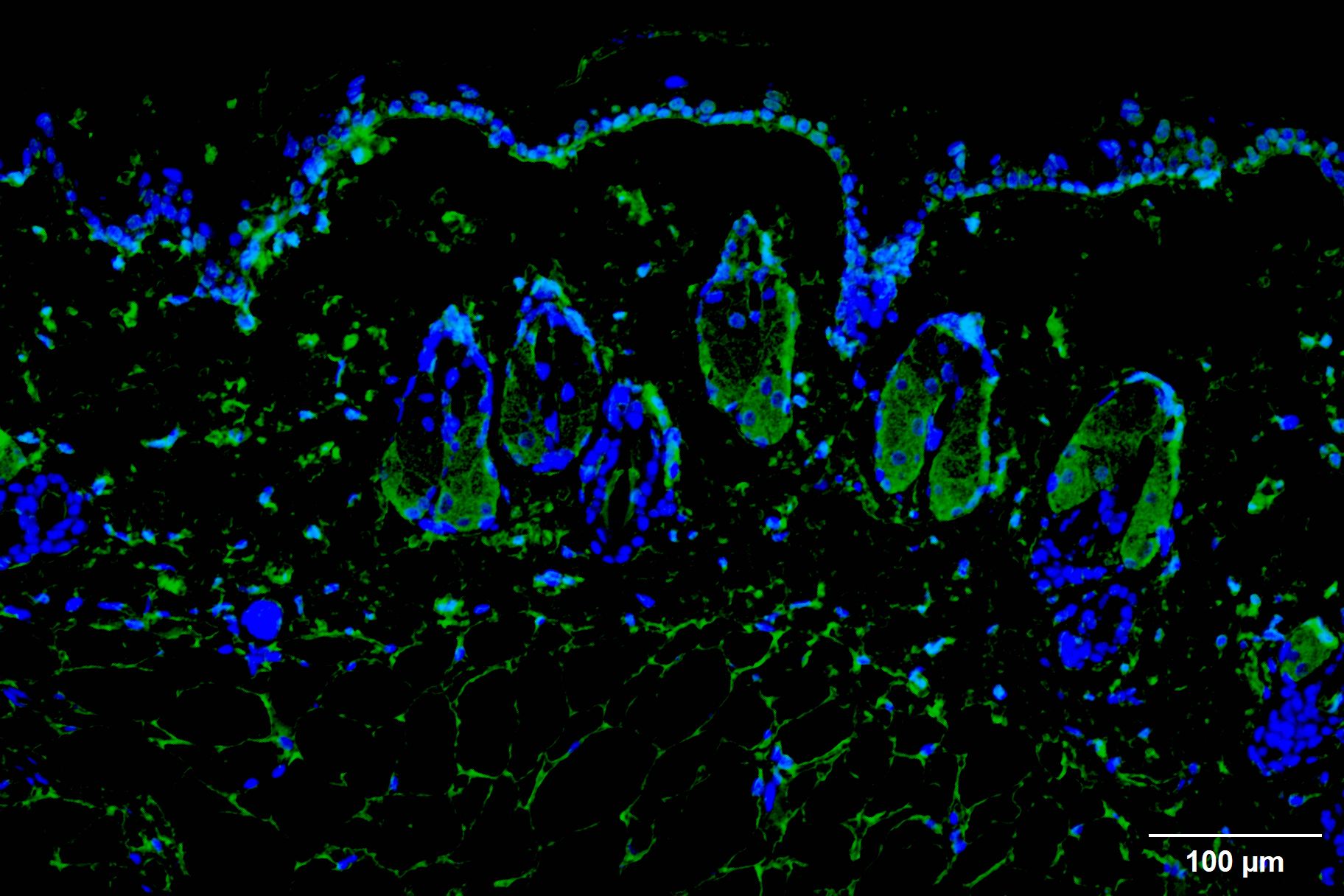

Supplement: Supplementary file 6 [file DataSheet6.zip › LA-Immunofluorescence staining image-Figure 5B/Figure 5B/6-3.tif]

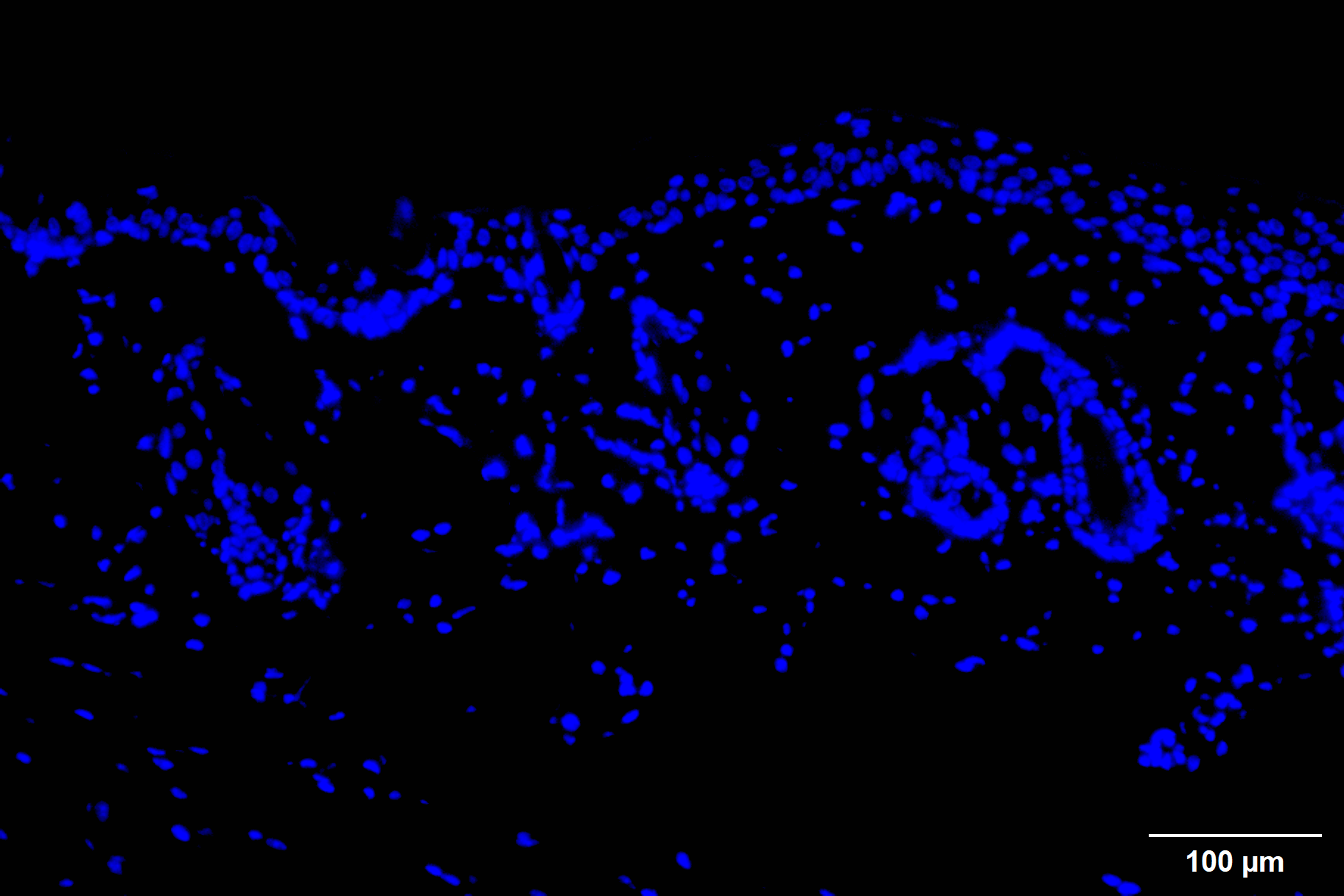

Supplement: Supplementary file 6 [file DataSheet6.zip › LA-Immunofluorescence staining image-Figure 5B/Figure 5B/7-1.tif]

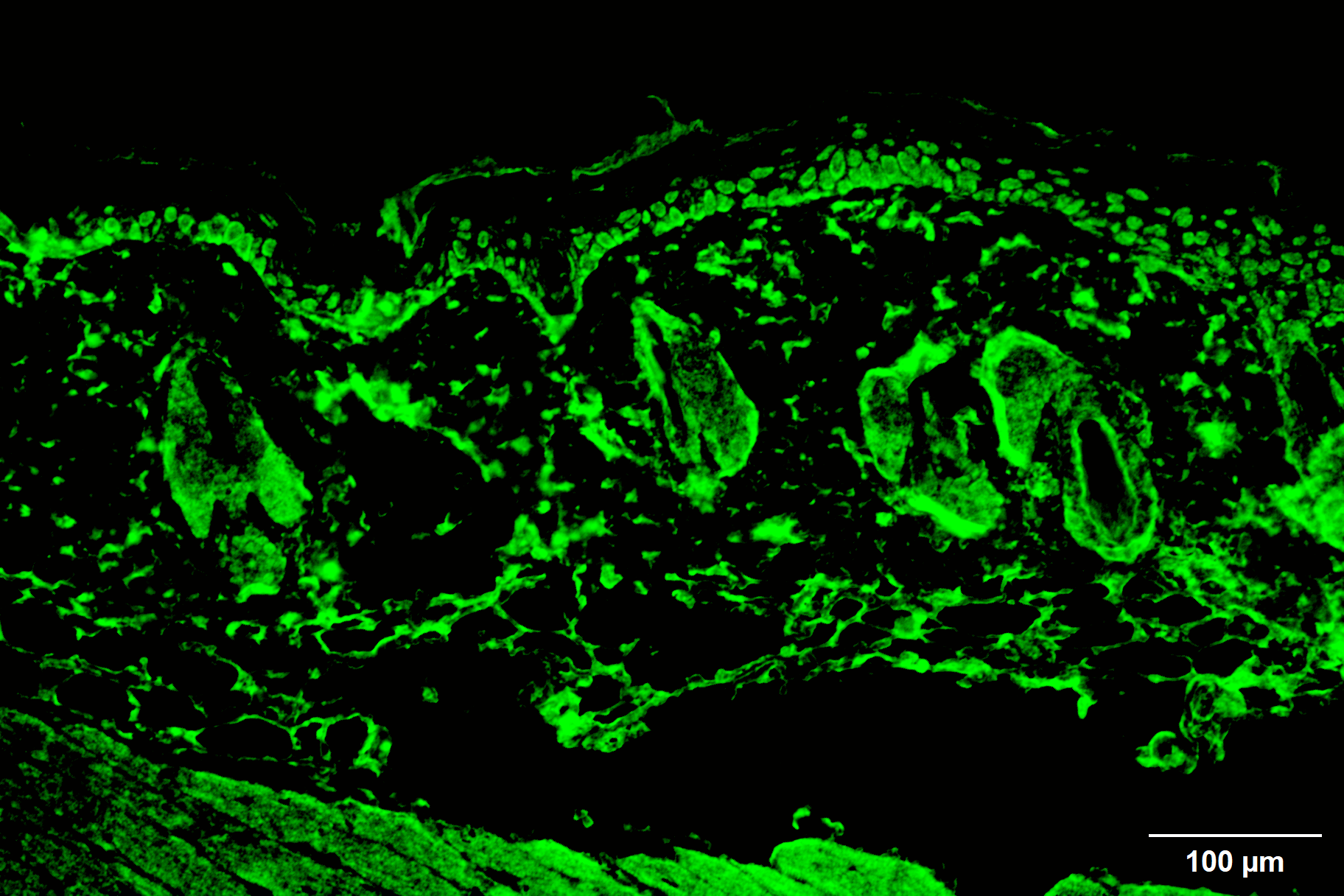

Supplement: Supplementary file 6 [file DataSheet6.zip › LA-Immunofluorescence staining image-Figure 5B/Figure 5B/7-2.tif]

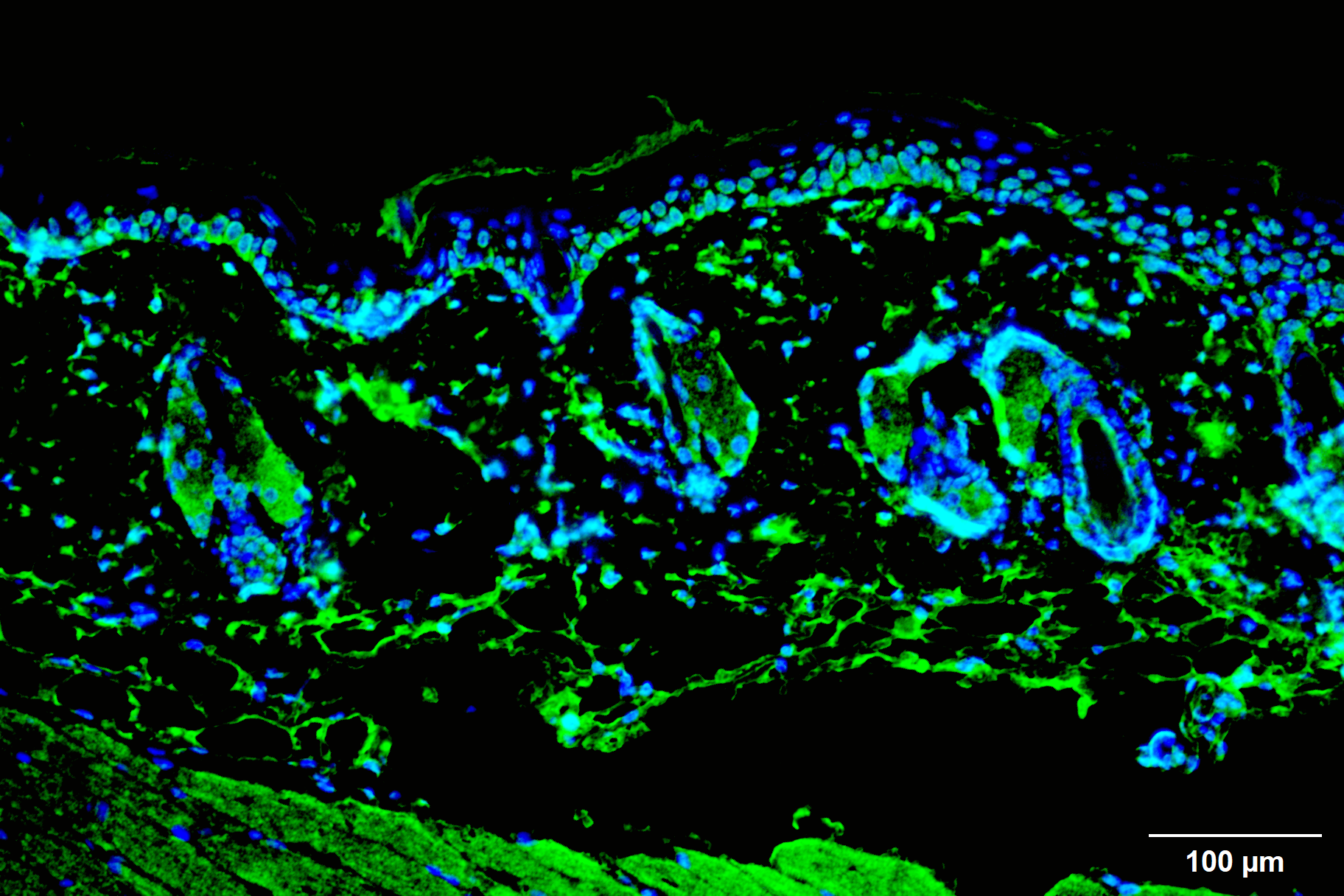

Supplement: Supplementary file 6 [file DataSheet6.zip › LA-Immunofluorescence staining image-Figure 5B/Figure 5B/7-3.tif]

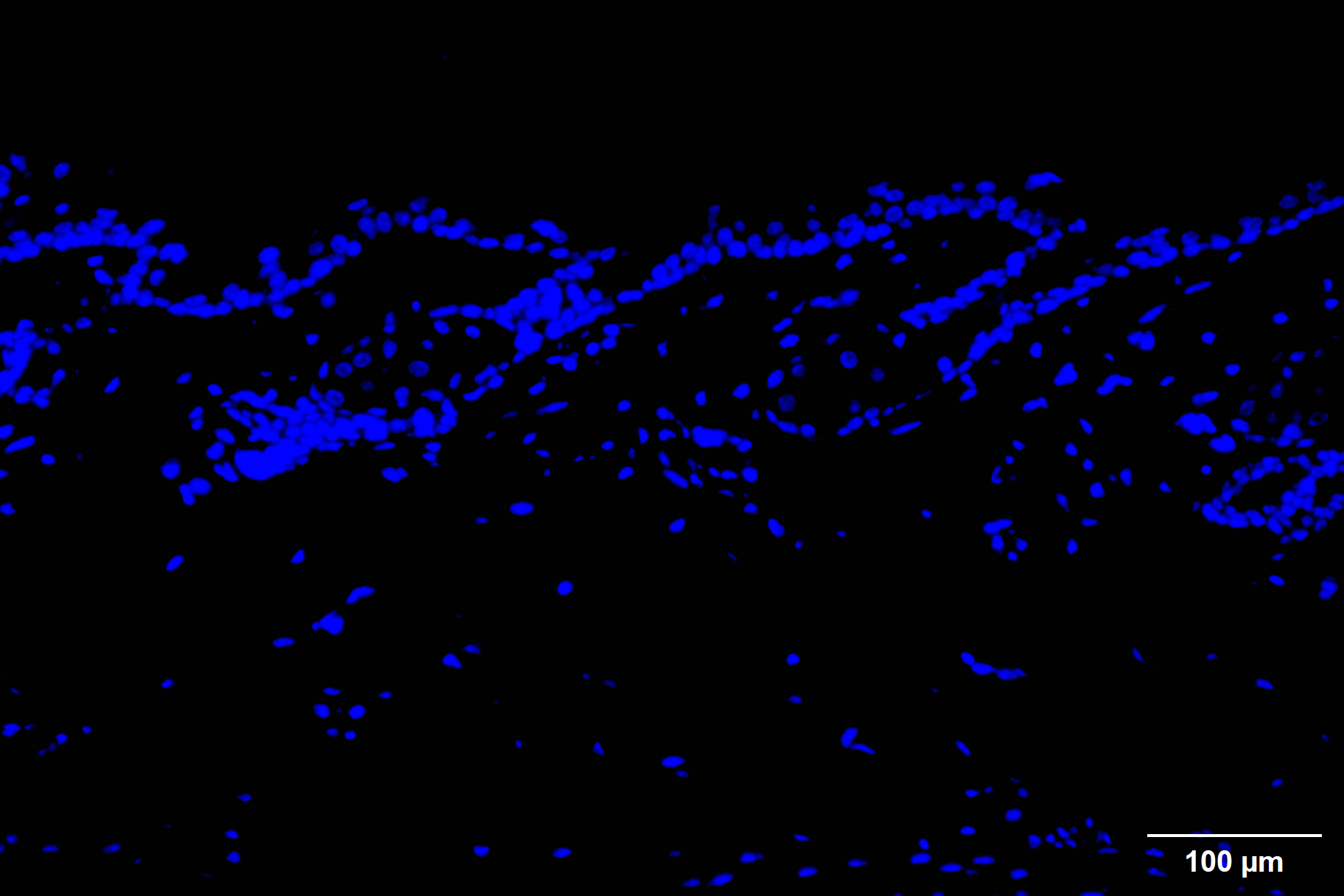

Supplement: Supplementary file 7 [file DataSheet7.zip › LA-Immunofluorescence staining image-Figure 5C/Figure 5C/1-1.tif]

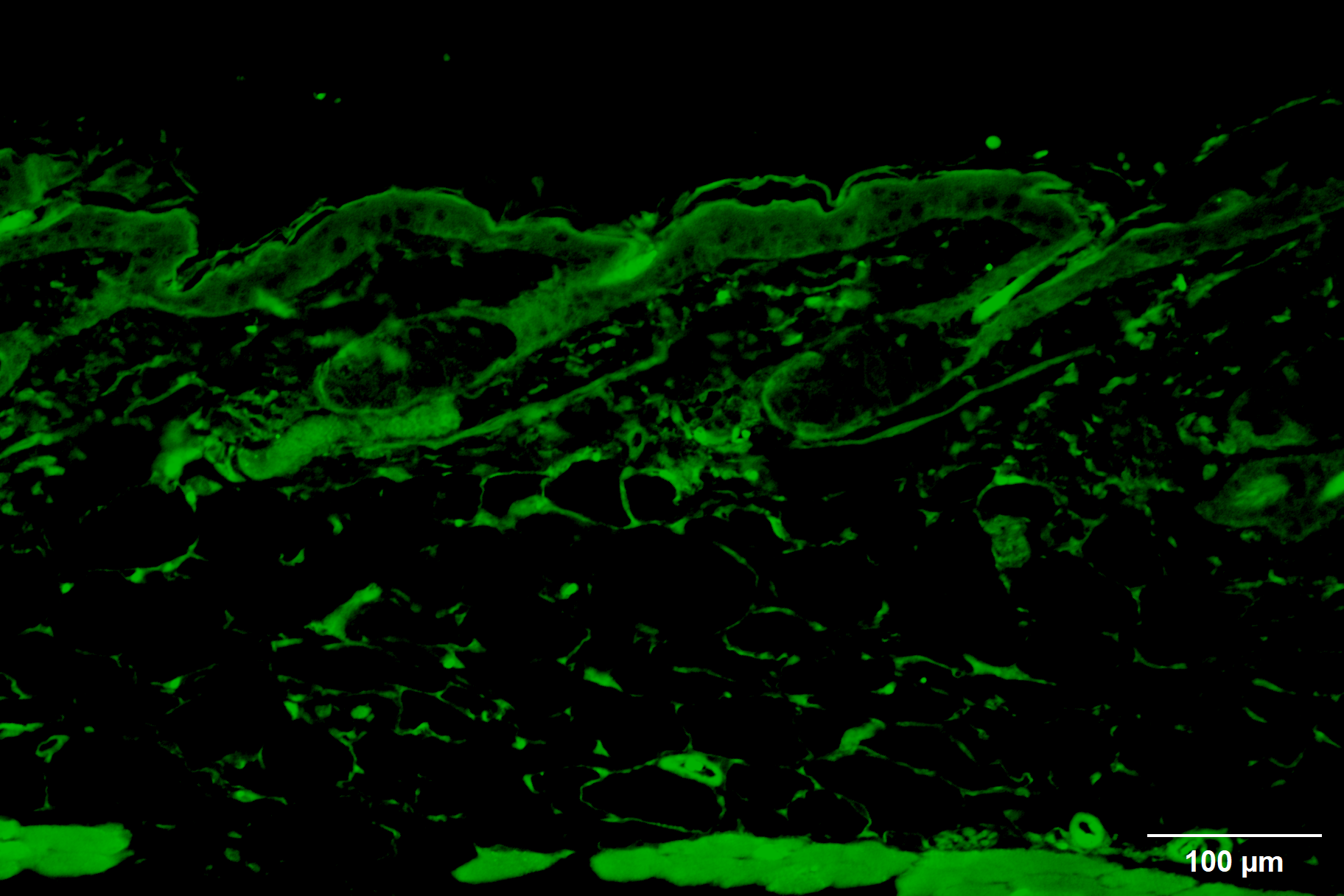

Supplement: Supplementary file 7 [file DataSheet7.zip › LA-Immunofluorescence staining image-Figure 5C/Figure 5C/1-2.tif]

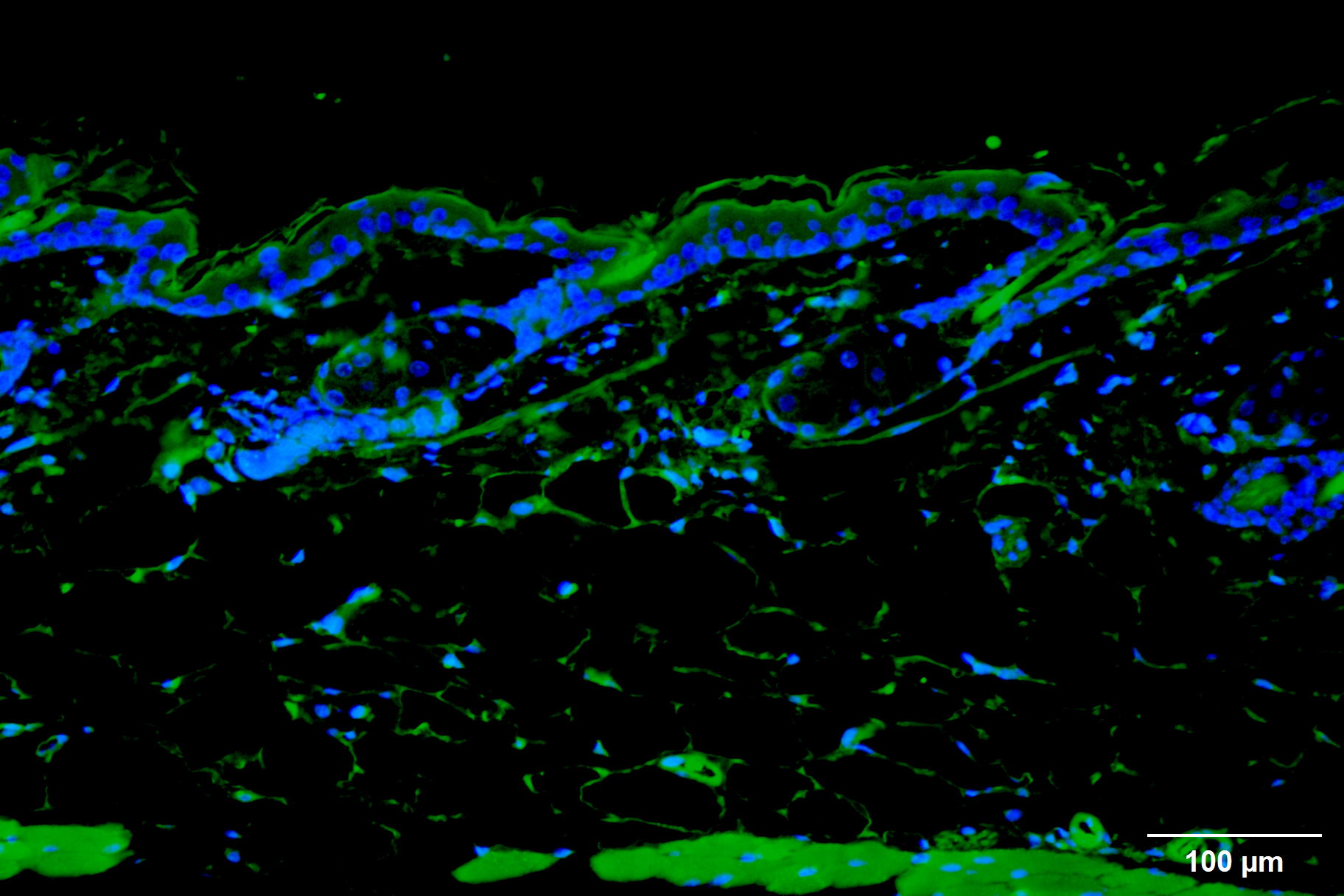

Supplement: Supplementary file 7 [file DataSheet7.zip › LA-Immunofluorescence staining image-Figure 5C/Figure 5C/1-3.tif]

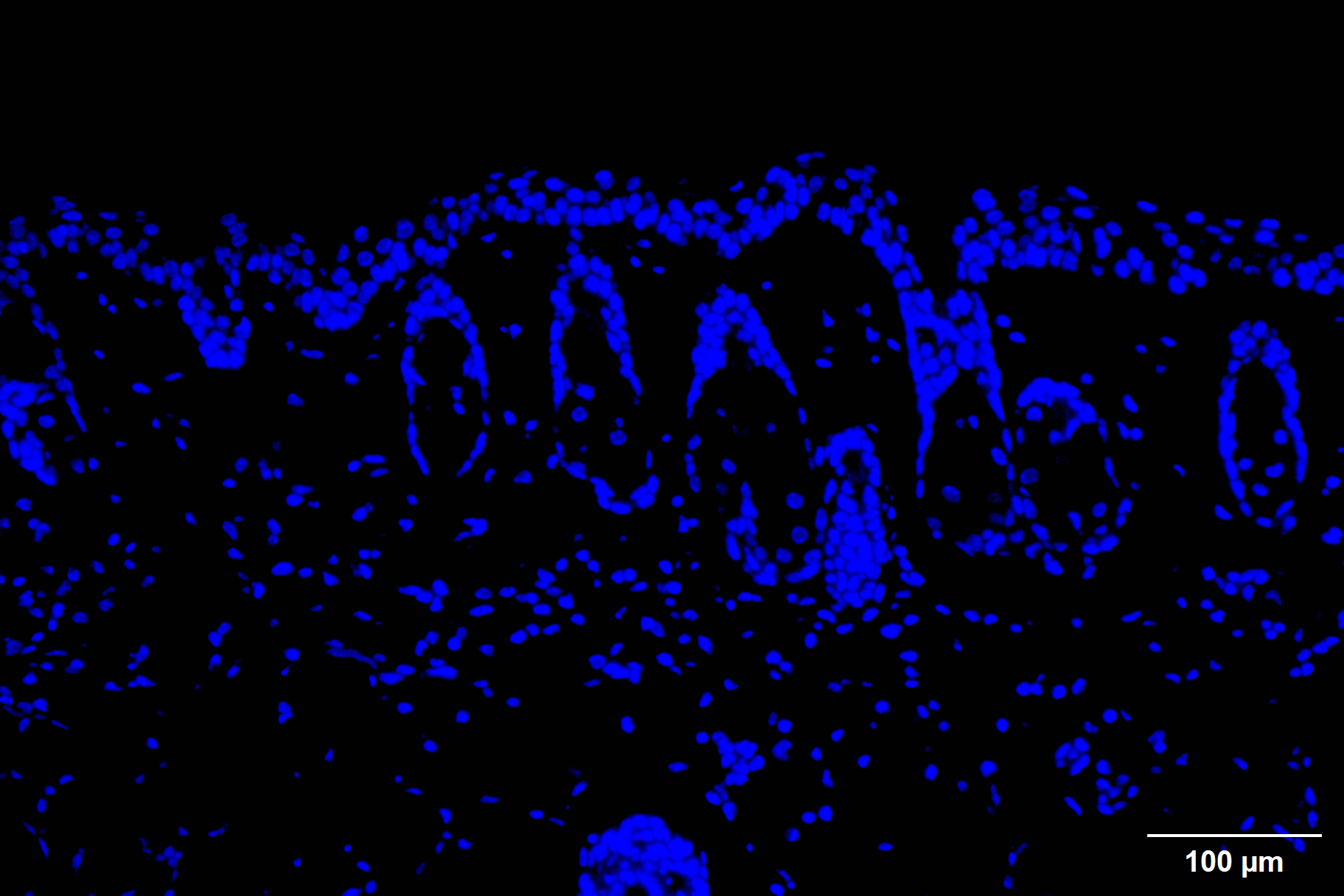

Supplement: Supplementary file 7 [file DataSheet7.zip › LA-Immunofluorescence staining image-Figure 5C/Figure 5C/2-1.tif]

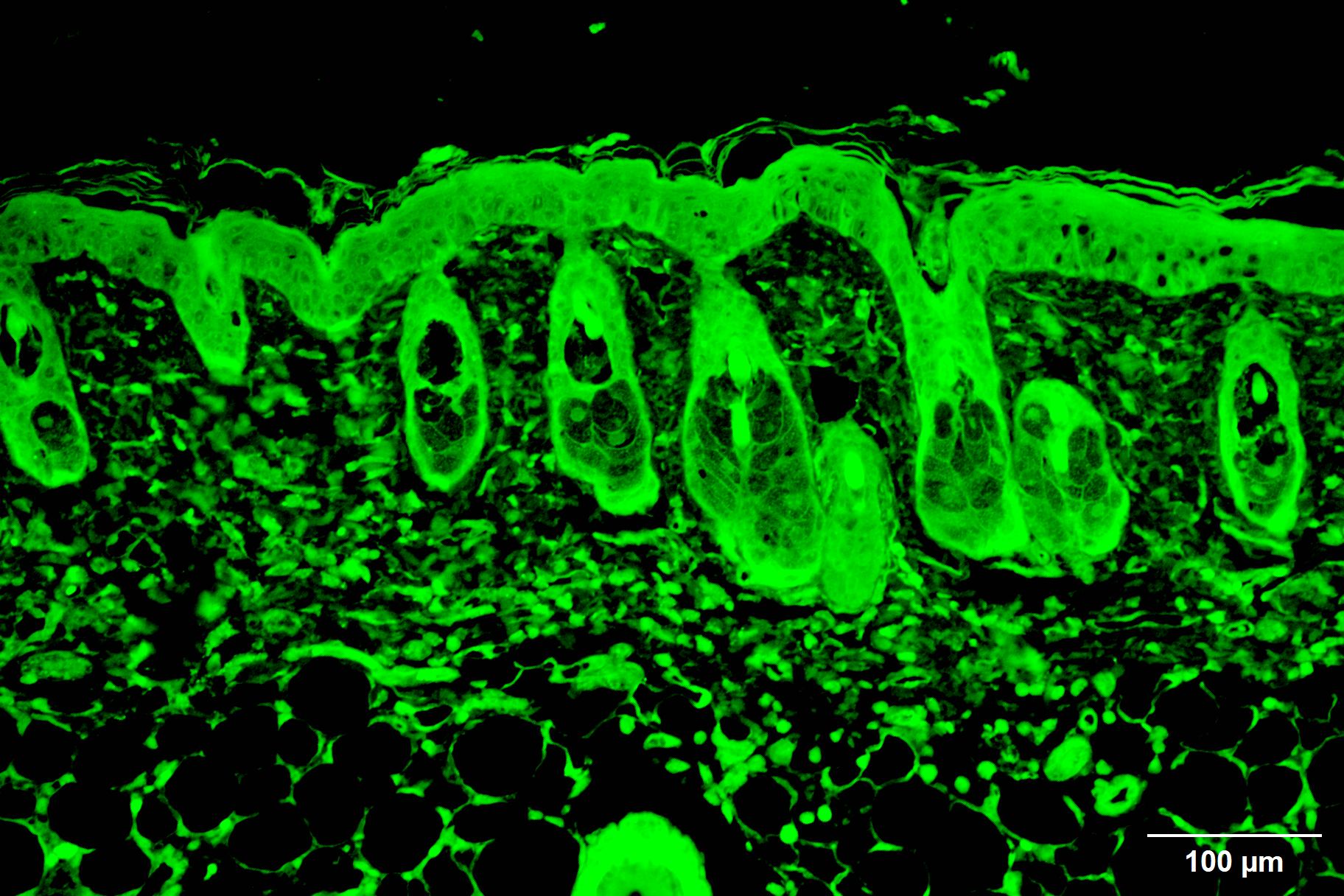

Supplement: Supplementary file 7 [file DataSheet7.zip › LA-Immunofluorescence staining image-Figure 5C/Figure 5C/2-2.tif]

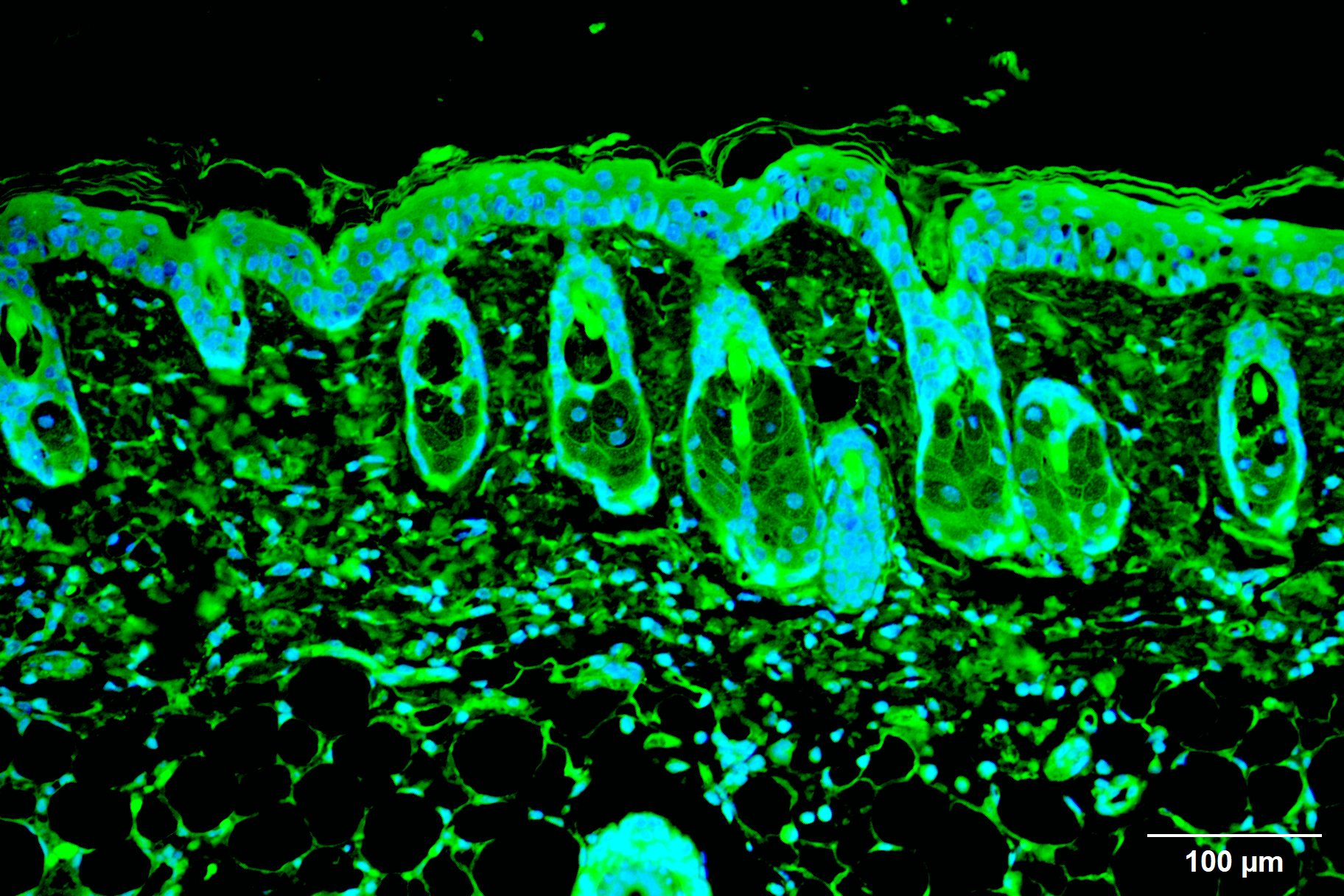

Supplement: Supplementary file 7 [file DataSheet7.zip › LA-Immunofluorescence staining image-Figure 5C/Figure 5C/2-3.tif]

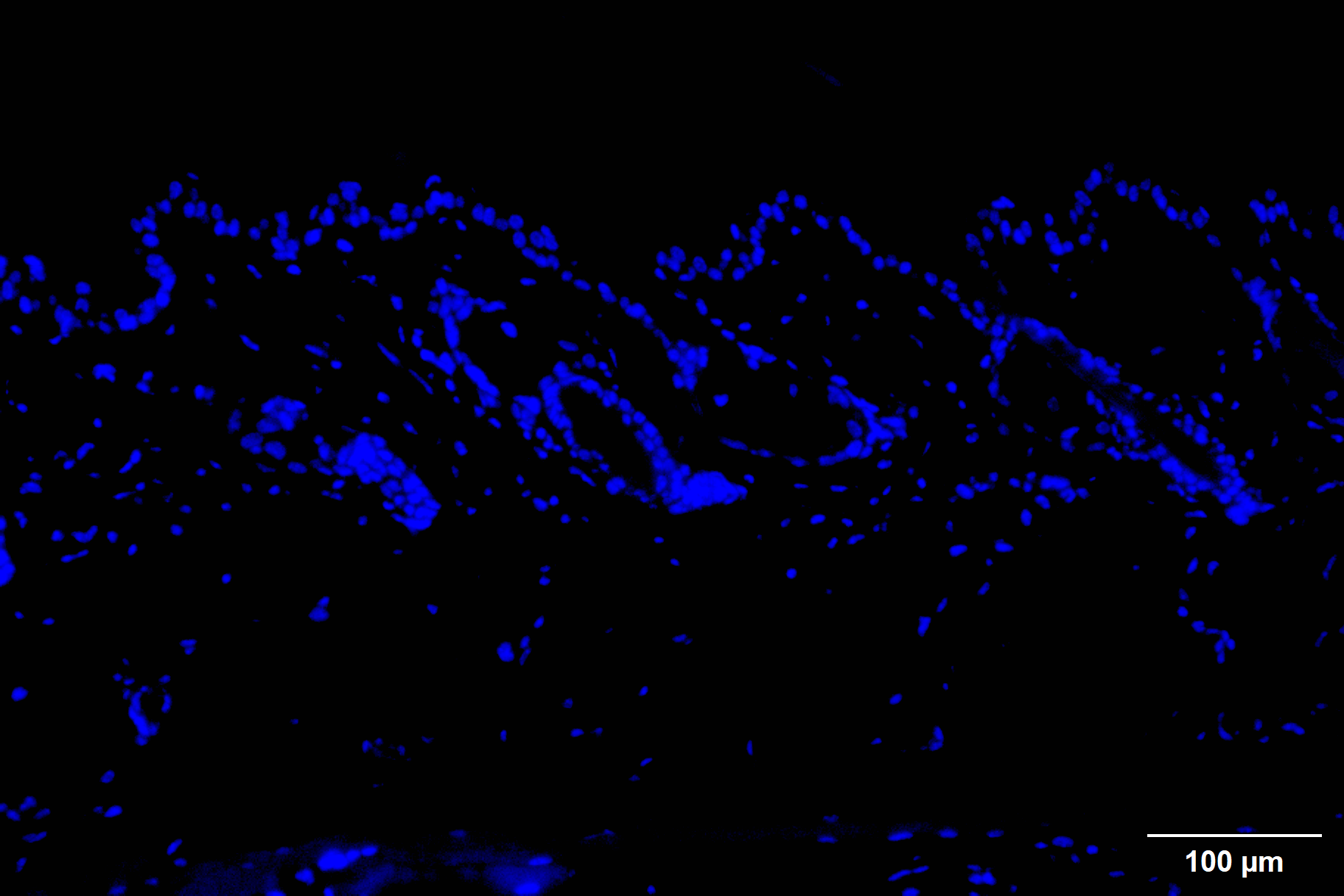

Supplement: Supplementary file 7 [file DataSheet7.zip › LA-Immunofluorescence staining image-Figure 5C/Figure 5C/3-1.tif]

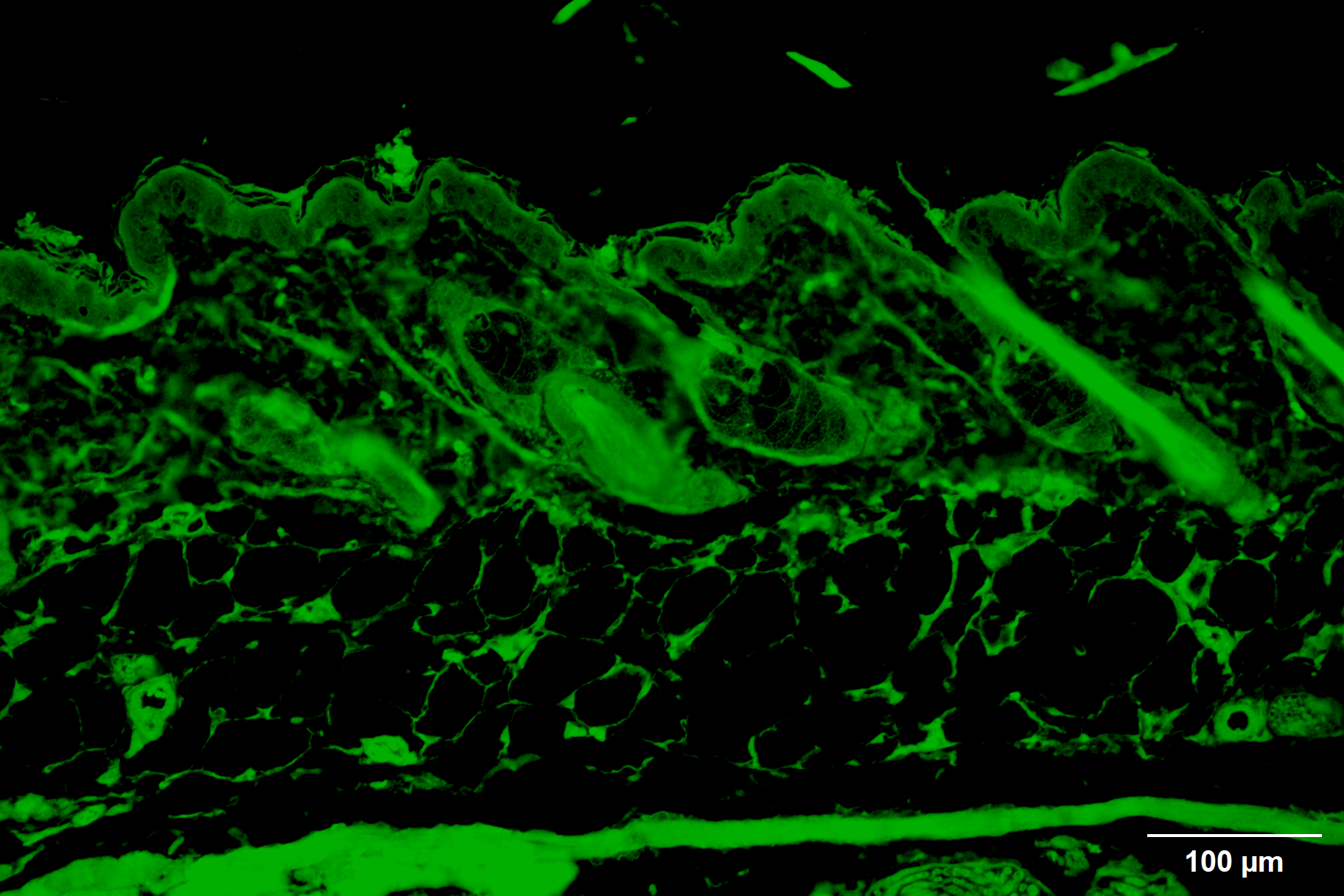

Supplement: Supplementary file 7 [file DataSheet7.zip › LA-Immunofluorescence staining image-Figure 5C/Figure 5C/3-2.tif]

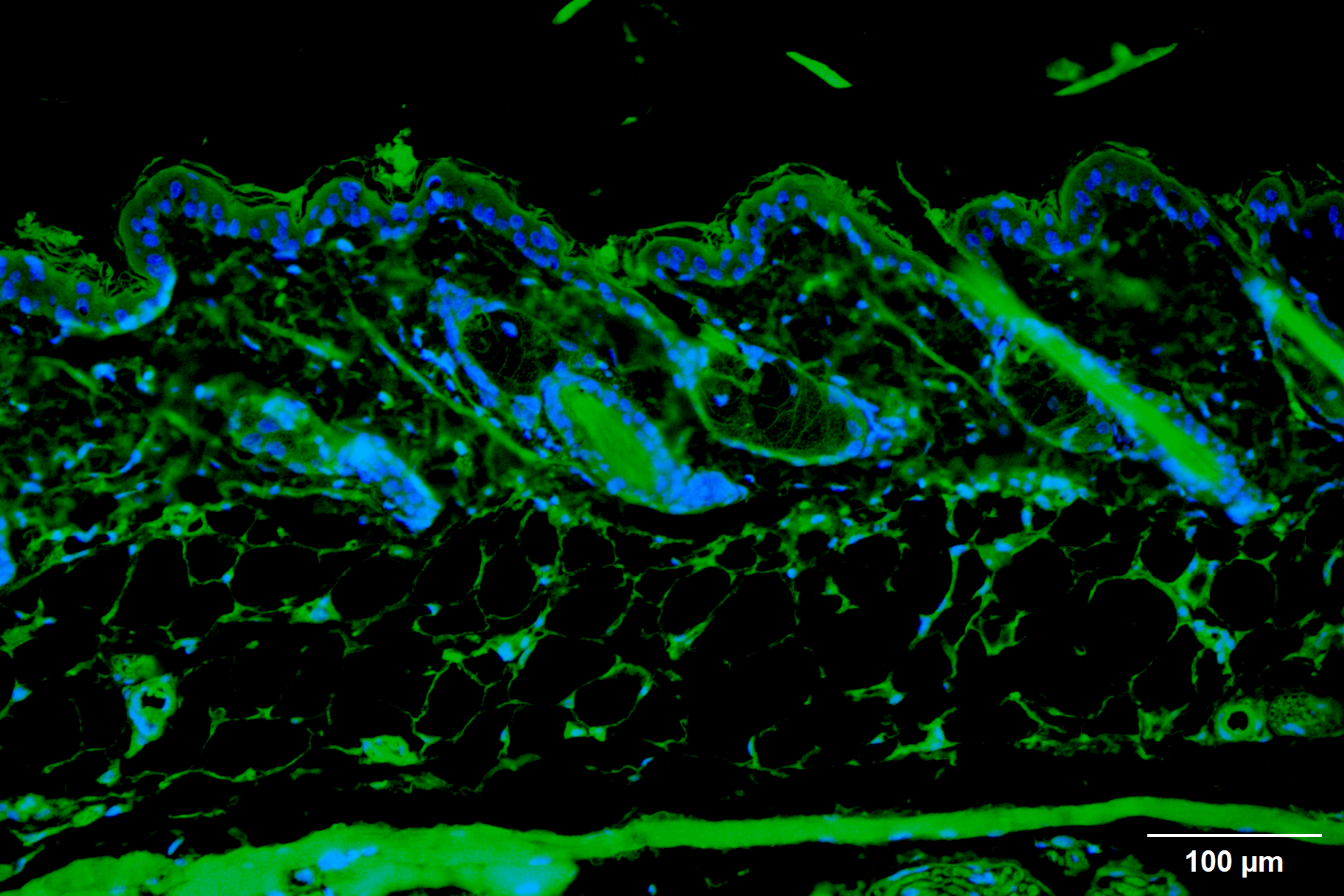

Supplement: Supplementary file 7 [file DataSheet7.zip › LA-Immunofluorescence staining image-Figure 5C/Figure 5C/3-3.tif]

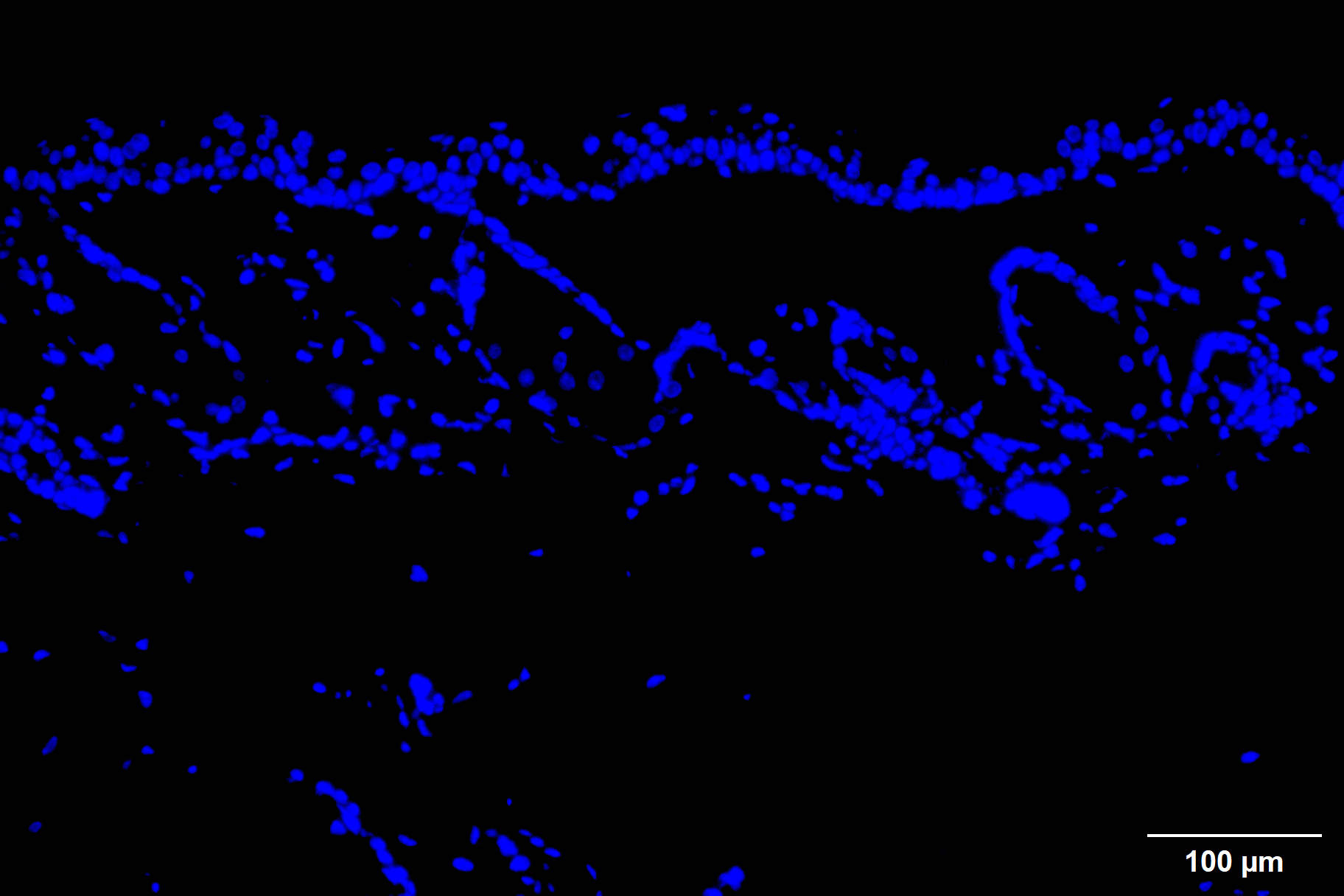

Supplement: Supplementary file 7 [file DataSheet7.zip › LA-Immunofluorescence staining image-Figure 5C/Figure 5C/4-1.tif]

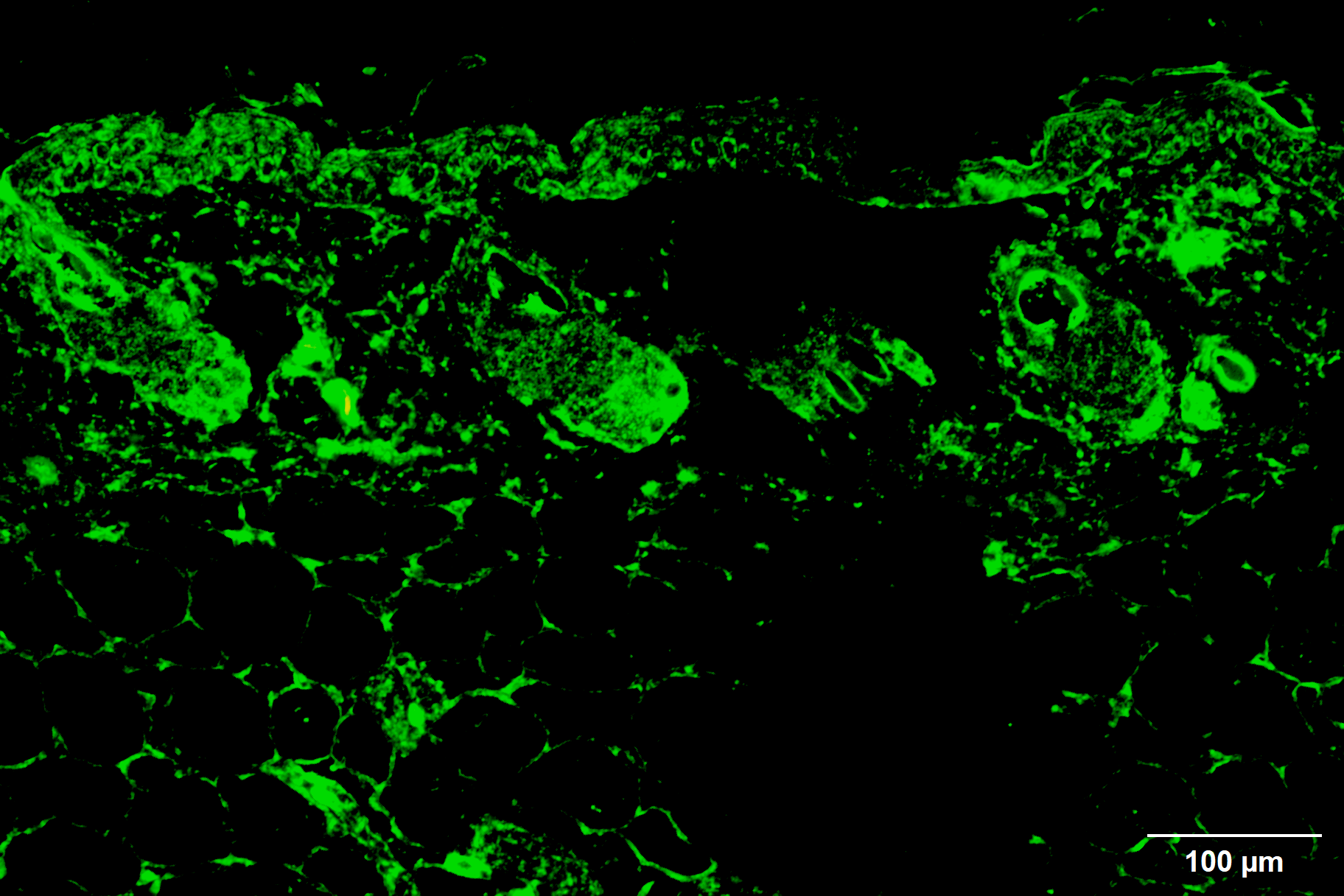

Supplement: Supplementary file 7 [file DataSheet7.zip › LA-Immunofluorescence staining image-Figure 5C/Figure 5C/4-2.tif]
