# Supplementary figures and images for: Lithospermic acid, a novel KLK5 inhibitor, ameliorates rosacea by suppressing the TLR4/NF-κB signaling pathway and rectifying phenylalanine metabolism (part 2 of 2)
Source: Front Immunol. 2026 Jan 26;17:1734997. doi: 10.3389/fimmu.2026.1734997 (PMC12883415; doi:10.3389/fimmu.2026.1734997)

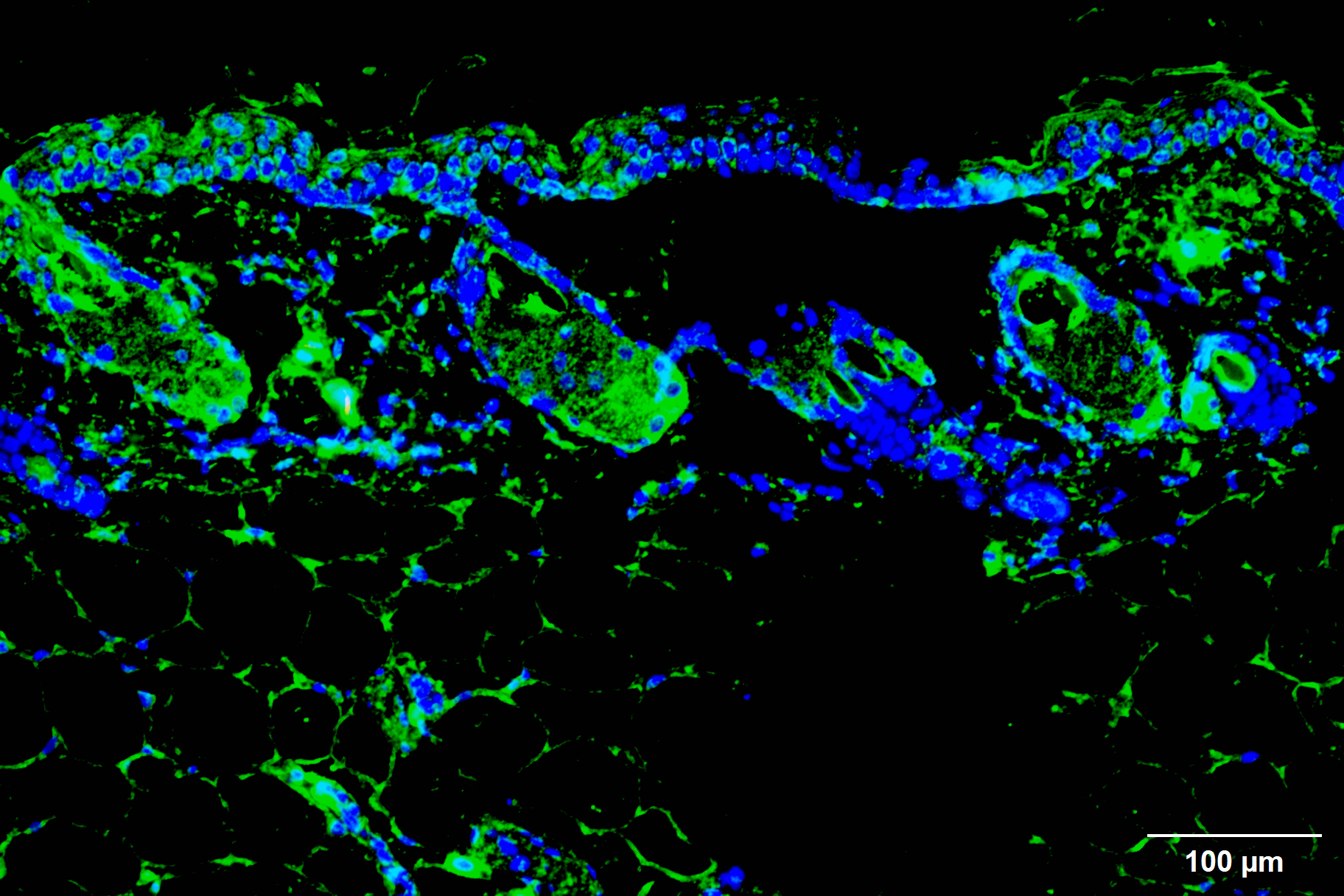

Supplement: Supplementary file 7 [file DataSheet7.zip › LA-Immunofluorescence staining image-Figure 5C/Figure 5C/4-3.tif]

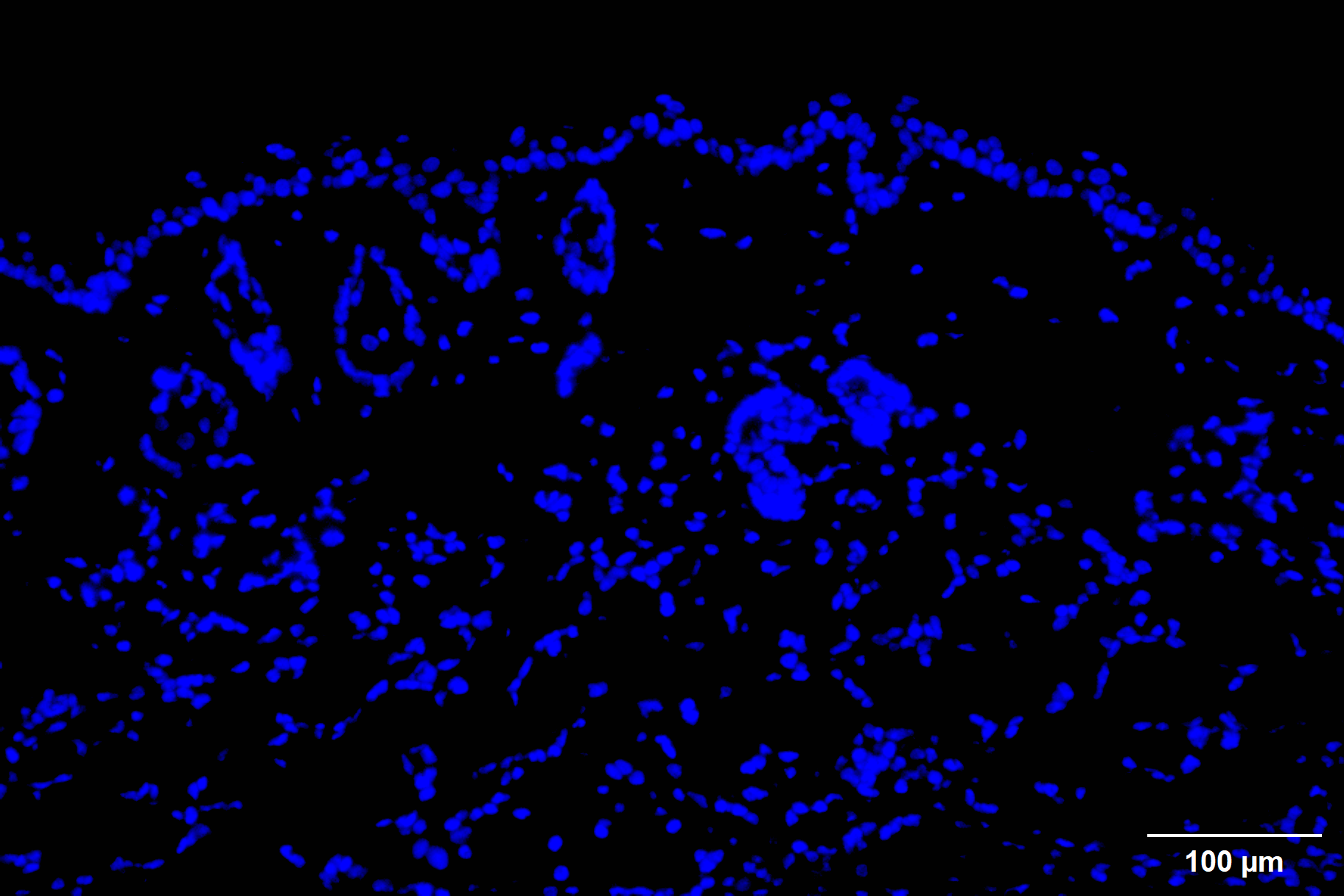

Supplement: Supplementary file 7 [file DataSheet7.zip › LA-Immunofluorescence staining image-Figure 5C/Figure 5C/5-1.tif]

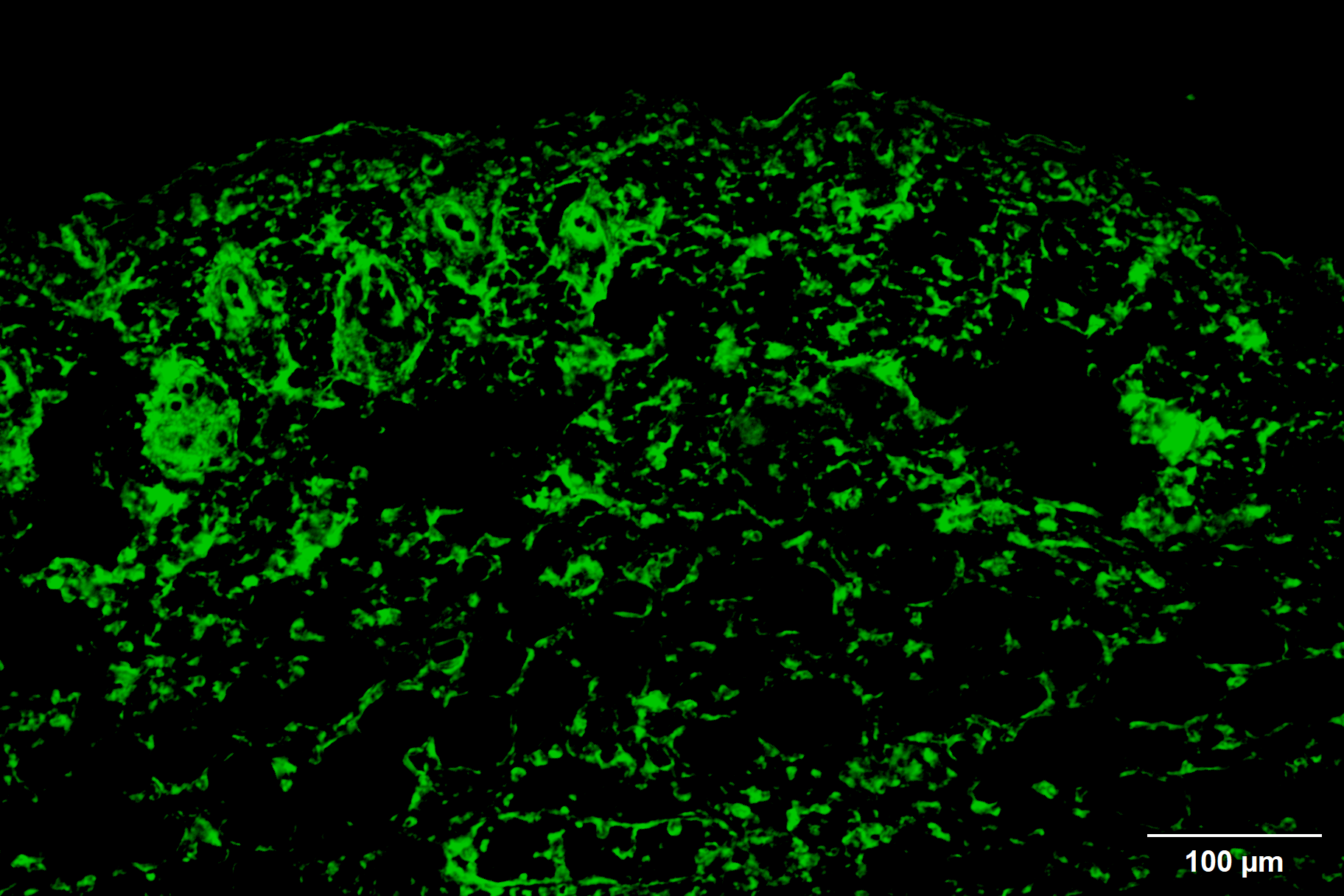

Supplement: Supplementary file 7 [file DataSheet7.zip › LA-Immunofluorescence staining image-Figure 5C/Figure 5C/5-2.tif]

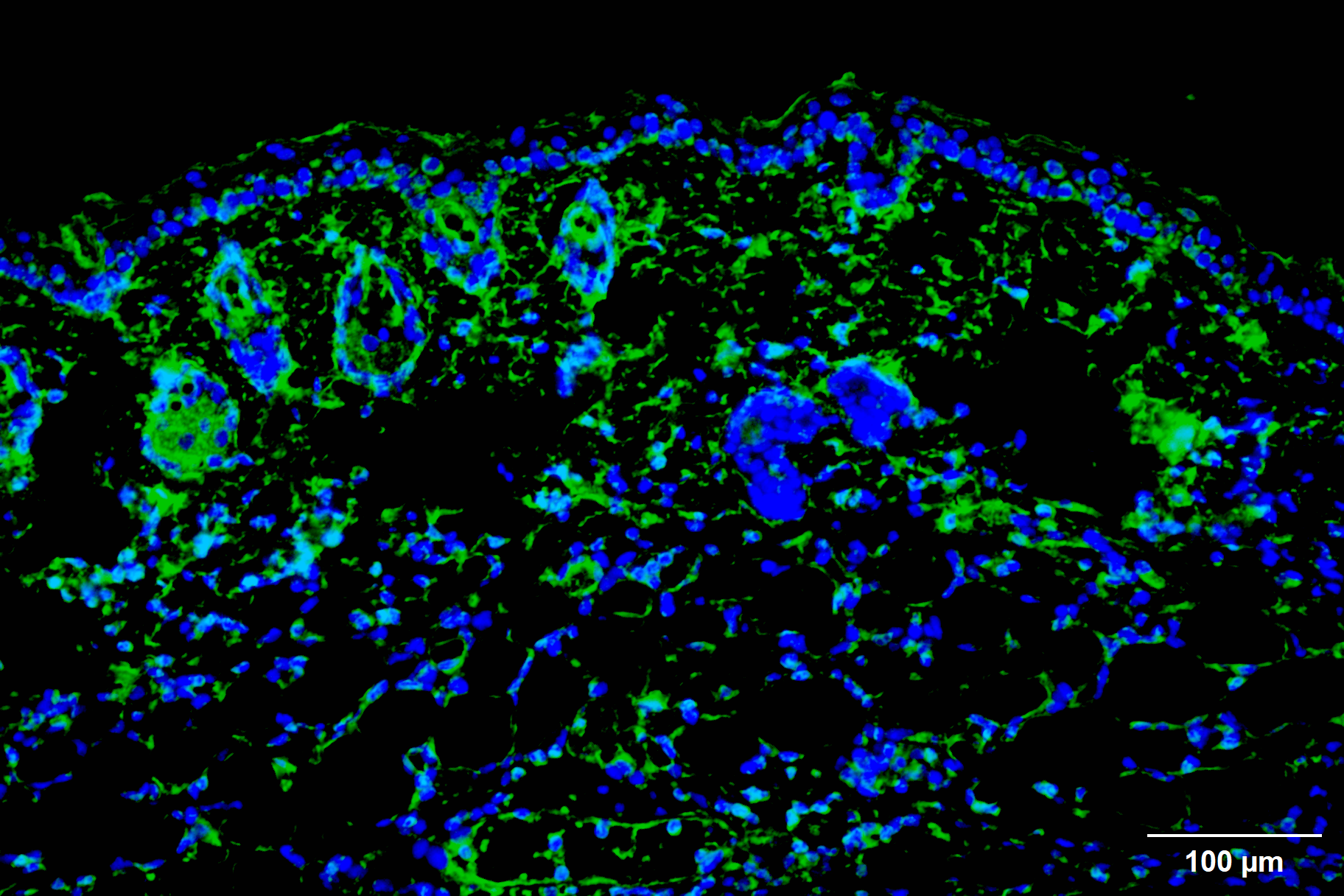

Supplement: Supplementary file 7 [file DataSheet7.zip › LA-Immunofluorescence staining image-Figure 5C/Figure 5C/5-3.tif]

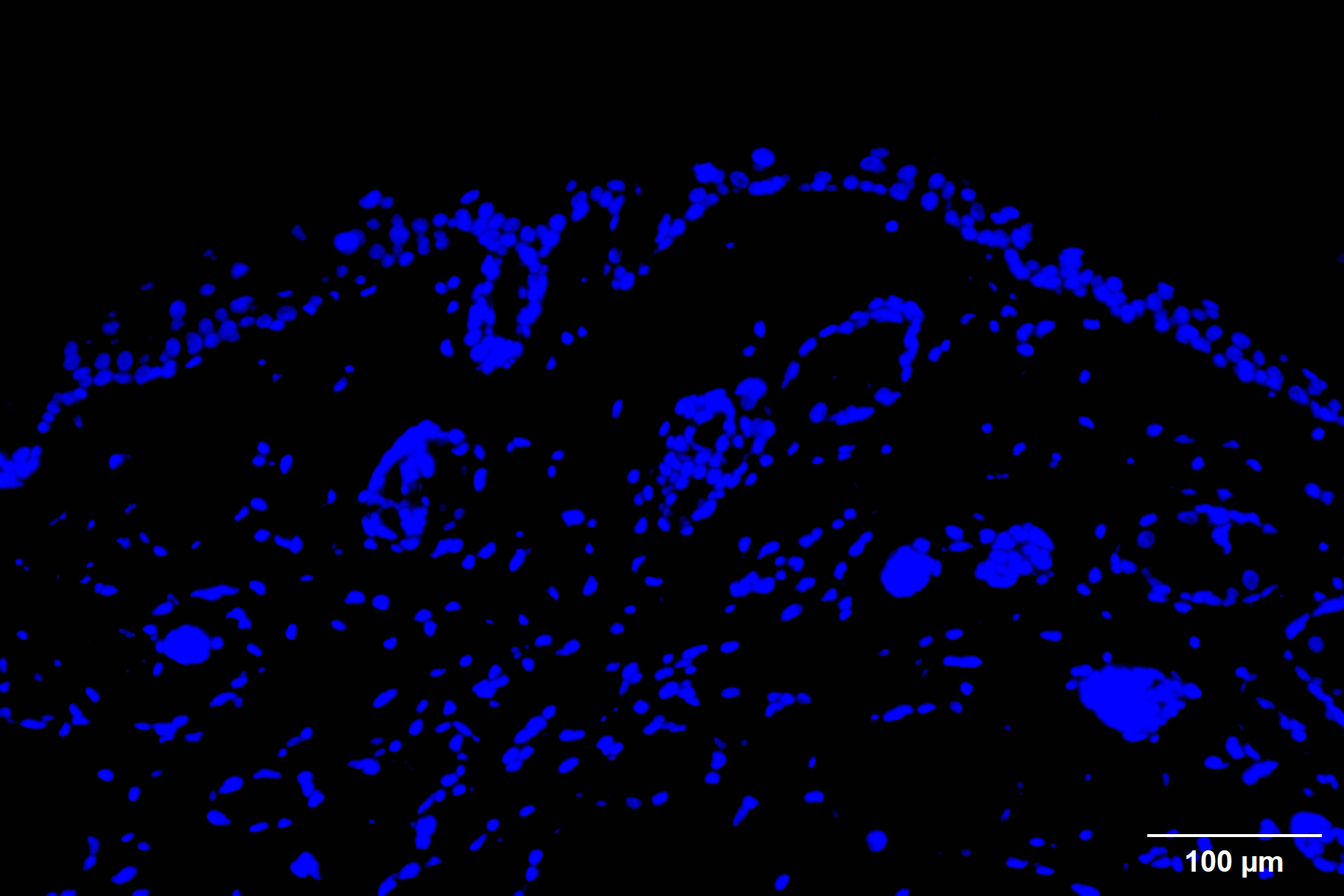

Supplement: Supplementary file 7 [file DataSheet7.zip › LA-Immunofluorescence staining image-Figure 5C/Figure 5C/6-1.tif]

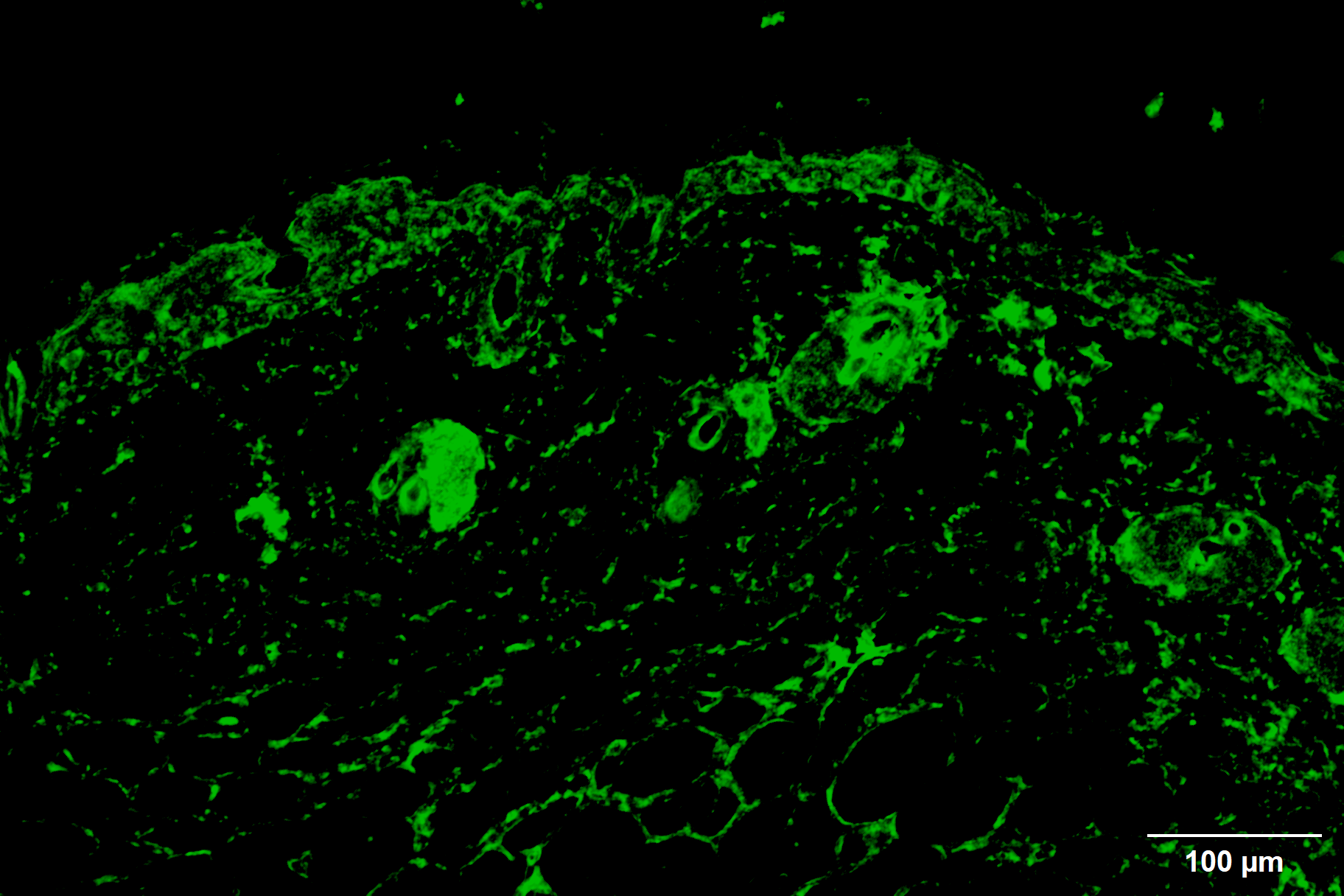

Supplement: Supplementary file 7 [file DataSheet7.zip › LA-Immunofluorescence staining image-Figure 5C/Figure 5C/6-2.tif]

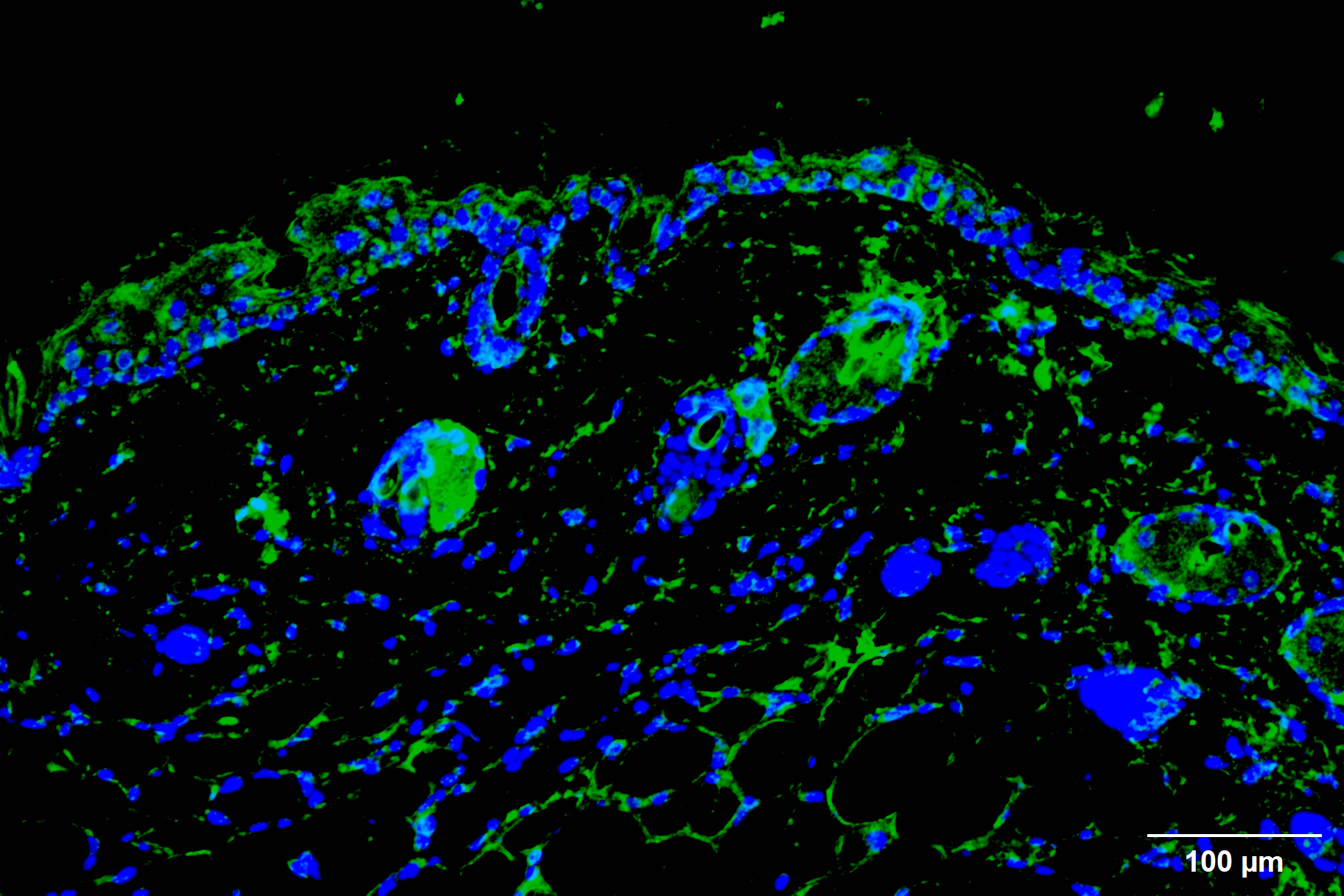

Supplement: Supplementary file 7 [file DataSheet7.zip › LA-Immunofluorescence staining image-Figure 5C/Figure 5C/6-3.tif]

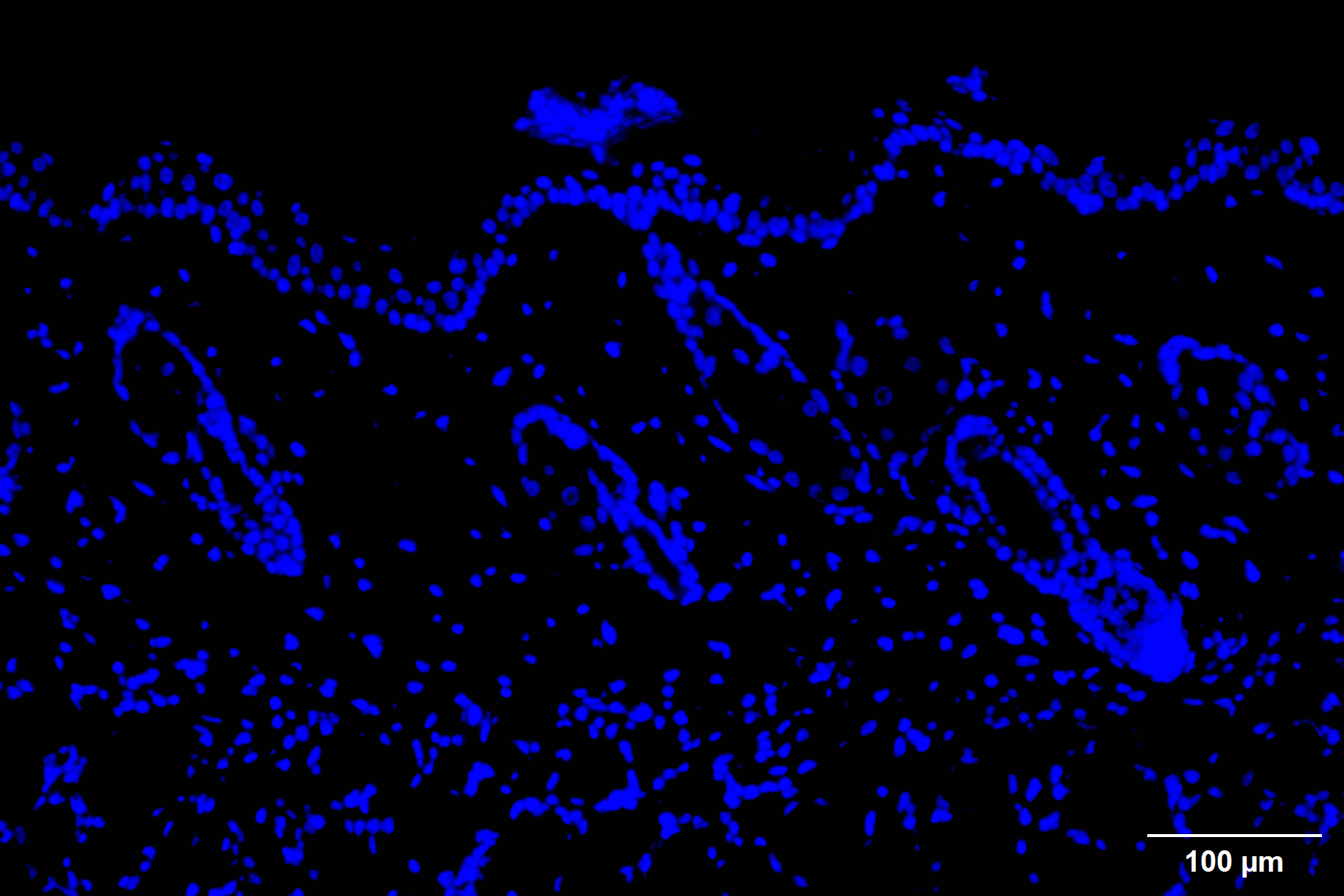

Supplement: Supplementary file 7 [file DataSheet7.zip › LA-Immunofluorescence staining image-Figure 5C/Figure 5C/7-1.tif]

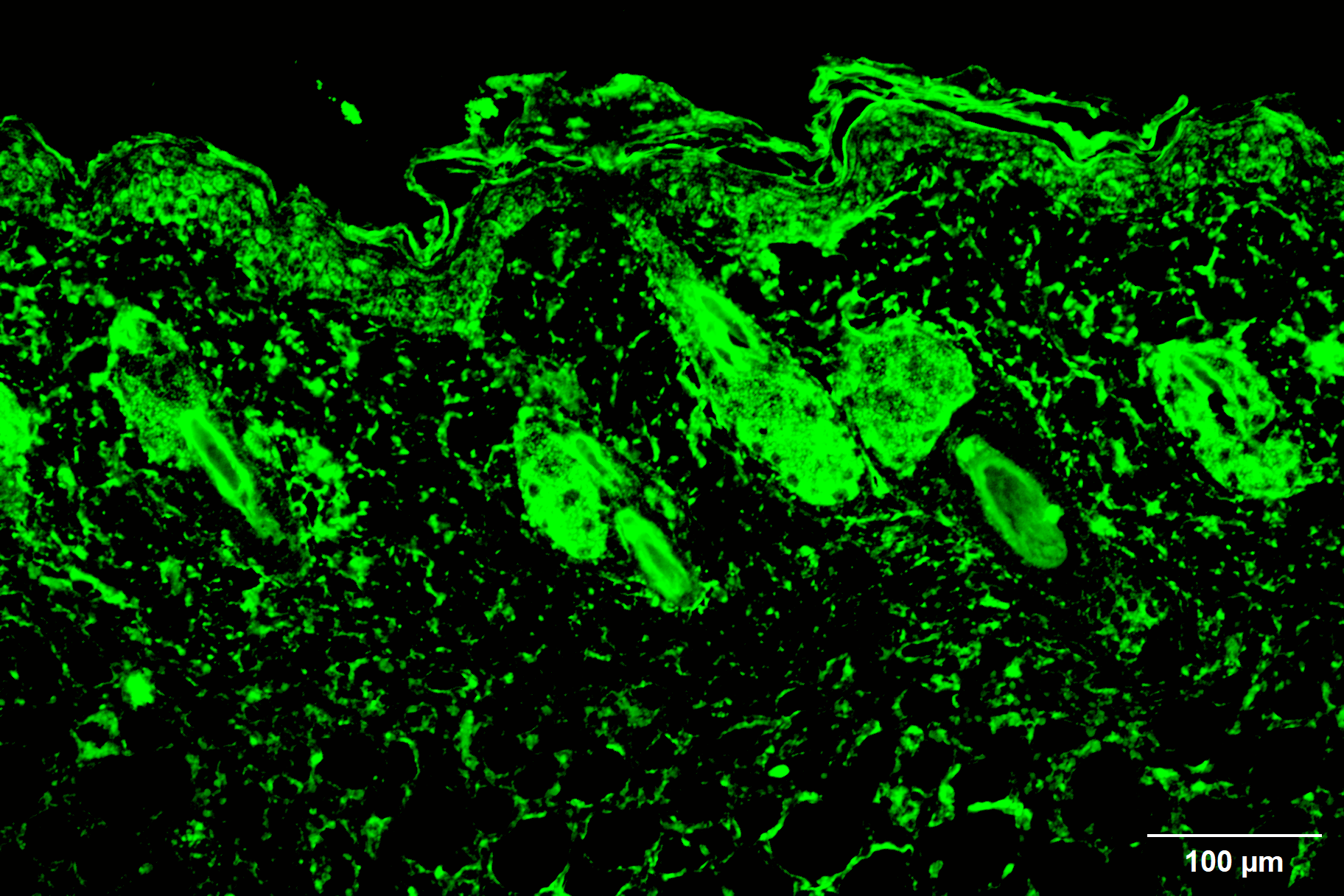

Supplement: Supplementary file 7 [file DataSheet7.zip › LA-Immunofluorescence staining image-Figure 5C/Figure 5C/7-2.tif]

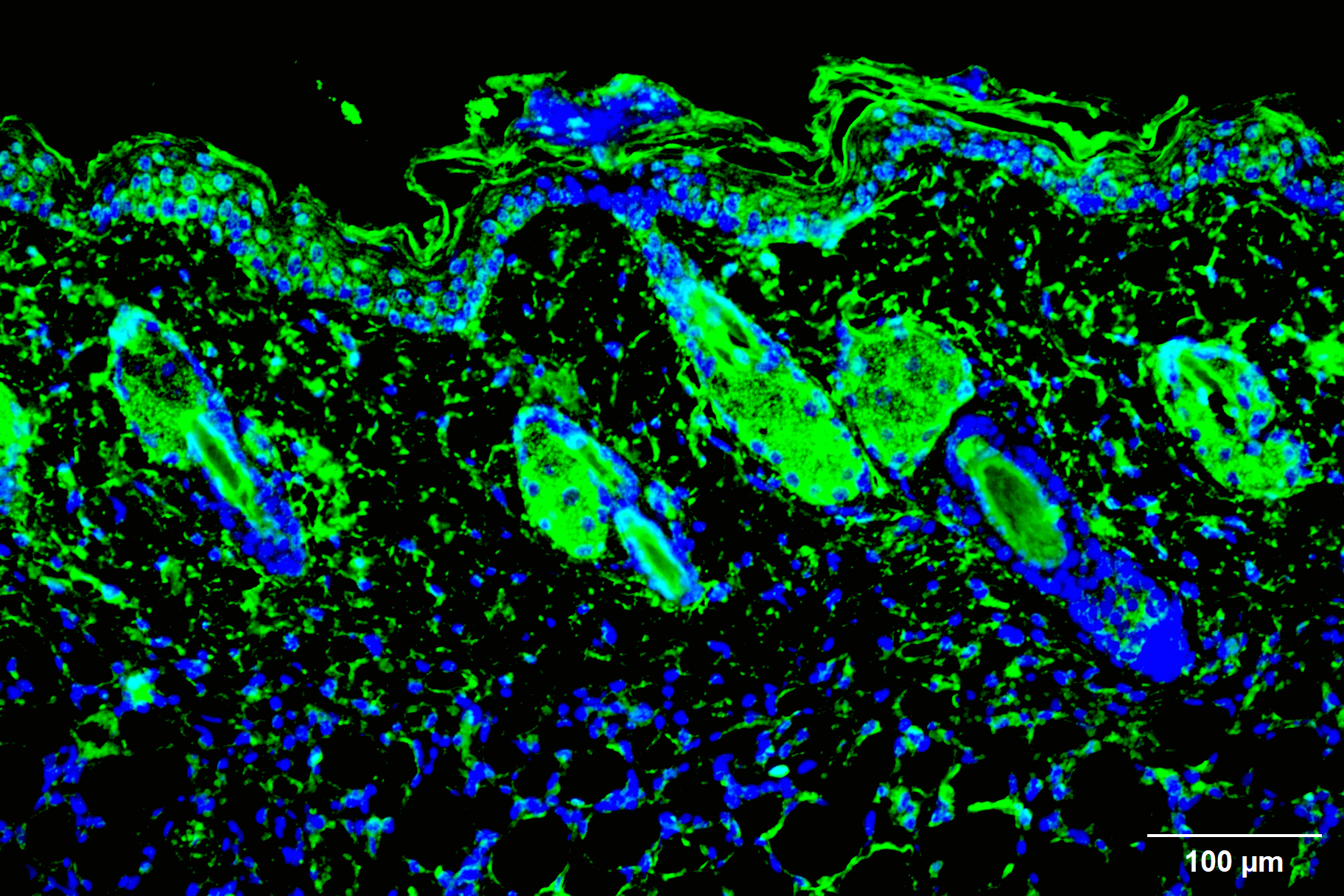

Supplement: Supplementary file 7 [file DataSheet7.zip › LA-Immunofluorescence staining image-Figure 5C/Figure 5C/7-3.tif]
